# Network-based identification of key master regulators associated with an immune-silent cancer phenotype

**DOI:** 10.1093/bib/bbab168

**Published:** 2021-05-13

**Authors:** Raghvendra Mall, Mohamad Saad, Jessica Roelands, Darawan Rinchai, Khalid Kunji, Hossam Almeer, Wouter Hendrickx, Francesco M Marincola, Michele Ceccarelli, Davide Bedognetti

**Affiliations:** Qatar Computing Research Institute, Hamad Bin Khalifa University, Doha, Qatar; Qatar Computing Research Institute, Hamad Bin Khalifa University, Doha, Qatar; Cancer Research Department, Research Branch, Sidra Medicince, Doha, Qatar; Cancer Research Department, Research Branch, Sidra Medicince, Doha, Qatar; Qatar Computing Research Institute, Hamad Bin Khalifa University, Doha, Qatar; Qatar Computing Research Institute, Hamad Bin Khalifa University, Doha, Qatar; Cancer Research Department, Research Branch, Sidra Medicince, Doha, Qatar; Refuge Biotechnologies, Menlo Park, California, USA; Department of Electrical Engineering and Information Technology (DIETI), University of Naples ”Federico II”, Via Claudio 21, 80215 Naples, Italy; Biogem, Istituto di Biologia e Genetica Molecolare, Via Camporeale, Ariano Irpino (AV); Cancer Research Department, Research Branch, Sidra Medicince, Doha, Qatar; Department of Internal Medicine and Medical Specialities, University of Genova, Genova, Italy; College of Health and Life Sciences, Hamad Bin Khalifa University, Doha, Qatar

**Keywords:** transcription regulator, master regulator analysis, immunologic constant of rejection, immune exclusion, gene regulatory networks

## Abstract

A cancer immune phenotype characterized by an active T-helper 1 (Th1)/cytotoxic response is associated with responsiveness to immunotherapy and favorable prognosis across different tumors. However, in some cancers, such an intratumoral immune activation does not confer protection from progression or relapse. Defining mechanisms associated with immune evasion is imperative to refine stratification algorithms, to guide treatment decisions and to identify candidates for immune-targeted therapy. Molecular alterations governing mechanisms for immune exclusion are still largely unknown. The availability of large genomic datasets offers an opportunity to ascertain key determinants of differential intratumoral immune response. We follow a network-based protocol to identify transcription regulators (TRs) associated with poor immunologic antitumor activity. We use a consensus of four different pipelines consisting of two state-of-the-art gene regulatory network inference techniques, regularized gradient boosting machines and ARACNE to determine TR regulons, and three separate enrichment techniques, including fast gene set enrichment analysis, gene set variation analysis and virtual inference of protein activity by enriched regulon analysis to identify the most important TRs affecting immunologic antitumor activity. These TRs, referred to as master regulators (MRs), are unique to immune-silent and immune-active tumors, respectively. We validated the MRs coherently associated with the immune-silent phenotype across cancers in The Cancer Genome Atlas and a series of additional datasets in the Prediction of Clinical Outcomes from Genomic Profiles repository. A downstream analysis of MRs specific to the immune-silent phenotype resulted in the identification of several enriched candidate pathways, including NOTCH1, TGF-$\beta $, Interleukin-1 and TNF-$\alpha $ signaling pathways. TGFB1I1 emerged as one of the main negative immune modulators preventing the favorable effects of a Th1/cytotoxic response.

## 1 Introduction

Over the past decade, corroborations of the effects of antitumor immunity on tumor progression have piled up. The discovery of escape mechanisms of the tumor-immune system, including the identification of immune checkpoints, has led to major advancements in immunotherapy [1–3]. Despite these advancements, a significant proportion of patients (60-80%) who are treated with immunotherapy still fail to obtain better clinical outcomes [1].

It is now accepted that a T-cell inflamed cancer phenotype is associated with responsiveness to immunotherapy and a favorable prognosis [3–6]. In this context, we previously defined the immunologic constant of rejection (ICR), a signature that captures the concomitant activation of innate and adaptive immune effector mechanisms required for the occurrence of immune-mediated tissue-specific destruction [9]. The ICR consists of 20 transcripts belonging to four functional categories: CXCR3/CCR5 chemokines (CXCL9, CXCL10, CCL5), Th1/Interferon-$\gamma $ signaling (IFNG, IL12B, TBX21, CD8A, CD8B, STAT1, IRF1), cytotoxic (GNLY, PRF1, GZMA, GZMB, GZMH) and immune regulatory (CD274, CTLA4, FOXP3, IDO1, PDCD1) functions [9, 23, 28, 50, 60, 62]. We have previously assessed the prognostic and predictive implications of ICR in different settings and used such a signature to define discrete categories of immune subtypes i.e. ICR high (ICR-H), ICR medium (ICR-M) and ICR low (ICR-L) within each solid tumor included in The Cancer Genome Atlas (TCGA) data [12, 49]. However, while prognostic and predictive connotations of the intratumoral immune response have been extensively addressed, the mechanisms governing the molecular alterations underlying immune exclusion are poorly understood. Thus, it is imperative to identify key driver genes and their associated downstream mechanisms leading to immune exclusion to develop effective therapeutic strategies [50].

Using the TCGA database, pan-cancer analyses have sought to address this critical question by correlating the mutational status of driver cancer genes and/or the status of oncogenic signals with the degree of the intratumoral immune response [7, 8, 16, 19, 49, 59]. While some of the oncogenic signals have been validated in experimental models [34, 56], a considerable proportion of intratumoral immune response variation remains unexplained [8]. It is presently unknown whether an intrinsic activation of transcription regulators (TRs) involved in sustaining the oncogenic process can influence distinct immune disposition and their prognostic implications, and this represents our working hypothesis.

A necessary condition for tumor progression and drug resistance is transcriptional dysregulation [24, 32]. A majority of cancer driver genes are TRs [20]. TRs are largely dysregulated due to genomic aberrations or alterations in their regulatory proteins, which in return can modulate the expression of their target genes, referred to as its ‘regulon’. These TRs have been identified as key oncogenic drivers whose activity patterns are influential to a patient’s clinical prognosis [21].

Here we use the TCGA RNA-Seq data to discover key driver TRs, referred to as master regulators (MRs), for the immune-silent cancer phenotype. We utilize the RNA-Seq data for $12$ cancer types, comprising a total of $2307$ primary tumor samples divided into ICR low (ICR-L and immune silent) and ICR high (ICR-H and immune active) [49], each having gene expression for $3674$ TRs and $23\,216$ target genes. These $12$ cancers include 8 tumor types in which ICR bears a favorable prognostic implication referred as ICR enabled (IRC-E) tumors and 4 cancer types in which ICR is associated with unfavorable prognosis, namely ICR disabled (ICR-D) tumors as illustrated in [49].

There have been several methods in the literature [15, 38] that have been used in previous studies to perform MR analysis (MRA). A primary ingredient for MRA is to reverse engineer a high quality gene regulatory network (GRN) consisting of TR-target gene interactions (regulon or gene sets) from RNA-Seq data. This is one of the central problems in computational biology, and a plethora of techniques have been proposed, including mutual information-based method ARACNE [37] and tree-based machine learning techniques such as GENIE [33] and regularized gradient boosting machine (RGBM) [17, 22, 41–44, 51]. In [45], through an open-science competition (DREAM Challenge), the authors compared various GRN inference methods on several synthetic and real datasets. In [26], the authors illustrated the superior performance of RGBM for the DREAM Challenge networks (see Supplementary Figure S1b). Hence, RGBM is the primary GRN inference technique focused on in this work.

Another key component of MRA is to estimate enrichment/activity scores for TRs in a given sample, taking into consideration its regulon. This is essential to identify differentially enriched/activated TRs (MRs). While techniques such as RGBM utilize a simplistic difference in average expression of positively and negatively regulated targets to estimate the activity of a TR, methods such as virtual inference of protein-activity by enriched regulon analysis (VIPER) [8] and MARINA [6] utilize a dedicated algorithm formulated to estimate TR activity taking into account the TR mode of action, the TR-target gene interaction confidence and the pleiotropic nature of each target gene regulation. Moreover, there exists single sample gene set enrichment analysis [57] techniques such as gene set variation analysis (GSVA [27]) and fast gene set enrichment analysis (FGSEA [53]) to estimate enrichment score for each TR in a given sample. This is utilized for further differential analysis (ICR-H versus ICR-L) to identify the key MRs w.r.t. a phenotype of interest.

In recent literature, techniques such as Netfactor [8] and [24] take a consensus based approach to identify signature specific MRs or estimate TR activities, respectively. It was shown in [24] that since the TR regulons are estimated by taking a consensus approach, they are more robust for downstream tasks with less likely to be influenced by false positives. Following the same principle, we identify the MRs specific to immune-active and immune-silent cancer phenotypes by taking a consensus (intersection) of the MRs determined by using four different MRA pipelines: (a) RGBM + FGSEA, (b) RGBM + GSVA, (c) RGBM + VIPER and (d) ARACNE + VIPER. Thus, in our proposed framework, we use two state-of-the-art GRN inference techniques and three different gene set enrichment/activity estimation techniques to robustly determine the MRs.

We investigate the MRs that are common across $12$ cancer types and are specific to either the ICR-H or ICR-L phenotype. We perform a validation of these MRs by observing expected activity patterns (with statistical significance) for ICR-H and ICR-L samples in each of the remaining cancer types in TCGA. These cancer types, referred as ICR neutral (ICR-N), have no clear correlation between the immunological status and prognosis [49] and thus serve as a test set for pan-cancer validation. Furthermore, in another replication study, we observe expected activity patterns for MRs specific to either ICR-H or ICR-L phenotype on a set of eight different datasets (cancer types) in the Prediction of Clinical Outcomes from Genomics Profiles (PRECOG) repository [25]. Finally, we perform downstream analysis of the MRs specific to ICR-L using ConsensusPathDB [35] to discover corresponding enriched pathways, several of which are potential candidates that can be targeted to readjust the immunosuppressive tumor microenvironment. The primary contributions of our work are as follows:

$\bullet $ A framework which takes the consensus of four different MRA pipelines to identify MRs specific to immune-active and immune-silent phenotype.

$\bullet $ Validation of the activities of MRs specific to ICR-L and ICR-H on sets from two different data sources i.e. the ICR-N cancer types in TCGA and the PRECOG datasets.

$\bullet $ Downstream analysis of MRs specific to ICR-L lead to the unbiased identification of enriched pathways, such as the NOTCH1, TGF-$\beta $, Interleukin-1 and TNF-$\alpha $ signaling pathways, which can potentially be targeted to readjust the immunosuppressive tumor microenvironment.

$\bullet $ TGFB1I1 emerged as one of the main negative immune modulators preventing the favorable effects of a Th1/cytotoxic response.

**Fig. 1:**
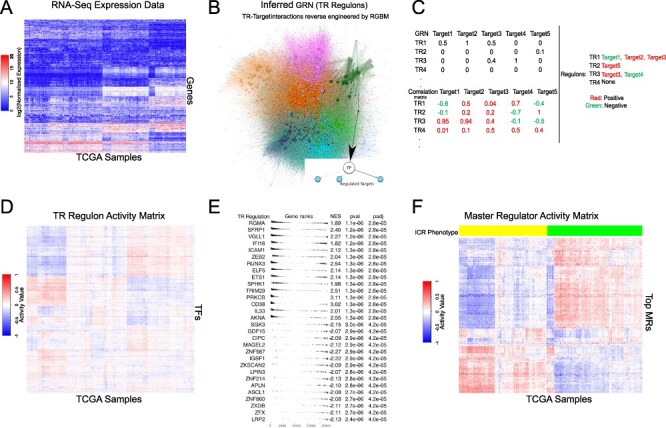
Identification of differentially active MRs for the ICR phenotype using one of the four master regulator analysis pipelines (RGBM + FGSEA). (A) RNA-Seq gene expression in the TCGA samples for one cancer, for example, breast invasive carcinoma (BRCA) was processed to be quantile-normalized and log2 transformed. (B) GRN inferred from the RNASeq data using RGBM for a given cancer. The GRN estimated TR-target interactions (see Supplementary Table S1 for details about TR-target network characteristics). (C) Correlation matrix determined using RNA-Seq and GRN, leading to the quantification of positively and negatively regulated targets for each TR. (D) The single-sample TR activities obtained from gene expression data and the TR regulons. (E) Top statistically significant ($P$$\le $ 0.05) differentially active TRs, referred to as MRs, determined using the FGSEA technique. (F) Activity matrix corresponding to the MRs for a cancer type along with the encoded phenotype information. Here ‘yellow’ samples refer to ICR high phenotype whereas the ‘green’ samples belong to ICR low phenotype.

Figure 1 illustrates the protocol followed for RGBM + FGSEA (one of the four consensus methods) to identify the MRs from the RNA-Seq data (see Supplementary Figure S1a) differentiating ICR-H from ICR-L phenotype. Table 1 represents the notation table for all the abbreviations used in this work.

**Table 1:** List of notations and abbreviations used

TR	Transcription regulator
MR	Master regulator
GRN	Gene regulatory network
ICR	Immunologic constant of rejection
NES	Normalized enrichment score
MRA	Mater regulator analysis
TCGA	The Cancer Genome Atlas
PRECOG	Prediction of Clinical Outcomes for Genomics profiles
RGBM	Regularized gradient boosting machine
GSEA	Gene set enrichment analysis
FGSEA	Fast gene set-enrichment analysis
GSVA	Gene set variation analysis
VIPER	Virtual inference of protein activity by enriched regulons
ICR-H	ICR high $\rightarrow $ highest expression of ICR genes $\rightarrow $ immune-active phenotype
ICR-L	ICR low $\rightarrow $ lowest expression of ICR genes $\rightarrow $ immune-silent phenotype
ICR-M	ICR medium $\rightarrow $ medium expression of ICR genes
ICR-E	ICR enabled cancers or eight cancer types in which ICR-H has favorable prognosis in terms of clinical outcome [49]
ICR-D	ICR disabled cancers or four cancer types in which ICR-L has favorable prognosis in terms of clinical outcome [49]
ICR-N	ICR neutral cancers (20 cancer types) that have no clear correlation between ICR and prognosis [49]
ICR-EH	ICR enabled cancer and ICR high sample within that cancer
ICR-EL	ICR enabled cancer and ICR low sample within that cancer
ICR-DH	ICR disabled cancer and ICR high sample within that cancer
ICR-DL	ICR disabled cancer and ICR low sample within that cancer

## 2 Materials and methods

### Transcriptional regulators

We wanted to select a wide list of candidates as TRs, looking for all the genes involved in the process that modulate the frequency, rate or extent of cellular DNA-templated transcription. Therefore, we selected all the genes annotated with the Gene Ontology (GO) term GO:0006355 (regulation of transcription) [5]. The interrogation of the GO through Ensembl Biomart was performed in August $2018$ resulting in a list of $3674$ TRs. Previously tools such as ARACNE, VIPER and NetFactor focused on transcription factors (TFs). Moreover, the original RGBM algorithm also exploited an active binding network based on binding sites of TFs. Recently, there have been studies [14, 47, 48] that extend the hubs of GRNs to regulatory proteins beyond the TFs. For example, in [48], the authors considered a set of $2506$ regulatory proteins annotated in GO with TF activity and transcription cofactor activity. Our set of $3674$ TRs was a superset of their set including receptors, kinases, growth factors, signal transduction proteins, transcription co-activators and cofactors as candidate regulators.

Other interesting examples where hubs of the networks were focused on signal molecules (and not just TFs) include approach such as SigMaps [14] or surface receptors i.e. the receptors interactome, to identify active ligand-receptors pairs [47]. These studies used ARACNE + VIPER and generalized the concept of MRA to generic signal molecules (not just TFs) as originally intended in [6, 37].

### Data acquisition and normalization

RNA-Seq data from the TCGA website were downloaded and processed using TCGA biolinks. RNA-Seq data for each cancer were represented as $\mathcal{D}^{c}=\{g_{1,i}^{c}, g_{2,i}^{c}, \ldots , g_{p,i}^{c}\}$, $ \forall i \in \{1, \ldots , N_{c}\}$, where $c$ represents the cancer type, $i$ corresponds to the $i$th sample, $g_{j,i}^{c}$ refers to the expression of the $j$th target gene in sample $i$ and $N_{c}$ represents the total number of samples available for that $c$. We had a total of $p$ = $23\,216$ target genes, including $3674$ TRs. The RNA-Seq data from 32 primary solid tumor cancers were used in our analysis. These samples were quantile normalized and $\log{2}$ transformed for analysis (see Figure 1A).

From the PRECOG repository, we selected 8 datasets, each corresponding to a different and the largest unique dataset available for a particular cancer type as the validation set. These included GEO Accession Id: GSE32894 for bladder urothelial carcinoma (BLCA), GSE3494 for breast invasive carcinoma (BRCA), GSE39582 for colon adenocarcinoma (COAD), GSE108474 for glioblastoma multiforme (GBM), GSE65858 for head and neck squamous cell carcinoma (HNSC), GSE72094 for lung adenocarcinoma (LUAD), GSE9891 for ovarian serous cystadenocarcinoma (OV) and GSE65904 for skin cutaneous melanoma (SKCM). Each of these $8$ datasets consisted of 224, 251, 579, 490, 270, 398, 278 and 210 tumor samples, respectively, and were normalized using ‘rma’ or quantile normalization [13], followed by $\log{2}$ transformation, depending on the platform i.e. Affymetrix and Illumina, respectively. These normalized datasets along with ICR information for each sample within a cancer type were obtained from [61].

### ICR classification and cancer type selection

Gene signatures found in early studies on tumor rejection by immunotherapy strongly overlap with pathways that were upregulated during other instances of immune-mediated tissue rejection like graft versus host disease, allograft rejection or autoimmunity [63]. This observation led to the formulation of the ICR. More specifically, ICR reflects coordinated activation of IFN-stimulated genes, upregulation of specific chemokine ligands, Th1 polarization and induction of immune effector functions, paralleled by the counter-activation of immune regulatory mechanisms [23, 28, 49, 50].

We previously classified the cancer samples in the TCGA using ICR classification [49] for each cancer type $c$. In short, a consensus clustering [49] algorithm based on the expression of the $20$ ICR genes [49] was applied to the cancer samples of a particular cancer $c$, to classify its samples into three discrete categories: ICR high (ICR-H and immune active i.e. hot immune phenotype), ICR medium and ICR low (ICR-L and immune silent i.e. cold immune phenotype).

The cluster with the highest expression of ICR genes was termed ICR-H, while the cluster with the lowest ICR gene expression was termed ICR-L. All samples in the intermediate cluster were defined as ICR medium (ICR-M) for each cancer, $c$, as indicated in [49]. The code and instructions to obtain the ICR class labels for all tumor samples of a specific cancer, $c$, are available in our repository https://github.com/raghvendra5688/ICR_Analysis (see Supplementary Table S2 for a breakdown of the tumor samples into the ICR-H, ICR-M and ICR-L categories for each cancer of interest). Our objective was to compare the cancer samples with a highly active immune phenotype i.e. ICR-H with the immune-silent phenotype i.e. ICR-L. In [49], we emphasized that there exists a subset of cancer types where ICR-H has a better survival prognosis than ICR-L. These eight cancer subtypes were referred to as ICR enabled (ICR-E) cancers. Similarly, there exists a subset of four cancer types for which the ICR-L group has better survival prognosis than ICR-H group, which we defined as ICR disabled (ICR-D) cancers. All remaining cancers were classified as ICR neutral (ICR-N). Thus, in this work, we primarily focused on these 12 cancer types (8 ICR-E and 4 ICR-D cancer types) as the ICR phenotype has prognostic value in these cancer types [49].

### Inferring gene regulatory networks

Given $\mathcal{D}^{c}$, we inferred GRN between the TRs and the target genes (i.e. TR-target edges, Figure 1B), using two different state-of-the-art techniques, namely RGBM [42] and ARACNE [37]. The inferred GRNs were weighted and unsigned. For quality control, we remove those TRs whose regulon size were less than 10 in both RGBM and ARACNE inferred GRNs. We used the ‘RGBM’ and ‘corto’ packages in R to perform the RGBM and ARACNE methods for GRN inference, respectively. A brief description of these methods is provided in the Supplementary.

### Scoring TR activities

Given $\mathcal{D}^{c}$ and the GRN ($\mathcal{G}^{c}$) for a particular cancer $c$, the level of activity of a TR in a sample can be estimated as a function of the collective mRNA levels of its targets as illustrated in RGBM [42] and VIPER [8]. More details about TR activity estimation for RGBM are provided in the Supplementary.

### Gene-set enrichment analysis and MR selection

In VIPER, a probabilistic framework that directly integrates the target mode of regulation i.e. whether targets are activated or repressed, confidence in regulator-target interactions and target overlap between different regulators (pleiotropy) is utilized to compute the normalized enrichment score of a TR’s regulon. Since VIPER expresses activity for all the TRs on the same scale i.e. NES, we can now perform differential analysis using a Bayesian statistical framework such as LIMMA [54] (‘limma’ package in R) to identify differentially activated TRs (MRs) between ICR-H and ICR-L samples for a particular cancer $c$.

In FGSEA [53], to identify the differentially active TR regulons between ICR-H and ICR-L primary tumor samples, we first estimate the average mRNA level difference of each gene between the two groups. This difference represents the fold change score (FC-score). To determine the enrichment score with statistical significance for specific TR regulons, we use the ‘fgsea’ function in the ‘fgsea’ package in R [53]. We select TRs with FDR-adjusted [11] $P$$\le $ 0.05 and $|\textrm{NES}^{c}|> 1.0$ for all cancer types as differentially activated MRs (Figure 1E). Figure 1F highlights the activity of the MRs indicating some MRs have high activity in ICR-H samples but low activity in ICR-L samples and vice-versa.

**Fig. 2:**
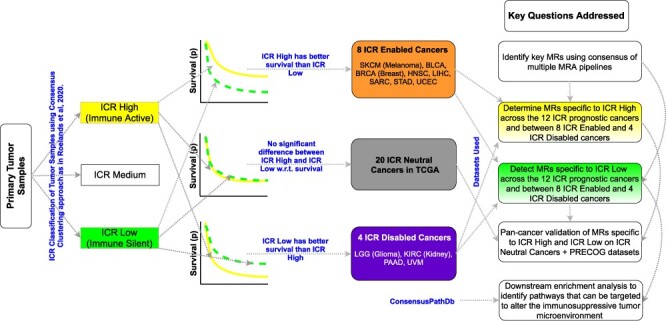
Figure 2 provides insight into the ICR classification, ICR’s prognostic value and the key questions answered in this paper.

In GSVA, a non-parametric, unsupervised technique is used to estimate TR regulon enrichment scores as a function of genes inside and outside the regulons analogously to a competitive gene set test [27]. We use the ‘gsva’ function in the ‘gsva’ package in R providing the expression information, TR regulons, maximum and minimum size of a regulon as input while keeping all other parameters at their default settings. We obtain a sample-specific enrichment score for each TR regulon, which can now be utilized to perform differential analysis using a Bayesian statistical framework, such as LIMMA, to determine the differentially activated TRs (MRs) between ICR-H and ICR-L samples for a cancer type $c$.

### Pathway enrichment analysis

We use ConsensusPathDB [30, 35] for the functional and pathway enrichment analysis of MRs common across the 12 cancer types for the ICR-H and ICR-L phenotypes separately (latest version [36]). ConsensusPathDB allows us to perform overexpression analysis on top of differentially activated MRs to identify significantly enriched molecular functions (M), cellular components (C), biological process (BP), pathways (P) and protein complexes (PC). The advantage of using ConsensusPathDB over a popular tool like DAVID [31] is that it provides the option to search through multiple databases (different types of interactions) to find enriched pathways, unlike DAVID, which only uses the KEGG database. Moreover, unlike ingenuity pathway analysis, ConsensusPathDB is a free open source software available for such enrichment analysis. Since we consider well annotated TFs (genes) along with receptors, kinases and proteins in our list of transcriptional regulators, we only include databases such as Biocarta, CORUM, Innate DB, KEGG, WikiPathways, Reactome, Nepath, PIC and PINdb, all of which are available in ConsensusPathdb, for our downstream enrichment analysis. The visualization of the enriched pathways obtained via ConsensusPathDB is performed using the ‘func2vis’ package in R.

## 3 Experimental results

### MR identification using consensus framework

Detailed information about the $12$ cancers of interest and the number of ICR-H, ICR-L and ICR-M samples in each cancer is provided in Supplementary Table S2. A comparison of the inferred GRNs from the RGBM and ARACNE methods (per cancer $c$) is provided in Supplementary Table S1. In this work, we used four different pipelines for performing MRA: (a) RGBM + FGSEA, (b) RGBM + GSVA, (c) RGBM + VIPER and (d) ARACNE + VIPER and take a consensus i.e. intersection of the MRs determined by these varied pipelines as the differentially activated MRs between ICR-H and ICR-L samples for a particular $c$. For the RGBM + FGSEA method, we used the $|\textrm{NES}^{c}|>1.0$ and FDR-adjusted $P$$\le $ 0.05 as the selection criterion for identifying the differentially activated TRs (MRs). However, for the other 3 pipelines to be less restrictive, we selected all TRs with FDR-adjusted $P$$\le $ 0.05 when comparing the enrichment scores between ICR-H and ICR-L samples as our MRs. Supplementary Figures S1c and S1d illustrate the volcano plot as well as the differential activity of the MRs identified using each of the four different MRA pipelines for an ICR-E cancer (BLCA) and an ICR-D cancer, LGG (brain lower grade glioma), respectively. The total number of consensus MRs for each cancer type of interest is highlighted in the Supplementary. Supplementary Figure S1e highlights the MRs identified using the different MRA pipelines as Venn diagrams for each of the $12$ cancers of interest.

Finally, we investigate whether the MRs identified were influenced by tumor purity levels in the samples. The tumor purity information for each sample was obtained from [58] (available for all the 12 ICR cancers) and [8] (available for a subset of 8 ICR enabled/disabled cancers). We took the tumor purity information as a covariate when performing differential activity analysis using the ‘limma’ package in R and observed that the top differentially active MRs identified without considering tumor purity remained intact for the majority of the 12 cancers of interest, except for cancers with small sample sizes such as SKCM and UCEC (see Supplementary Figure S1f for comparison). This might be the result of (1) imperfect estimation of purity; (2) heterogeneity of the non-tumor-cell compartment (consisting of stroma and different leukocyte subsets with heterogeneous functional activation states, such as T helper 1, T helper 2, M1 and M2 macrophages etc.); (3) the heterogeneity in terms of activation of immune-related pathways, such as IRF1/STAT1 and Wnt $\beta $-catenin signaling in cancer cells [39, 55, 56]; and (4) the dynamic relationship between cancer cells and immune cells, as elucidated by single-cell sequencing studies [7, 10]. Therefore, MRs specific to ICR-H and ICR-L identified by our consensus pipeline might have captured both tumor-immune interplay and cancer-cell intrinsic immunoregulatory signals.

### MR activities across primary tumors for the ICR phenotype

**Fig. 3:**
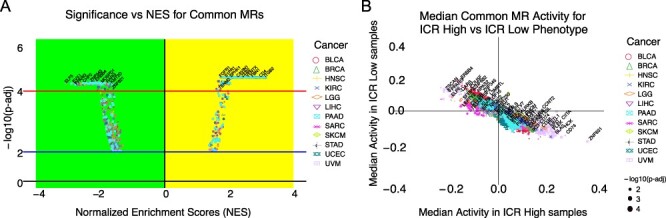
(A) NES for the Consensus MRs obtained via FGSEA were showcased for each $c$ through a volcano plot. For each of these MR, $|\textrm{NES}^{c}$—¿1 and FDR-adjusted $P$$\le $ 0.05 in the RGBM + FGSEA MRA pipeline. (B) Median activities of MRs in ICR-H and ICR-L samples of each cancer were highlighted as scatter points. For these MRs, either median activity was high in ICR-H samples and low in ICR-L samples for a given $c$ (points along the extreme of the diagonal in the $4$th quadrant) or median activity was high in ICR-L samples and low in ICR-H samples (points along the extreme of the diagonal in the $2$nd quadrant).

The goal here was to showcase the activity patterns or the NES of the consensus MRs and illustrate its usage to identify known MRs specific to the ICR-H phenotype. We highlight the NES scores for MRs, as determined by the FGSEA method, for each cancer $c$ as a volcano plot in Figure 3A. We demonstrated the median activity of these MRs (per $c$) across ICR-H and ICR-L samples in Figure 3B. We observed that MRs with NES ¿ 0 tend to have high positive median activity across ICR-H samples and negative median activity across ICR-L samples i.e. points belonging to the $4$th quadrant in Figure 3B (see also Supplementary Figure S3). Thus, these MRs were considered to be specific to the ICR-H phenotype. Similarly, MRs with NES ¡ 0, generally had high positive median activity across ICR-L samples and negative median activity across ICR-H samples i.e. points belonging to the $2$nd quadrant in Figure 3B. Therefore, these MRs were considered to be specific to the ICR-L phenotype.

**Fig. 4:**
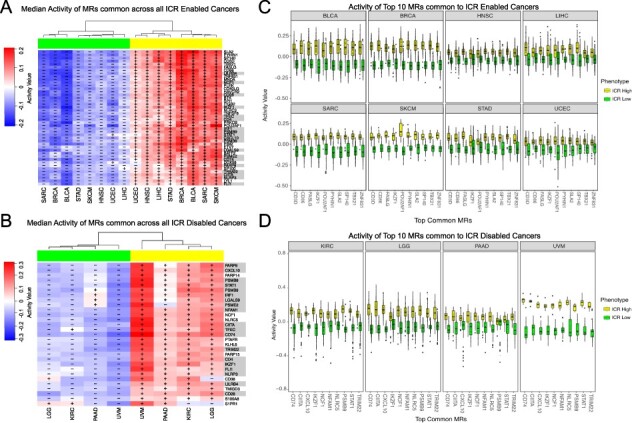
(A) $44$ consensus MRs identified to be differentially active for each of the eight ICR-E cancers. Median activity in ICR-H (‘yellow’) versus ICR-L (‘green’) samples in each cancer, $c$, for these MRs were shown by a heatmap. (B) Median activity in ICR-H versus ICR-L samples in each of the 4 ICR-D cancers was highlighted for the $29$ consensus MRs (ICR-D) i.e MRs that were differentially active in every one of the 4 ICR-D cancers (for each of the 4 MRA pipelines). (C) Box plots comparing MR activities (top 10 MRs based on fold-change in activity, see Supplementary Table S3) in ICR-H versus ICR-L samples for each of the $8$ ICR-E cancers. Predominantly, every one of these MRs had high activity in the ICR-H samples and low activity in the ICR-L samples for each, $c$, and was thus specific to the ICR-H phenotype. (D) Box plots comparing MR activities (top 10 MRs based on fold-change activity, see Supplementary Table S4) in ICR-H vs ICR-L samples for each of the 4 ICR-D cancers. As observed in Figure 4C, all these MRs were specific to the ICR-H phenotype. The $19$ MRs highlighted in ‘gray’ in Figure 4A and B are MRs shared by both the ICR-E and ICR-D cancers.

It is noteworthy that the same MR can appear multiple times (with different color/shapes) in both Figure 3A and B, since we were showcasing the results for all the $12$ cancers together. Additionally, we observed genes such as CD28, CD4, CD74, CIITA, CXCL10, FLI1, IKZF1, IRF1, LGALS9, LILRB4, NCF1, NLRP3, PARP9, PSMB8, PSMB9, PSME2, STAT1, TFEC and TRIM22 were MRs for all the 12 cancer subtypes. Out of the 20 ICR genes, only 6 were in the list of $3674$ TRs (STAT1, IRF1, TBX21, FOXP3 and CXCL10). Remarkably, $3$ of them (STAT1, IRF1 and CXCL10) were MRs consistently positively activated in all the 12 ICR-H cancer samples (see Figure 4A and B). In particular, STAT1, IRF1, TBX21 and CXCL10 were positively activated in ICR-EH tumors and STAT1, IRF1 and CXCL10 in ICR-DH cancer samples. Therefore, this provided a positive validation that our approach could capture expected known genes as MRs for the ICR-H phenotype.

### Consensus MRs across the 12 ICR prognostic cancers

As a first step, we aimed at identifying the most conserved MRs characterizing the two opposite immune phenotypes (ICR-H and ICR-L) within each prognostic cancer category (ICR-E and ICR-D). We then compare the lists of the identified MRs between the eight ICR-E and the four ICR-D cancers. We found $44$ MRs differentially activated between ICR-H and ICR-L phenotypes and common to all the 8 ICR-E cancers as observed in Figure 4A. Similarly, we identified $29$ MRs common to all the $4$ ICR-D cancers as depicted in Figure 4B. Interestingly, we observe each of these MRs has high positive median activity in ICR-H samples and low negative median activity in ICR-L samples and thus is considered to be specific to the ICR-H phenotype. From Figure 4A and B, we determined $19$ MRs (highlighted in ‘gray’ in Figure 4A and B), which were shared across all the 12 cancers of interest (both ICR-E and ICR-D cancers) and were all specific to the ICR-H phenotype.

This observation indicated that (1) the dominant features characterizing the two opposite immune phenotypes (ICR-H and ICR-L) resulted in the upregulation of MRs related to high immune activity rather than MRs capturing immune-exclusion and (2) these MRs are shared independently of the prognostic connotation of immunologic activity (ICR).

### Consensus MRs specific to ICR-H and ICR-L phenotypes

We observed from Figure 4 that all the shared MRs across the $12$ cancers of interest, or even within the ICR-E or ICR-D categories, were specific to the ICR-H phenotype. While this approach led to the identification of dominant features conducive to immune activation (ICR-H specific MRs), we employed a less stringent criterion to identify MRs facilitating immune-exclusion i.e. ICR-L specific MRs.

**Fig. 5:**
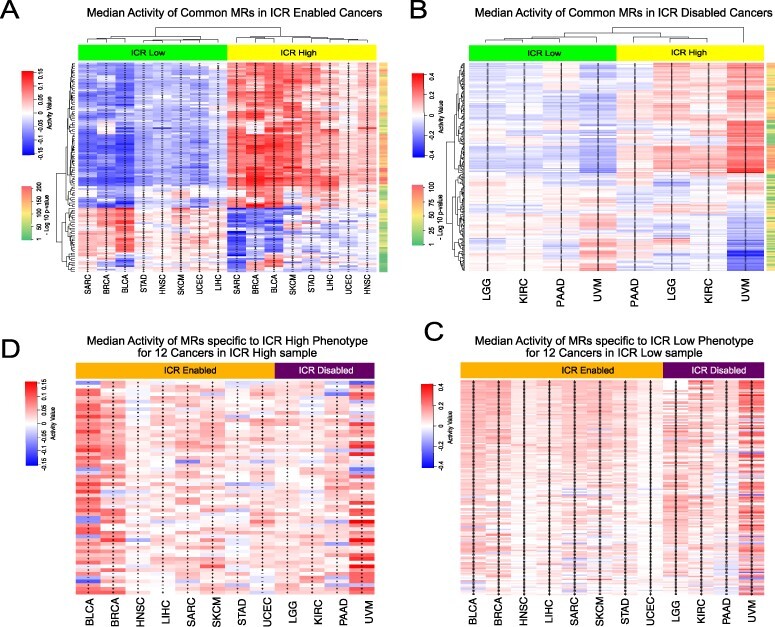
(A) Median activity in ICR-H (‘yellow’) and ICR-L (‘green’) samples for each of the 8 ICR-E cancers were highlighted for consensus MRs ($118$ in total) in $\ge 50\%$ of the cancer subtypes (see Supplementary Table S5). The ‘yellow’ rectangle included MRs which had high + median activity in ICR-H samples and - median activity in ICR-L samples and were specific to the ICR-H phenotype. Similarly, the ‘green’ rectangle highlighted the MRs (32 in total) that were specific to the ICR-L phenotype. (B) Median activity in ICR-H versus ICR-L samples for each of the 4 ICR-D cancers were illustrated for the consensus MRs ($234$ in total) in $\ge 50\%$ of the cancer tissues (see Supplementary Table S7). (C) $155$ MRs specific to the ICR-H phenotype and their median activity in ICR-H samples for the $12$ ICR-E and ICR-D cancers were showcased here (see Supplementary Table S9). (D) $57$ MRs specific to the ICR-L phenotype and their median activity in ICR-L samples for the $12$ ICR-E and ICR-D cancers (see Supplementary Table S10).

Figure 5A highlights consensus MRs which were present in $\ge $ 4 out of the 8 ICR-E cancers ($50\%$ selection criterion). We obtained a set of $118$ such MRs and their corresponding cancer subtypes were elaborated in Supplementary Table S5. Figure 5A illustrated the median activity in the ICR-H and the ICR-L samples for each of the eight ICR-E cancers. A total of $32$ of these MRs had high median activity in ICR-L samples and low median activity in ICR-H samples in at least $50\%$ of the ICR-E cancers (see Supplementary Table S6 for significance). These $32$ MRs were considered to be specific to the ICR-L phenotype for the ICR-E cancers. A similar analysis was performed for ICR-D cancers, as observed in Figure 5B, with the same selection criterion ($50\%$ i.e. 2 out of 4 ICR-D cancers). More details about the set of consensus MRs identified for ICR-D cancers were provided in the Supplementary. A total of $84$ of MRs had high median activity in ICR-L samples and low median activity in ICR-H samples in at least $50\%$ of the ICR-D cancers (see Supplementary Table S8 for significance) and were considered to be specific to the ICR-L phenotype for the ICR-D cancers.

We took a union of the MRs identified to be specific to the ICR-H for the eight ICR-E cancers as well as the four ICR-D cancers and considered only those MRs whose median activity in ICR-H cancer samples was $>0$. This resulted in a total of $155$ MRs (see Supplementary Table S9), which were considered to be specific to ICR-H phenotype across all the $12$ cancers of interest. Figure 5D highlighted the median activity of each of these MRs across all the 12 ICR cancers. Several of these MRs (IRF1, STAT1, CXCL10, TBX21 and FOXP3) were part of the $20$ ICR gene signature whose high expression indicated active immune engagement i.e. the ICR-H phenotype. We performed a similar analysis for the ICR-L phenotype as demonstrated in Figure 5C. This leads to a total of $57$ MRs (see Supplementary Table S10), which were considered to be specific to the ICR-L phenotype across all the $12$ cancers of interest. Figure 5C highlighted the median activity of each of these MRs across all the $12$ ICR cancers. Thus, we identified the set of $155$ MRs and $57$ MRs specific to the ICR-H (immune-active) and ICR-L (immune-silent) phenotype, respectively, and could now perform downstream pathway enrichment analysis to identify molecular mechanisms potentially governing the immune-exclusion functions.

### TGBF1I1 as main negative immune modulator preventing favourable response

**Fig. 6:**
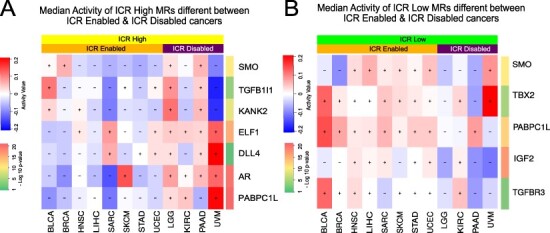
(A) MRs having different median activity patterns in ICR-H samples for ICR-E versus ICR-D cancers (see Supplementary Table S9). (B) MRs having different median activity patterns in ICR-L samples for ICR-E versus ICR-D cancers (see Supplementary Table S10). MRs which were not a TR for a cancer, $c$, were given a median activity of $0$ (e.g. AR does not satisfy the quality control criterion to be a TR for HNSC, KIRC and had 0 median activity and TGFBR3 does not satisfy the quality control criterion to be TR for BRCA, STAD and had 0 median activity).

We observed in Supplementary Table S9, a set of seven MRs with different median activity patterns between the ICR-EH and ICR-DH cancer samples across the eight ICR-E and the four ICR-D cancers of interest. Some of these MRs are not necessarily a TR in each of the $12$ cancers and hence are given a median activity of $0$. Each of these seven MRs had a low negative median activity in a majority of the eight ICR-E cancers and high positive median activity in a majority of the four ICR-D cancers, as depicted in Figure 6A (see Supplementary Table S9 for statistical significance). These $7$ MRs included SMO, TGFB1I1, ELF1, KANK2, DLL4, AR and PABPC1L and could potentially provide insights about the difference in survival prognosis between the ICR-EH and ICR-DH tumor samples. Interestingly, the MR TGF-$\beta $ appears to be positively activated in the ICR-D cancers, whereas it has a median negative activity in majority of the ICR-E cancers. TGF-$\beta $ is a known immune suppressor [66] that inhibits the proliferation of T-cells as well as cytokine production via FOXP3-dependent and independent mechanisms. Thus, its high activation can give some insights for the poor survival prognosis in the case of ICR-H samples belonging to the ICR-D cancers.

Similarly, we observed in Supplementary Table S10, a set of $5$ MRs with different median activity patterns between the ICR-L samples across the 12 ICR cancers. Each of these five MRs had a low negative median activity in a majority of the four ICR-D cancers and high positive median activity in a majority of the eight ICR-E cancers, as depicted in Figure 6B (see Supplementary Table S10 for statistical significance). These $5$ MRs included SMO, TBX2, TGFBR3, PABPC1L and IGF2. The deactivation of these MRs could be associated with better survival prognosis in the ICR-D cancers, whereas their high activity in ICR-E cancers could be associated with poor survival outcomes as shown in [49].

We additionally correlated the activity of MRs with survival outcomes. Interestingly four out of the six MRs with high median activity in ICR-DH (TGFB1I1, KANK2, PABPC1L and SMO) could segregate the entire cohort according to survival outcome ($P$ ¡ 0.05, Bonferroni corrected), all being associated with shorter survival. This effect was coherent across the group of all $12$ ICR cancers and within ICR-E and ICR-D cancer groups (see Supplementary Figure S6C), corroborating their intrinsic immunosuppressive role. Furthermore, ICR prognostication was dependent on the expression of TGFB1I1, as ICR was associated with a favorable outcome only in presence of low TGFB1I1 activity (Bonferroni corrected $P$ = 0.03, see Supplementary Figure S6D and E). Overall, this suggested that TGFB1I1 could be the main immunomodulator and a potential target for immune conversion.

### Enrichment analysis

Once we had identified the MRs that were specific to the ICR-H ($155$ MRs) and ICR-L ($57$) phenotypes across all the 12 cancer subtypes of interest, we performed downstream (enrichment) analysis using ConsensusPathDB [35]. First, we considered all the $155$ MRs specific to the ICR-H phenotype as enriched genes and the background to be the set of all target genes ($23\,216$ genes). We then utilized the overexpression analysis framework of ConsensusPathDB for determining enriched pathways, protein complexes and GO categories. We identified a total of $40$ protein complexes, $826$ GO terms and $237$ pathways that were significantly enriched (FDR-adjusted $P$$\le $ 0.05) for the MRs specific to the ICR-H phenotype. The enriched protein complexes and GO terms specific to ICR-H MRs were detailed in the Supplementary.

The top significantly enriched pathways associated with MRs particular to the ICR-H phenotype involve Immune System (R-HSA168256), Cytokine Signaling in Immune System (R-HSA-1280215), Interferon Signaling (R-HSA-913531), C-type lectin receptor signaling pathway (path:hsa04625), Interleukin-4 and Interleukin-13 signaling (WP4066), etc. as depicted in Supplementary Figure S8A. The MRs that were part of each enriched pathway were illustrated in Supplementary Figure S8B, where the intensity represents the median activity for that MR across all 12 cancer tissues of interest. Interestingly, we observed that the majority of the top significantly enriched pathways are hallmark pathways of immune engagement [9], justifying the ICR-H phenotype, where the high activity of these MRs indicated active immune engagement and at least a partial rejection of the cancer tissue [62].

A similar analysis was performed for the $57$ MRs specific to the ICR-L phenotype. On overexpression analysis, we detected a total of $4$ protein complexes, $131$ GO terms and $30$ pathways to be significantly enriched (FDR-adjusted $P$ ¡ 0.05) for the MRs specific to the ICR-L phenotype (only $33$ of 57 MRs are involved in one or more enriched pathway). The enriched protein complexes included Brg1-associated complex II from CORUM, PDPK1:PIP3:PKC zeta from Reactome, emerin C32 and AF4.com from PINdb as depicted in Supplementary Table S11. The significantly enriched GO terms along with their category level stratification for the ICR-L phenotype were showcased in Supplementary Figure S7B.

**Fig. 7:**
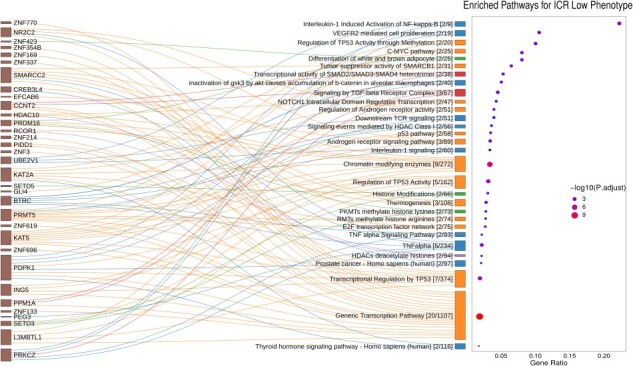
Sankey plot showcasing MRs specific to the ICR-L phenotype (only $33$ out of 57 MRs) were involved in each of the enriched pathways obtained via ConsensusPathDB as a result of overexpression analysis. The dot plot showed the ratio between MRs specific to ICR-L phenotype and the total number of genes in each enriched pathway (FDR-adjusted $P$$\le $ 0.05).

The top significantly enriched pathways particular to the ICR-L phenotype include the Generic Transcription Pathway (R-HSA-212436), Transcriptional Regulation of TP53 (R-HSA-3700989), NOTCH1 Intracellular Domain Regulates Transcription (R-HSA-2122947), TNF-$\alpha $ (WP231) and Interleukin-1 (R-HSA-9020702) signaling pathways, Signaling by TGF-$\beta $ Receptor Complex (R-HSA-170834), etc. We identified five clusters with the predominant clusters belonging to transcriptional regulation and interleukin signaling pathways as depicted in Figure 7. From Figure 7, we observed that the maximum ratio on the $x$-axis reached a value of $\approx $ 0.25, indicating that at max only one-fourth of the genes in a pathway were overexpressed (i.e. MRs specific to the ICR-L phenotype across 12 cancers). Figure 7 showcased that MRs such SMARCC2, KAT2A, KAT5, L3MBTL1, PRMT5 and HDAC10 were the ones which are involved in Regulation of TP53 Activity, NOTCH signaling pathway and generic transcription pathways, whereas MRs such as BTRC, PRKCZ and PDPK1 were the ones associated with Interleukin-1 and TNF-$\alpha $ signaling pathways. Interestingly, MRs such as PRDM16 and ZNF423 lead to enrichment of obesity-related pathways, differentiation of white and brown adipocyte (WP2895) and MRs SETD3 and SETD5 lead to enrichment of Histone modifications (WP2369). Moreover, the MR SALL2 has appeared as a new player in cancer [29] due to its role in the regulation of cell proliferation and survival, its interaction with viral oncogenes and its association with the TP53 tumor suppressor and MYC oncogene, thereby demanding more investigation.

### Validation of MRs for ICR-N cancers and PRECOG datasets

**Fig. 8:**
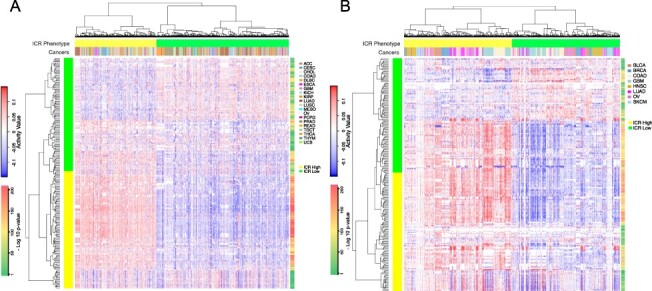
(A) Validation of activity patterns of MRs specific to the ICR-H and ICR-L phenotypes with statistical significance obtained via analysis of the $12$ ICR-E and ICR-D cancers with the 20 ICR-N cancers. The $20$ ICR-N cancers were adrenocortical carcinoma (ACC), cervical squamous cell carcinoma and endocervical adenocarcinoma (CESC), cholangiocarcinoma (CHOL), COAD, lymphoid neoplasm diffuse large B-cell lymphoma (DLBC), esophageal carcinoma (ESCA), GBM, kidney chromophobe (KICH), kidney renal papillary cell carcinoma (KIRP), LUAD, lung squamous cell carcinoma (LUSC), mesothelioma (MESO), OV, pheochromocytoma and paraganglioma (PCPG), prostate adenocarcinoma (PRAD), rectum adenocarcinoma (READ), testicular germ cell tumors (TGCT), thyroid carcinoma (THCA), thymoma (THYM) and uterine carcinosarcoma (UCS). (B) Validation of the activity patterns of MRs specific to the ICR-H and ICR-L phenotypes with statistical significance obtained via analysis of the $12$ ICR-E and ICR-D cancers on eight different datasets in the PRECOG repository.

We performed a validation experiment by comapring the activity patterns of the consensus MRs determined by our framework to be specific to the ICR-H and ICR-L tumor samples in all ICR neutral (ICR-N) cancers. We performed hierarchical clustering of the MRs specific to ICR-L phenotype based on their activity patterns in ICR-N tumor samples. A similar hierarchical clustering was performed for the MRs specific to the ICR-H phenotype and the two dendrograms were assimilated together, as illustrated in Figure 8A. We observed that the MRs, which were specific to the ICR-L phenotype ($55$ out of $57$) had predominantly high activity patterns in all ICR-L samples independent of the type of cancer, whereas they had low activity patterns in the majority of the ICR-H samples for all the $20$ ICR-N cancers in TCGA (see Figure 8A and Supplementary Table S12 for statistical significance). Similarly, for the MRs associated with the ICR-H phenotype, we observed that a majority of these MRs ($145$ out of $155$) had high activities in the ICR-H samples while they had negative activities in the majority of the ICR-L samples as demonstrated in Figure 8A (see Supplementary Table 12 for statistical significance).

Moreover, an additional validation on the set of eight datasets (BLCA, BRCA, COAD, GBM, HNSC, LUAD, OV and SKCM cancers) obtained from the PRECOG repository was conducted. For an MR whose gene expression is not available in a particular dataset, we considered its activity value to be $0$ for the ICR-H and ICR-L samples in that dataset. We again observed that the MRs which were specific to ICR-L phenotype ($53$ out of $57$) had predominantly high activity patterns in all ICR-L samples independent of the type of cancer, whereas they had low activity patterns in a majority of the ICR-H samples in the PRECOG datasets (see Figure 8B and Supplementary Table S13 for statistical significance). Similarly, for the MRs associated with the ICR-H phenotype, we observed that a majority of these MRs ($148$ out of $155$) had high activities in the ICR-H samples while they had negative activities in the majority of the ICR-L samples as demonstrated in Figure 8B (see Supplementary Table 13 for statistical significance). Finally, we highlight in Supplementary Figure 9, the reverse activity patterns of top MRs identified specifically for each cancer subtype, $c$, in the TCGA by the RGBM + FGSEA MRA pipeline (i.e. the MRs per cancer as depicted in Figure 3) in the corresponding PRECOG repository dataset.

These two *in silico* validations confirm that the MRs, which we determined using our consensus framework, were specific to the ICR-L phenotype and should likely be involved in immune exclusion functions. Thus, the enriched pathways associated with these MRs could potentially represent molecular mechanisms driving the immune-excluded cancer phenotype and could be targeted to design therapeutic strategies.

## 4 Discussion

In recent years, TR activities estimated from RNA-Seq data have attracted much attention in cancer research [17, 22, 24, 42]. Although different methodologies [6, 24, 42] have been used to derive TR activity profiles based on different definitions of TR regulons, the common notion was that mRNA levels of the target genes of a TR could be used to determine its activity. Moreover, TRs (MRs) which were differentially activated w.r.t. a phenotype of interest could be treated as prognostic markers and could reveal novel mechanisms associated with the tumor microenvironment. However, the exploration of MRs as therapeutic targets, alone or in combination with other genomic markers is a recent phenomenon only [6, 17, 22, 24, 42].

Here, we designed and applied four different MRA pipelines using the TCGA RNA-Seq data to discover differentially activated TRs (MRs) w.r.t. the immunologic constant of rejection phenotype (ICR-H versus ICR-L). We took a consensus of the MRs identified by these varied MRA pipelines for our goal of identifying key driver MRs for the immune-silent (ICR-L) cancer phenotype. Our network-based framework led to the discovery of $155$ MRs specific to the ICR-H phenotype and $57$ MRs specific to the ICR-L phenotype. Downstream analysis of the MRs specific to ICR-H using ConsensusPathDB showed significant enrichment of protein complexes such as the IRF1 and IRF9 complex with the CXCL10 promoter, DTX3L-PARP9-STAT1 complex, CD4: IL16 complex and pathways that are hallmark pathways of an active immune response.

The primary goal of our work was to identify key driver genes and their associated mechanisms for an immune excluded cancer phenotype (ICR-L). The downstream analysis of MRs specific to ICR-L using ConsensusPathDB resulted in enrichment of the BRG1-associated protein complex, which has a known role in oncogenesis [65]. Moreover, we identified TGF-$\beta $, NOTCH1, Interleukin-1 and TNF-$\alpha $ pathways to be significantly enriched w.r.t. the MRs particular to the ICR-L phenotype. Some of the MRs associated with the ICR-L phenotype lead to significant enrichment of the $\beta $-catenin pathway, whose signaling was known to prevent antitumor immunity in melanoma [56] and other tumors [40] and was associated with an immune-silent phenotype due to lack of CCL4 mediated chemotaxis of effector cells. NOTCH inhibitors are currently in clinical trials and demonstrated clinical activity in heavily pretreated metastatic cancer patients [46].

Similarly, TGF-$\beta $ (TGFB1I1) is a known immune suppressor [66] and its high activation in ICR-D cancers suggests the occurrence of phenomenon, such as immune exhaustion, leading to poor survival rates in these ICR-H tumor samples. This observation is in agreement with very recent data in mice demonstrating that blocking TGFB1 overcomes resistance to immune checkpoint inhibition [18]. The list of MRs generated by our analysis might be exploited for future targeted therapy combinations aimed at converting immune-silent to immune-active tumors, therefore, potentially extending the benefit of immunotherapy.

It is noteworthy that the MRs, PABPC1L and SMO are the only MRs that have high positive median activity in ICR-DH tumors, whereas it has negative median activity in ICR-DL tumors. Moreover, it has negative activity in ICR-EH tumors while having high positive median activity across the majority of the ICR-EL tumors. Thus, PABPC1L and SMO are potential biomarkers that demand further investigation to better understand their prognostic role within the context of ICR.

Briefly, our results demonstrate that TR activity profiles inferred from RNA-Seq data using RGBM + FGSEA, RGBM + GSVA, RGBM + Viper and ARACNE + Viper MRA pipelines can be used to discover key MRs associated with an immune excluded phenotype. *In silico* validation of these consensus MRs was performed in ICR-N cancers and a set of eight different datasets collected from the PRECOG repository, suggesting that these MRs can be used as promising therapeutic markers. Finally, as the data were generated form bulk transcriptome, next steps would include the dissection of the origin of the identified MRs using single cell sequencing techniques and spatial transcriptomics [8], the contribution of somatic mutations and germline variants [52], the validation at protein level [64] and their functional analysis in experimental models.

**Key Messages**

$\bullet $ Network analysis coupled with the availability of large-scale genomic data leads to identification of key driver genes for an immune-silent cancer phenotype.

$\bullet $ Master regulators such as L3MBTL1, SALL2, BTRC, PRKCZ, KAT2A and SMARCC2 are positively active for the immune-silent cancer phenotype in pan-cancer settings.

$\bullet $ The downstream pathway analysis leads to detection of NOTCH1, TGF-$\beta $, Interleukin-1 and TNF-$\alpha $ signaling pathways that were coherently associated with absence of a protective immune response, potentially representing a target for cancer immunologic conversion.

## Supplementary Material

final_diff_bbab168

Reply_to_Reviewers_ICR_v3_bbab168

Supplementary_Information_for_Network_based_Identification_of_Key_MRs_for_ICR_bbab168

## Notes

**Raghvendra Mall** is a scientist at QCRI. His expertise lies in machine learning and computational biology. His research focuses on inferring gene regulatory networks and differential network analysis in complex biological networks.

**Mohamad Saad** is a scientist at QCRI. His area of expertise is statistical genetics and bioinformatics. His research work focuses on GWAS and imputation of missing genotypes in population- and family-based designs.

**Jessica Roelands** is a PhD student affiliated with Sidra Medicine and Leiden University. Her research aims to define immunogenomic drivers of cancer immune responsiveness with a specific focus on colon cancer.

**Darawan Rinchai** is an expert in the fields of immunology and bioinformatics. Her research focuses on defining immune-biomarkers associated with immune checkpoint blockade administration.

**Khalid Kunji** is a software engineer at QCRI with a background in biophysics and mathematics. His work includes protein property prediction, family-based imputation and other work in bioinformatics.

**Hossam Al Meer** is a research associate in Bioinformatics at QCRI with a BSc in Computer Science from UCL and several years of software development experience in industry.

**Wouter Hendrickx** is the PI of the Functional Cancer Omics laboratory at Sidra Medicine. He has extensive experience in cancer research, with emphasis on tumor microenvironment, disease progression and immune phenotypes.

**Francesco M. Marincola**: President and chief scientific officer, Refuge Biotechnologies, Menlo Park, California; editor in chief, *Journal of Translational Medicine*. Previously distinguished research fellow, AbbVie Corporation; chief research officer, Sidra, Qatar; tenured investigator, National Institutes of Health.

**Michele Ceccarelli** is full professor of Computer Science and Engineering at University of Naples ‘Federico II’ where he serves as chair of PhD program in Computational and Quantitative Biology. Michele has made several seminal contributions in cancer genomics and computational systems biology.

**Davide Bedognetti** is director of Cancer Research Department at Sidra Medicine, Doha, Qatar, and associate professor at University of Genova, Italy, and adjunct associate professor at Hamad Bin Khalifa University, Doha, Qatar. His research focuses on defining determinants of cancer immune responsiveness.
