# Supplementary material for: Network-based identification of key master regulators associated with an immune-silent cancer phenotype
Source: Brief Bioinform. 2021 May 13;22(6):bbab168. doi: 10.1093/bib/bbab168 (PMC8574720; doi:10.1093/bib/bbab168)
Supplement: final_diff_bbab168 [file final_diff_bbab168.zip › final_diff_bbab168.pdf]

Briefings in Bioinformatics
doi.10.1093/bioinformatics/xxxxxx
Advance Access Publication Date: 25 March 2021
Problem Solving Protocols

OXFORD

Transcriptome analysis

Network-based Identification of Key Master Regulators associated with an Immune-Silent Cancer Phenotype

Raghvendra Mall [1]*, Mohamad Saad [1], Jessica Roelands [2], Darawan Rinchai [2], Khalid Kunji [1], Hossam Almeer [1], Wouter Hendrickx [2], Francesco M Marincola [3], Michele Ceccarelli [4,5]* and Davide Bedognetti [2,6,7]*

[1] Qatar Computing Research Institute, Hamad Bin Khalifa University, Doha, Qatar, [2] Cancer Research Department, Research Branch, Sidra Medicince, Doha, Qatar, [3] Refuge Biotechnologies, Menlo Park, California, USA, [4] Department of Electrical Engineering and Information Technology (DIETI), University of Naples “Federico II”, Via Claudio 21, 80215 Naples, Italy, [5] Biogem, Istituto di Biologia e Genetica Molecolare, Via Camporeale, Ariano Irpino (AV) and [6] Department of Internal Medicine and Medical Specialities, University of Genova, Genova, Italy [7] College of Health and Life Sciences, Hamad Bin Khalifa University, Doha, Qatar

*To whom correspondence should be addressed.

Associate Editor: XXXXXXX

Received on XXXXX; revised on XXXXX; accepted on XXXXX

Abstract

Motivation: A cancer immune phenotype characterized by an active T-helper 1 (Th1)/cytotoxic response is associated with responsiveness to immunotherapy and favorable prognosis across different tumors. However, in some cancers, such an intratumoral immune activation does not confer protection from progression or relapse. Defining mechanisms associated with immune evasion is imperative to refine stratification algorithms, to guide treatment decisions, and identify candidates for immune-targeted therapy. Molecular alterations governing mechanisms for immune exclusion are still largely unknown. The availability of large genomic datasets offers an opportunity to ascertain key determinants of differential intratumoral immune response.

Results: We follow a network-based protocol to identify transcription regulators (TRs) associated with poor immunologic antitumor activity. We use a consensus of 4 different pipelines consisting of two state-of-the-art gene regulatory network inference techniques, Regularized Gradient Boosting Machines (RGBM) and ARACNE to determine TR regulons, and three separate enrichment techniques, including fast gene-set enrichment analysis (FGSEA), gene set variation analysis (GSVA), and virtual inference of protein-activity by enriched regulon analysis (VIPER) to identify the most important TRs affecting immunologic anti-tumor activity. These TRs, referred to as Master Regulators (MRs), are unique to immune-silent and immune-active tumors respectively. We validated the MRs coherently associated with the immune-silent phenotype across cancers in The Cancer Genome Atlas (TCGA) and a series of additional datasets in the PREdiction of Clinical Outcomes from Genomic Profiles (PRECOG) repository.

Downstream analysis of MRs specific to the immune-silent phenotype resulted in the identification of several enriched candidate pathways, including NOTCH1, TGF-β, Interleukin-1, and TNF-α signaling pathways. TGFB1I1 emerged as one of the main negative immune modulators preventing the favorable effects of a Th1/cytotoxic response.

Availability: Code for this paper is available on GitHub at: `https://github.com/raghvendra5688/ICR_Analysis` and associated data at: `https://data.mendeley.com/datasets/d9ffb7kkzt/3`

Contact: Raghvendra Mall, Michele Ceccarelli and Davide Bedognetti

Supplementary information: Supplementary data is available at *Briefings in Bioinformatics* online.

1 Introduction

Over the last decade, corroborations of the effects of anti-tumor immunity on tumor progression have piled up. The discovery of escape mechanisms of the tumor-immune system, including the identification of immune checkpoints, have led to major advancements in immunotherapy [1, 2, 3]. Despite these advancements, a significant proportion of patients (60-80%) who are treated with immunotherapy still fail to obtain better clinical outcomes [1].

It is now accepted that a T-cell inflamed cancer phenotype is associated with responsiveness to immunotherapy and a favorable prognosis [4, 5, 3, 6]. In this context, we previously defined the Immunologic Constant of Rejection (ICR), a signature that captures the concomitant activation of innate and adaptive immune effector mechanisms required for the occurrence of immune-mediated tissue-specific destruction [7]. The ICR consists of 20 transcripts belonging to four functional categories: CXCR3/CCR5 chemokines (CXCL9, CXCL10, CCL5), Th1/Interferon-γ signaling (IFNG, IL12B, TBX21, CD8A, CD8B, STAT1, IRF1), cytotoxic (GNLY, PRF1, GZMA, GZMB, GZMH), and immune regulatory (CD274, CTLA4, FOXP3, IDO1, PDCD1) functions [7, 8, 9, 10, 11, 12]. We have previously assessed the prognostic and predictive implications of ICR in different settings, and used such ~~signatures~~ a signature to define discrete categories of immune subtypes i.e. *ICR High* (ICR-H), *ICR Medium* (ICR-M) and *ICR Low* (ICR-L) within each solid tumor included in The Cancer Genome Atlas (TCGA) data [3, 13]. However, while prognostic and predictive connotations of the intratumoral immune response have been extensively addressed, the mechanisms governing the molecular alterations underlying immune exclusion are poorly understood. Thus, it is imperative to identify **key driver genes and their associated downstream mechanisms leading to immune exclusion to develop effective therapeutic strategies [12]**.

Using the TCGA database, pan-cancer ~~analysis~~ analyses have sought to address this critical question by correlating the mutational status of driver cancer genes and/or the status of oncogenic signals with the degree of the intratumoral immune response [3, 4, 5, 14, 6, 15]. While some of the oncogenic signals have been validated in experimental models [16, 17], a considerable proportion of intratumoral immune response variation remains unexplained [6]. **It is presently unknown whether an intrinsic activation of transcription regulators (~~TR~~TRs) involved in sustaining the oncogenic process can influence distinct immune disposition and their prognostic implications, and this represents our working hypothesis**.

A necessary condition for tumor progression and drug resistance is transcriptional dysregulation [18, 19]. A majority of cancer driver genes are Transcription Regulators (~~TR~~TRs) [20]. TRs are largely dysregulated due to genomic aberrations or alterations in their regulatory proteins, which in return can modulate the expression of their target genes, referred to as its “regulon”. These TRs have been identified as key oncogenic drivers whose activity patterns are influential to a patientâŁ™s clinical prognosis [21].

Here we use the TCGA RNA-Seq data to discover key driver TRs, referred to as Master Regulators (~~MR~~MRs), for the immune-silent cancer phenotype. We utilize the RNA-Seq data for 12 cancer types, comprising a total of 2,307 primary tumor samples divided into ICR Low (ICR-L and immune-silent) and ICR High (ICR-H and immune-active) [3], each having gene expression for 3,674 TRs and 23,216 target genes. These 12 cancers include 8 tumor types in which ICR bears a favorable prognostic implication referred as ICR Enabled (IRC-E) tumors, and 4 cancer types in which ICR is associated with unfavorable prognosis, namely ICR Disabled (ICR-D) tumors as illustrated in [3].

There have been several methods in the literature [22, 23] that have been used in previous studies to perform ~~master regulator analysis~~ Master Regulator Analysis (MRA). A primary ingredient for MRA is to reverse engineer a high quality ~~gene regulatory network~~ Gene Regulatory Network (GRN) consisting of TR-target gene interactions (regulon or gene sets) from RNA-Seq data. This is one of the central problems in computational biology and a plethora of techniques have been proposed~~for the same~~, including mutual information based method ARACNE [24], and tree-based machine learning techniques such as GENIE [25] and RGBM [26, 27, 28, 29, 30, 31, 32]. In [33], through an open-science competition (DREAM Challenge), the authors compared various GRN inference methods on several synthetic and real datasets. In [26], the authors illustrated the superior performance of RGBM for the DREAM Challenge networks (see Supp. Fig S1b). Hence RGBM is the primary GRN inference technique focused on in this work.

Another key component of MRA is to estimate enrichment/activity scores for ~~TR~~ TRs in a given sample, taking into consideration its regulon. This is essential to identify differentially enriched/activated TRs (MRs). While techniques such as RGBM utilize a simplistic difference in average expression of ~~positive and negative~~ positively and negatively regulated targets to estimate the activity of a TR, methods such as VIPER [34] and MARINA [35], utilize a dedicated algorithm formulated to estimate TR activity taking into account the TR mode of action, the TR-target gene interaction confidence and the pleiotropic nature of each target gene regulation. Moreover, there exists single sample gene set enrichment analysis [36] techniques such as gene set variation analysis (GSVA [37]) and fast gene set enrichment analysis (FGSEA [38]) to estimate enrichment score for each TR in a given sample. **This is utilized for further differential analysis (ICR-H vs ICR-L) to identify the key MRs w.r.t. a phenotype of interest**.

In recent literature, techniques such as Netfactor [39] and [18], take a consensus based approach to identify signature specific MRs or estimate TR activities respectively. It was shown in [18] that since the TR regulons are estimated by taking a consensus approach, they are more robust for downstream tasks with less ~~possibility of being~~ likely to be influenced by false positives. Following the same principle, we identify the MRs specific to immune-active and immune-silent cancer ~~phenotype~~ phenotypes by taking a consensus (intersection) of the MRs determined by using 4 different MRA pipelines: (a) RGBM + FGSEA; (b) RGBM + GSVA; (c) RGBM + VIPER; (d) ARACNE + VIPER. Thus, in our proposed framework, we use two state-of-the-art GRN inference techniques and 3 different gene set enrichment/activity estimation techniques to robustly determine the MRs.

We investigate the MRs that are common across 12 cancer types and are specific to either the ICR-H ~~and~~ or ICR-L ~~phenotypes respectively~~phenotype. We perform a validation of these MRs by observing expected activity patterns (with statistical significance) for ICR-H and ICR-L samples in each of the remaining cancer types in TCGA. ~~These cancer types, referred~~ **These cancer types, referred as ICR Neutral (ICR-N), have no clear correlation between the immunological status and prognosis [3] and thus serve as a test set for pan-cancer validation**. Furthermore, in another replication study, we observe expected activity patterns for MRs specific to either ICR-H ~~and~~ or ICR-L ~~phenotypes respectively~~ phenotype on a set of 8 different datasets (cancer types) in the PREdiction of Clinical Outcomes from Genomics Profiles (PRECOG) repository [40]. Finally, we perform downstream analysis of the MRs specific to ICR-L using ConsensusPathDB [41] to discover corresponding enriched pathways, several of which are potential candidates that can be targeted to readjust the immunosuppressive tumor microenvironment. The primary contributions of our work are:

 The Author 2020. Published by Oxford University Press. All rights reserved. For permissions, please e-mail: journals.permissions@oup.com

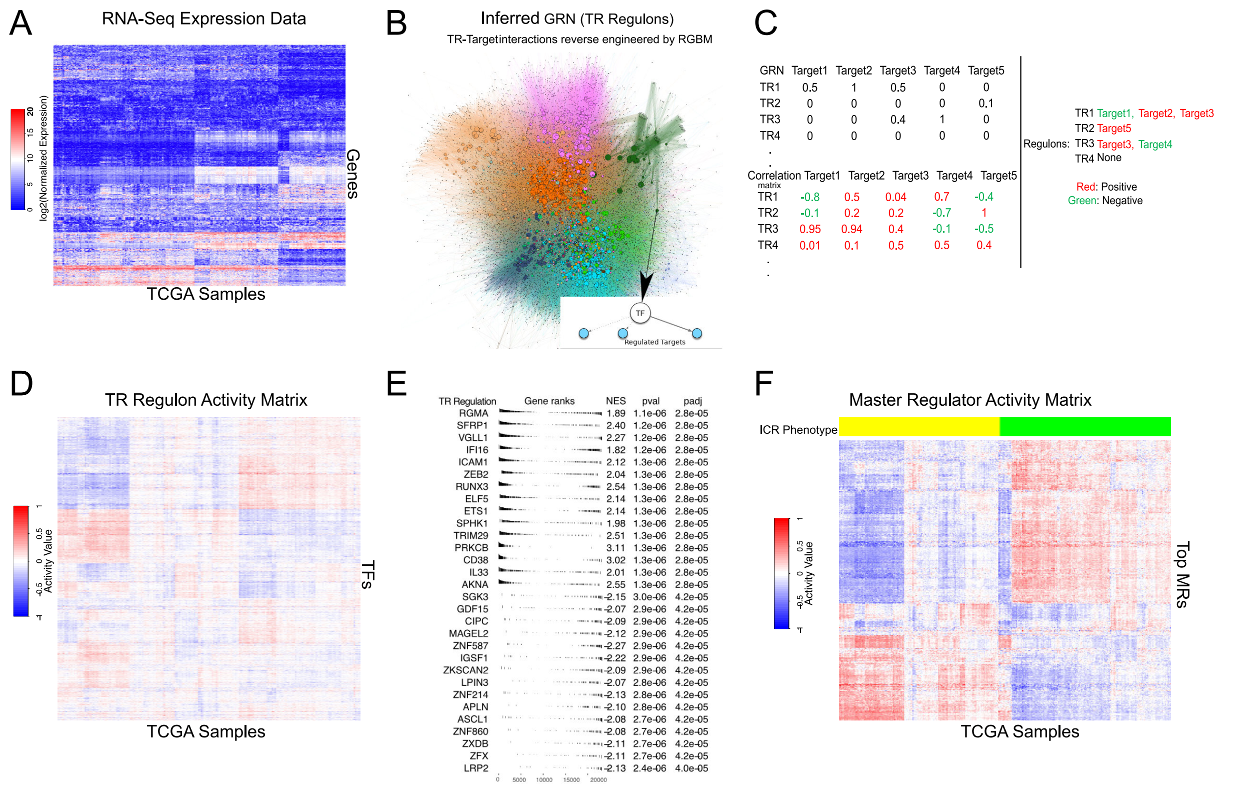

Fig. 1. Identification of differentially active MRs for the ICR phenotype using one of the 4 master regulator analysis pipelines (RGBM + FGSEA). A) RNA-Seq gene expression in the TCGA samples for one cancer, for example, ~~Breast~~ BReast invasive ~~carcinoma~~ CArcinoma (BRCA) was processed to be quantile-normalized and log2 transformed. B) Gene regulatory network (GRN) inferred from the RNASeq data using RGBM for a given cancer~~, c~~. The GRN ~~comprised~~ estimated TR-target interactions (see Supp. Table S1 for details about TR-target network characteristics). C) ~~Determined correlation~~ Correlation matrix determined using RNA-Seq and GRN, leading to the quantification of positively and negatively regulated targets for each TR. D) The single-sample TR activities obtained from gene expression data and the TR regulons. E) Top statistically significant (p-value ≤ 0.05) differentially active TRs, referred to as ~~master regulators~~ Master Regulators (MRs), determined using the ~~Fast Gene Set Enrichment Analysis (~~FGSEA ~~)~~technique. F) Activity matrix corresponding to the MRs for a cancer type along with the encoded phenotype information. Here ‘yellowâĽ™ ~~colored~~ samples ~~referred~~ refer to ICR High phenotype whereas the ‘greenâĽ™ ~~colored~~ samples belong to ICR Low phenotype.

- A framework which takes the consensus of 4 different MRA pipelines to identify MRs specific to immune-active & immune-silent phenotype.
- Validation of the activities of MRs specific to ICR-L and ICR-H on sets ~~of two different datasets~~ from two different data sources i.e. the ICR-N cancer types in TCGA and the PRECOG datasets.
- Downstream analysis of MRs specific to ICR-L lead to the unbiased identification of enriched pathways~~such as~~ , such as the NOTCH1, TGF-β, Interleukin-1 and TNF-α signaling pathways, which can potentially be targeted to readjust the immunosuppressive tumor microenvironment.
- TGFB1I1 emerged as one of the main negative immune modulators preventing the favorable effects of a Th1/cytotoxic response.

Figure 1 illustrates the protocol followed for RGBM + FGSEA (1 ~~out~~ of the 4 consensus methods) to identify the MRs from the RNA-Seq data (see Supp. Fig S1a) differentiating ICR-H from ICR-L phenotype. Table 1 represents the notation table for all the abbreviations used in this work.

2 Materials & Methods

Transcriptional Regulators

We wanted to select a wide list of candidates as TRs, looking for all the genes involved in the process that ~~modulates~~ modulate the frequency, rate or extent of cellular DNA-templated transcription. Therefore, we selected all the genes annotated with the Gene Ontology term GO:0006355 (regulation of transcription) [42]. The interrogation of the Gene Ontology through Ensembl Biomart was performed in August, 2018 resulting in a list of 3,674 TRs. Previously tools such as ARACNE, VIPER, and NetFactor focused on transcription factors (TFs). Moreover, the original RGBM algorithm also exploited an active binding network based on binding sites of TFs. Recently, there ~~had~~ have been studies [43, 44, 45] that extend the hubs of gene regulatory networks to regulatory proteins beyond the TFs. For example, in [43], the authors considered a set of 2,506 regulatory proteins annotated in Gene Ontology (GO) with transcription factor activity and transcription co-factor activity. Our set of 3,674 TRs was a superset of their set including receptors, kinases, growth factors, signal transduction proteins, transcription co-activators and cofactors as candidate regulators.

Other interesting examples where hubs of the networks were focused on signal molecules (and not just TFs), include approach such as SigMaps [44] or surface receptors i.e. the receptors interactome, to identify active ligand-receptors pairs [45]. These studies used ARACNE + VIPER and

TR	Transcription Regulator
MR	Master Regulator
GRN	Gene Regulatory Network
ICR	Immunologic Constant of Rejection
NES	Normalized Enrichment Score
MRA	Mater Regulator Analysis
TCGA	The Cancer Genome Atlas
PRECOG	PREdiction of Clinical Outcomes for Genomics profiles
RGBM	Regularized Gradient Boosting Machine
GSEA	Gene Set Enrichment Analysis
FGSEA	Fast Gene Set-enrichment Analysis
GSVA	Gene Set Variation Analysis
VIPER	Virtual inference of protein activity by enriched regulons
ICR-H	ICR High $\rightarrow$ highest expression of ICR genes $\rightarrow$ immune-active phenotype
ICR-L	ICR Low $\rightarrow$ lowest expression of ICR genes $\rightarrow$ immune-silent phenotype
ICR-M	ICR Medium $\rightarrow$ medium expression of ICR genes
ICR-E	ICR Enabled cancers or 8 cancer types in which ICR-H has favourable prognosis in terms of clinical outcome [3]
ICR-D	ICR Disabled cancers or 4 cancer types in which ICR-L has favourable prognosis in terms of clinical outcome [3]
ICR-N	ICR Neutral cancers (20 cancer types) that have no clear correlation between ICR and prognosis [3]
ICR-EH	ICR Enabled cancer and ICR High sample within that cancer
ICR-EL	ICR Enabled cancer and ICR Low sample within that cancer
ICR-DH	ICR Disabled cancer and ICR High sample within that cancer
ICR-DL	ICR Disabled cancer and ICR Low sample within that cancer

Table 1. List of notations and abbreviations used.

generalized the concept of MRA to generic signal molecules (not just TFs) as originally intended in [24, 35].

Data Acquisition and Normalization

RNA-Seq data from the TCGA website ~~were~~ was downloaded and processed using TCGA biolinks. RNA-Seq data for each cancer was represented as $\mathcal{D}^c = \{g^c_{1,i}, g^c_{2,i}, \ldots, g^c_{p,i}\}$, ~~$\forall i\{1, \ldots, N_c\}$~~ $\forall i \in \{1, \ldots, N_c\}$ where c represents the cancer type, i corresponds to the ith sample, $g^c_{j,i}$ refers to the expression of the jth target gene in sample i and N_c represents the total number of samples available for that c. We had a total of p = $23,216$ target genes, including $3,674$ TRs. The RNA-Seq data from 32 ~~cancers of tissue type primary solid tumors~~ primary solid tumor cancers were used in our analysis. These samples were quantile normalized and log 2 transformed for analysis (see Fig 1A).

From the PRECOG repository, we selected 8 datasets, each corresponding to a different and the largest ~~available dataset~~ unique dataset available for a particular cancer type as the validation set. These included GEO Accession Id: GSE32894 for ~~Bladder Urothelial Carcinoma~~ BLadder urothelial CArcinoma (BLCA), GSE3494 for ~~Breast Invasive Carcinoma~~ BReast invasive CArcinoma (BRCA), GSE39582 for ~~Colon adenocarcinoma~~ COlon ADenocarcinoma (COAD), GSE108474 for ~~Glioblastoma multiforme~~ GlioBlastoma Multiforme (GBM), GSE65858 for Head and Neck ~~squamous cell carcinoma~~ Squamous cell Carcinoma (HNSC), GSE72094 for ~~Lung adenocarcinoma~~ LUng ADenocarcinoma (LUAD), GSE9891 for ~~Ovarian~~ OVarian serous cystadenocarcinoma (OV) and GSE65904 for ~~Skin~~ SKin Cutaneous Melanoma (SKCM). Each of these 8 datasets consisted of 224, 251, 579, 490, 270, 398, 278 and 210 tumor samples respectively and were normalized using ‘rma’ or quantile normalization [46], followed by log 2 transformation, depending on the platform i.e. Affymetrix and Illumina respectively. These normalized datasets along with ICR information for each sample within a cancer type was obtained from [47].

ICR Classification and Cancer Type Selection

Gene signatures found in early studies on tumor rejection by immunotherapy strongly overlap with pathways that were upregulated during other instances of immune-mediated tissue rejection like graft versus host disease, allograft rejection or autoimmunity [48]. This observation led to the formulation of the ICR. More specifically, ICR reflects coordinated activation of IFN-stimulated genes, upregulation of specific chemokine ligands, Th1 polarization, and induction of immune effector functions, paralleled by the counter-activation of immune regulatory mechanisms [8, 10, 3, 12].

~~To classify cancer samples according to their immunologic functional orientation, we~~ We previously classified the cancer samples in the TCGA using ICR classification [3] for each cancer type c. In short, a consensus clustering [3] algorithm based on the expression of the 20 ICR genes [3] was applied ~~on~~ to the cancer samples of a particular cancer c, to classify its samples into three discrete categories: ICR High (ICR-H and immune active ~~or~~ i.e. hot immune phenotype), ICR Medium, and ICR Low (ICR-L and immune silent ~~or~~ i.e. cold immune phenotype).

The cluster with the highest expression of ICR genes was termed ICR-H, while the cluster with the lowest ICR gene expression was termed ICR-L. All samples in the intermediate cluster were defined as ICR Medium (ICR-M) for each cancer, c, as indicated in [3]. The code and instructions to obtain the ICR class labels for all tumor samples of a specific cancer, c, is available in our repository `https://github.com/raghvendra5688/ICR_Analysis` (see Supplementary Table S2 for a breakdown of the tumor samples into the ICR-H, ICR-M and ICR-L categories for each cancer of interest). Our objective was to compare the cancer samples with a highly active immune phenotype i.e. ICR-H with the immune-silent phenotype i.e. ICR-L. In [3], we emphasized that there exists a subset of cancer types where ICR-H has a better survival prognosis than ICR-L. These 8 cancer subtypes were referred to as ICR Enabled (ICR-E) cancers. Similarly, there exists a subset of 4 cancer types for which the ICR-L group has better survival prognosis than ICR-H group, which we defined as ICR Disabled (ICR-D) cancers. All remaining cancers were classified as ICR Neutral (ICR-N). Thus, in this work, **we primarily focused on these 12 cancer types (8 ICR-E and 4 ICR-D cancer types) as the ICR phenotype has prognostic value in these cancer types [3]**.

Inferring Gene Regulatory Networks

Given $\mathcal{D}^c$, we inferred GRN between the TRs and the target genes (i.e. TR-target edges, Fig 1B), using two different state-of-the-art techniques, namely RGBM [26] and ARACNE [24]. The inferred GRNs were ~~unsigned and weighted~~ weighted and unsigned. For quality control, we remove those TRs whose regulon size ~~is~~ were less than 10 in both RGBM and ARACNE inferred GRNs. We used the ‘RGBM’ and ‘corto’ packages in R to ~~implement~~ perform the RGBM and ARACNE methods for GRN inference respectively. A brief description of these methods is provided in the Supplementary.

Scoring TR Activities

Given $\mathcal{D}^c$ and the GRN ($\mathcal{G}^c$) for a particular cancer c, the level of activity of a TR in a sample can be estimated as a function of the collective mRNA levels of its targets as illustrated in RGBM [26] and VIPER [34]. More details about TR activity estimation for RGBM is provided in the Supplementary.

Gene-Set Enrichment Analysis and MR Selection

In VIPER, a probabilistic framework that directly integrates the target mode of regulation i.e. whether targets are activated or repressed, confidence in regulator-target interactions, and target overlap between

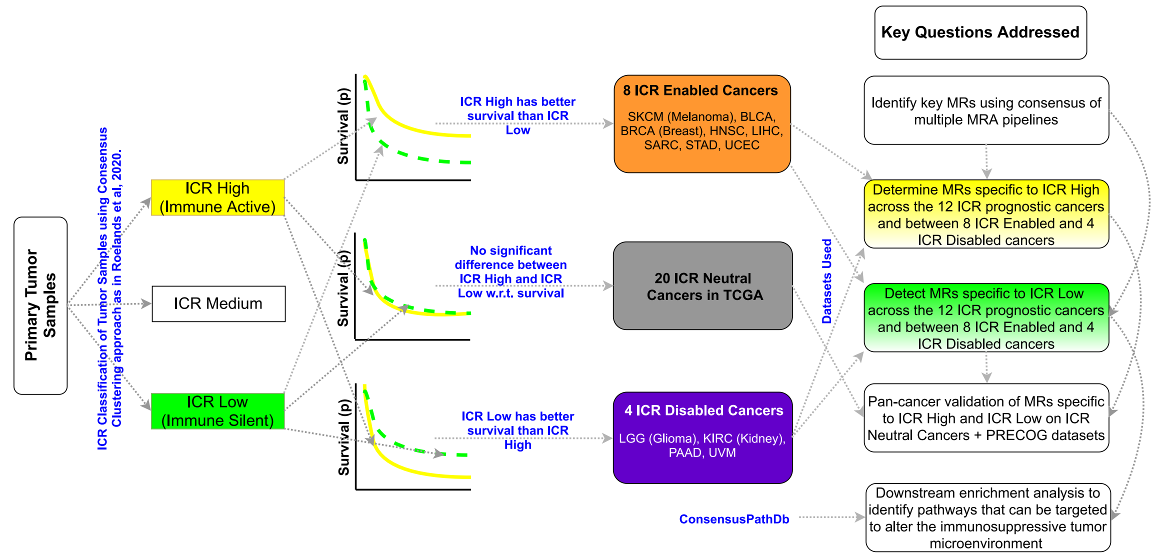

Fig. 2. Figure 2 provides insight ~~to~~ into the ICR classification, ICR's prognostic value and the key questions answered in this paper.

different regulators (pleiotropy) is utilized to compute the normalized enrichment score of a TR's regulon. Since VIPER expresses activity for all the TRs ~~in~~ on the same scale i.e. NES, we can now perform differential analysis using a Bayesian statistical framework such as LIMMA [49] ('limma' package in R) to identify differentially activated TRs (MRs) between ICR-H and ICR-L samples for a particular cancer c.

In FGSEA [38], to identify the differentially active TR regulons between ICR-H and ICR-L primary tumor samples, we first estimate the average mRNA level difference of each gene between the two groups. This difference represents the fold change score (FC-score). To determine the enrichment score with statistical significance for specific TR regulons, we use the 'fgsea' function in the 'fgsea' package in R [38]. We select TRs with FDR-adjusted [50] p-values ≤ 0.05 and $|\text{NES}^c| > 1.0$ for all cancer types as differentially activated MRs (Fig 1E). Figure 1F highlights the activity of the MRs indicating some MRs have high activity in ICR-H samples but low activity in ICR-L samples and vice-versa.

In GSVA, a non-parametric, unsupervised technique is used to estimate TR regulon enrichment scores as a function of genes inside and outside the regulons ~~,~~ analogously to a competitive gene set test [37]. We use the 'gsva' function in the 'gsva' package in R providing the expression information, TR regulons, maximum and minimum size of a regulon as input ~~and~~ while keeping all other parameters ~~to~~ at their default settings. We obtain a sample-specific enrichment score for each TR regulon, which can now be utilized to perform differential analysis using a Bayesian statistical framework, such as LIMMA, to determine the differentially activated TRs (MRs) between ICR-H and ICR-L samples for a cancer type c.

Pathway Enrichment Analysis

We use ConsensusPathDB [41, 51] for the functional and pathway enrichment analysis of MRs common across the 12 cancer types for the ICR-H and ICR-L ~~phenotype~~ phenotypes separately (latest version [52]). ConsensusPathDB allows us to perform over-expression analysis on top of differentially activated MRs to identify significantly enriched molecular functions (M), cellular components (C), biological process (BP)~~and~~ , pathways (P), and protein complexes (PC). The advantage of using ConsensusPathDB over a popular tool like DAVID [53] is that it provides the option to search through multiple databases (different types of interactions) to find enriched pathways, unlike DAVID, which only uses the KEGG database. Moreover, unlike Ingenuity Pathway Analysis, ConsensusPathDB is a free open source software available for such enrichment analysis. Since ~~,~~ we consider well annotated transcription factors (genes) along with receptors, kinases and proteins in our list of transcriptional regulators, we only include databases such as Biocarta, CORUM, Innate DB, KEGG, WikiPathways, Reactome, Nepath, PIC and PINdb, all of which are available in ConsensusPathdb, for our downstream enrichment analysis. The visualization of the enriched pathways obtained via ConsensusPathDB is performed using the 'func2vis' package in R.

3 Experimental Results

MR Identification using Consensus Framework

~~A detailed~~ Detailed information about the 12 cancers of interest and the number of ICR-H, ICR-L and ICR-M samples in each cancer is provided in Supp. Table S2. A comparison of the inferred GRNs ~~of~~ from the RGBM and ARACNE methods (per cancer c) is provided in Supp. Table S1. **In this work, we used four different pipelines for performing MRA: (a) RGBM + FGSEA; (b) RGBM + GSVA; (c) RGBM + VIPER and (d); ARACNE + VIPER and take a consensus i.e. intersection of the MRs determined by these varied pipelines as the differentially activated MRs between ICR-H and ICR-L samples for a particular c.** For the RGBM + FGSEA method, we used the $|\text{NES}^c| > 1.0$ and FDR-adjusted p-value ≤ 0.05 as the selection criterion for identifying the differentially activated TRs (MRs). However, for the other 3 pipelines to be less restrictive, we selected all TRs with FDR-adjusted p-values ≤ 0.05 when comparing the enrichment scores between ICR-H and ICR-L samples as our MRs. Supp. Figures S1c and S1d ~~illustrates~~ illustrate the volcano plot as well as the differential activity of the MRs identified using each of the 4 different MRA pipelines for an ICR-E cancer (BLCA) and an ICR-D cancer, LGG (Brain Lower Grade Glioma), respectively. ~~We obtained a total of 661, 542, 452, 342, 437, 150, 560 and 236~~ The total number of consensus MRs for ~~ICR-E cancers BLCA, BRCA, HNSC, LIHC (Liver hepatocellular carcinoma), SARC (Sarcoma), SKCM, STAD (Stomach adenocarcinoma) and UCEC (Uterine Corpus Endometrial Carcinoma) respectively. Similarly, we obtained a total of 616, 453, 481 and 327 consensus MRs for ICR-D cancers LGG, KIRC (Kidney renal clear cell carcinoma), PAAD (Pancreatic adenocarcinoma) and UVM (Uveal~~

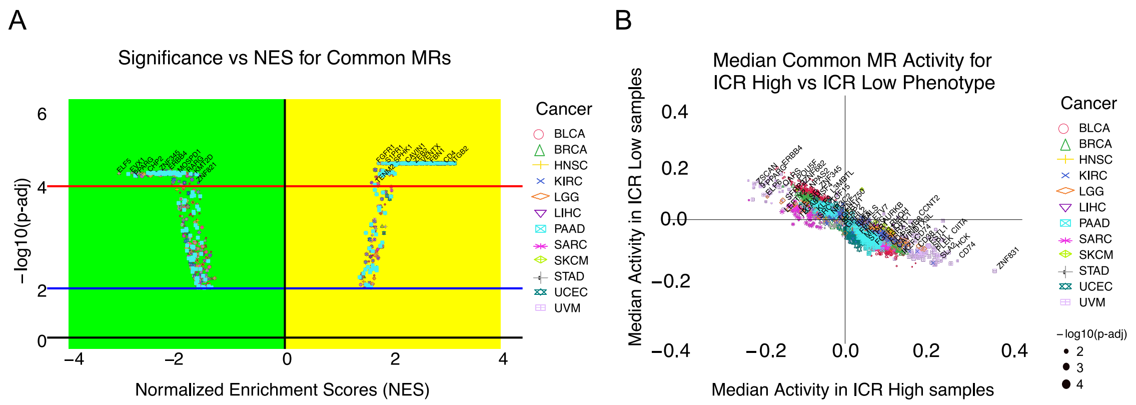

Fig. 3. A) NES for the Consensus MRs obtained via FGSEA were showcased for each c through a volcano plot. For each of these MR, $|NES^c|>1$ and FDR-adjusted p-value ≤ 0.05 in the RGBM + FGSEA MRA pipeline. B) Median activities of MRs in ICR-H and ICR-L samples of each cancer were highlighted as scatter points. For these MRs either median activity was high in ICR-H samples and low in ICR-L samples for a given c (points along the extreme of the diagonal in the 4th quadrant) or median activity was high in ICR-L samples and low in ICR-H samples (points along the extreme of the diagonal in the 2nd quadrant).

~~Melanoma) respectively~~each cancer type of interest is highlighted in the Supplementary. Supp. Figure S1e highlights the MRs identified using the different MRA pipelines as ~~‘Venn ’~~ Venn diagrams for each of the 12 cancers of interest.

Finally, we investigate whether the MRs identified were influenced by tumor purity levels in the samples. The tumor purity information for each sample was obtained from [54] (available for all the 12 ICR cancers) and [55] (available for a subset of 8 ICR Enabled/Disabled cancers). We took the tumor purity information as a covariate when performing differential activity analysis using the ‘limmaâĹ™ package in R and observed that the top differentially active MRs identified without considering tumor purity remained intact for the majority of the 12 cancers of interest, except for ~~cancer~~ cancers with small sample sizes such as SKCM and UCEC (see Supp. Figure S1f for comparison). This might be the result of 1) imperfect estimation of purity; 2) heterogeneity of the non-tumor-cell compartment (consisting of stroma and different ~~leukocytes~~ leukocyte subsets with heterogeneous functional activation states, such as T helper 1, T helper 2, M1 and M2 macrophages etc.); 3) the heterogeneity in terms of activation of immune-related pathways, such is IRF1/STAT1 and Wnt β-catenin signaling in cancer cells [56, 57, 16], and 4) the dynamic relationship between cancer cell and immune cells, as elucidated by single-cell sequencing studies [58, 14]. Therefore, ~~MR~~ MRs specific to ICR-H and ICR-L identified by our consensus pipeline might have captured both tumor-immune interplay and cancer-cell intrinsic immunoregulatory signals.

MR Activities across Primary Tumors for the ICR Phenotype

The goal here was to showcase the activity patterns or the NES of the consensus MRs and illustrate its usage to identify known MRs specific to the ICR-H phenotype. We highlight the NES scores for MRs, as determined by the FGSEA method, for each cancer c as a volcano plot in Fig 3A. We demonstrated the median activity of these MRs (per c) across ICR-H and ICR-L samples in Fig 3B. We observed that MRs ~~whose~~ with NES > 0, tend to have high positive median activity across ICR-H samples and negative median activity across ICR-L samples i.e. points belonging to the 4th quadrant in Fig 3B (see also Supp. Figure S3). Thus, these MRs were considered to be specific to the ICR-H phenotype. Similarly, MRs ~~whose~~ with NES < 0, generally had high positive median activity across ICR-L samples and negative median activity across ICR-H samples i.e. points belonging to the 2nd quadrant in Fig 3B. ~~Thus~~Therefore, these MRs were considered to be specific to the ICR-L phenotype.

It ~~was~~ is noteworthy that the same MR ~~could~~ can appear multiple times (with different color/shapes) in both Figs. 3A and 3B, since we were showcasing the results for all the 12 cancers together. Additionally, we observed genes such as CD28, CD4, CD74, CIITA, CXCL10, FLI1, IKZF1, IRF1, LGALS9, LILRB4, NCF1, NLRP3, PARP9, PSMB8, PSMB9, PSME2, STAT1, TFEC and TRIM22 were MRs for all the 12 cancer subtypes. Out of the 20 ICR genes, only 6 were in the list of 3, 674 TRs (STAT1, IRF1, TBX21, FOXP3, and CXCL10). Remarkably, 3 of them (STAT1, IRF1, and CXCL10) were MRs consistently positively activated in all the 12 ICR-H ~~cancers~~ cancer samples (see Figures 4A and 4B). In particular, STAT1, IRF1, TBX21 and CXCL10 were positively activated in ICR-EH tumors and STAT1, IRF1, and CXCL10 in ICR-DH cancer samples. Therefore, this provided a positive validation that our approach could capture expected known genes as MRs for the ICR-H phenotype.

Consensus MRs across the 12 ICR ~~Prognosticated~~ Prognostic Cancers

~~**The goal was to estimate the MRs for all the 8 ICR-E cancers which were shared with the MRs identified for all the 4 ICR-D cancers.** To verify whether the underlying mechanisms that shape the tumor's immune phenotype were similar across cancer types, we first sought to investigate the consensus MRs within the ICR-E and ICR-D cancer types separately. The presence of shared MRs would then indicate the utilization of an underlying mechanism/process by the tumor microenvironment for each of the 12 cancers belonging to the ICR-E or ICR-D cancer subtypes respectively. The median activity of these MRs across all the samples belonging to ICR-H and ICR-L phenotype for each of the 8~~As a first step, **we aimed at identifying the most conserved MRs characterizing the two opposite immune phenotypes (ICR-H and ICR-L) within each prognostic cancer category (ICR-E and ICR-D).** We then compare

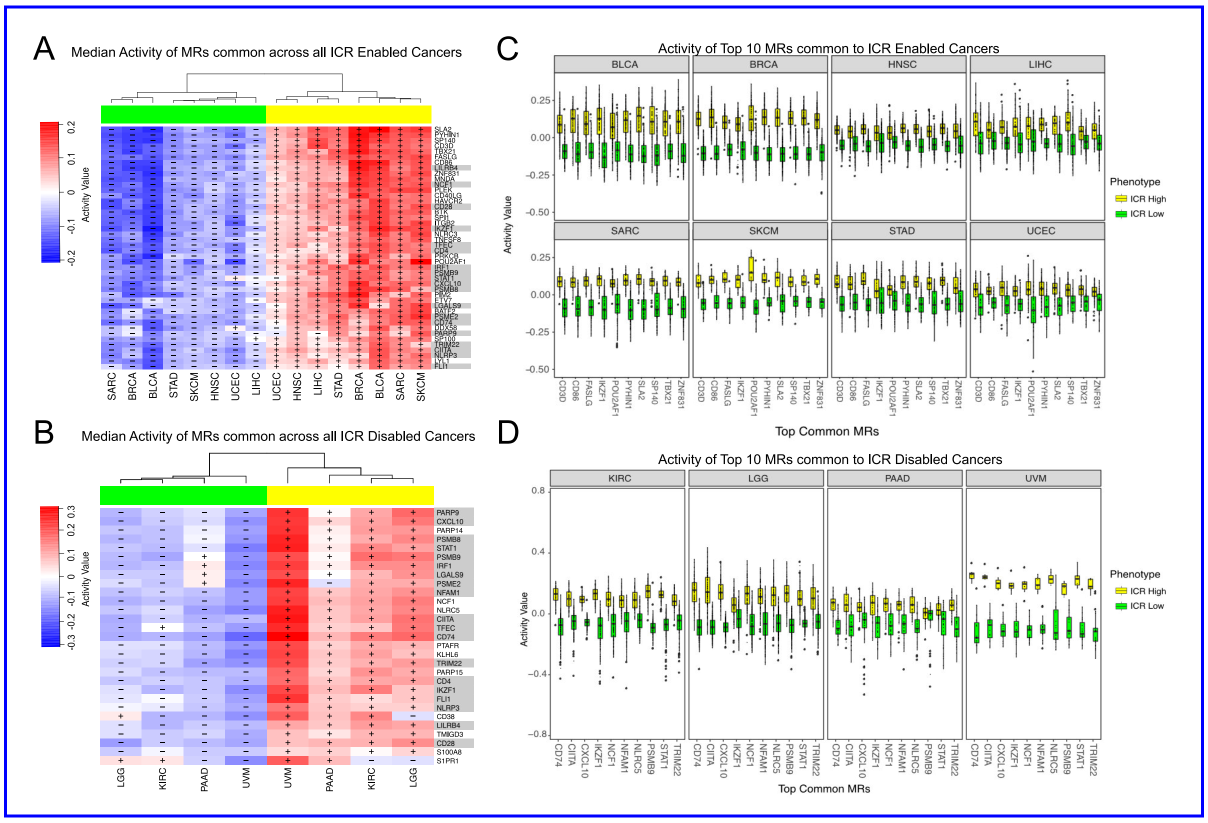

Fig. 4. A) 44 consensus MRs identified to be differentially active for each of the 8 ICR-E cancers. Median activity in ICR-H ('yellow') versus ICR-L ('green') samples in each cancer, *c*, for these MRs were ~~reflected through~~ shown by a heatmap. B) Median activity in ICR-H versus ICR-L samples in each of the 4 ICR-D cancers ~~were~~ was highlighted for the 29 consensus MRs (ICR-D) i.e MRs that were differentially active in every one of the 4 ICR-D cancers (for each of the 4 MRA pipelines). C) Box plots comparing MR activities (top 10 MRs based on fold-change in activity, see Supp. Table S3) in ICR-H versus ICR-L samples for each of the 8 ICR-E cancers. Predominantly, every one of these MRs had high activity in the ICR-H samples and low activity in the ICR-L samples for each, *c*, and was thus specific to the ICR-H phenotype. D) Box plots comparing MR activities (top 10 MRs based on fold-change activity, see Supp. Table S4) in ICR-H vs ICR-L samples for each of the 4 ICR-D cancers. As observed in Fig 4C, all these MRs were specific to the ICR-H phenotype. The 19 MRs highlighted in 'grey' in Figs. 4A and 4B are MRs shared by both the ICR-E and ICR-D cancers.

the lists of the identified MRs between the 8 ICR-E and ~~4~~ the 4 ICR-D cancers~~were depicted in Figures 4A and 4B respectively.~~

~~We next ranked the~~. We found 44 MRs ~~of ICR-E cancers based on the difference in their median activity in the~~ differentially activated between ICR-H ~~versus~~ and ICR-L ~~samples per cancer tissue (see Supp. Table S3) and highlight the activities of the top 10 MRs for~~ phenotypes and common to all the 8 ICR-E cancers as observed in Figure 4~~C. From Figure 4C, we observed that in all of these 8 cancer types,~~ A. Similarly, we identified 29 MRs common to all the 4 ICR-D cancers as depicted in Figure 4B. Interestingly, we observe each of these ~~top 10 MRs had predominantly high activity in the~~ MRs has high positive median activity in ICR-H samples and ~~relatively much lower activity in the~~ low negative median activity in ICR-L samples ~~. Thus, all these MRs were~~ and thus is considered to be specific to the ICR-H phenotype. ~~We performed a similar analysis for the 4~~ From Figures 4A and 4B, we determined 19 MRs (highlighted in 'grey' in Figures 4A and 4B), which were shared across all the 12 cancers of interest (both ICR-E and ICR-D cancers~~by ranking the 29 MRs based on the fold-change in their activities between the~~) and were all specific to the ICR-H phenotype.

This observation indicated that 1) the dominant features characterizing the two opposite immune phenotypes (ICR-H and ICR-L~~samples per cancer type (see Supp. Table S4) and illustrated the activities of the top 10 MRs in Figure 4D. We observed a similar trend as in ICR-E cancers. Hence, these~~) resulted in the upregulation of MRs ~~were also specific to the ICR-H phenotype.~~ ~~**Thus, the 19 MRs (highlighted in 'grey' in Figures 4A and 4B) which were shared across all the 12 cancers of interest (both ICR-E and ICR-D cancers) were all specific to the ICR-H phenotype**~~. related to high immune activity rather than MRs capturing immune-exclusion, and 2) these MRs are shared independently of the prognostic connotation of immunologic activity (ICR).

Consensus MRs Specific to ICR-H and ICR-L Phenotypes

We observed from Fig 4 that all the shared MRs across the 12 cancers of interest, or even within the ICR-E or ICR-D categories, were specific to the ICR-H phenotype. ~~**Our primary aim was to find MRs which were specific**~~ **While this approach led to the** ~~**ICR-L**~~ **identification of dominant features conducive to immune activation (**~~**immune-silent**~~**ICR-H specific MRs)**~~**phenotype across all the 12 cancer subtypes**~~**, we employed a less stringent criterion to identify MRs facilitating immune-exclusion i.e. ICR-L specific MRs.** ~~. To achieve this, we used a less stringent criterion for the selection of MRs.~~

Figure 5A highlights consensus MRs which were present in ≥ 4 out of the 8 ICR-E cancers (50% selection criterion). We obtained a set of 118 such MRs and their corresponding cancer subtypes were elaborated in Supp. Table S5. Figure 5A illustrated the median activity in the ICR-H and the ICR-L samples for each of the 8 ICR-E cancers. 32 of these MRs had high median activity in ICR-L samples and low median activity in ICR-H samples in at least 50% of the ICR-E cancers (see Supp. Table S6 for

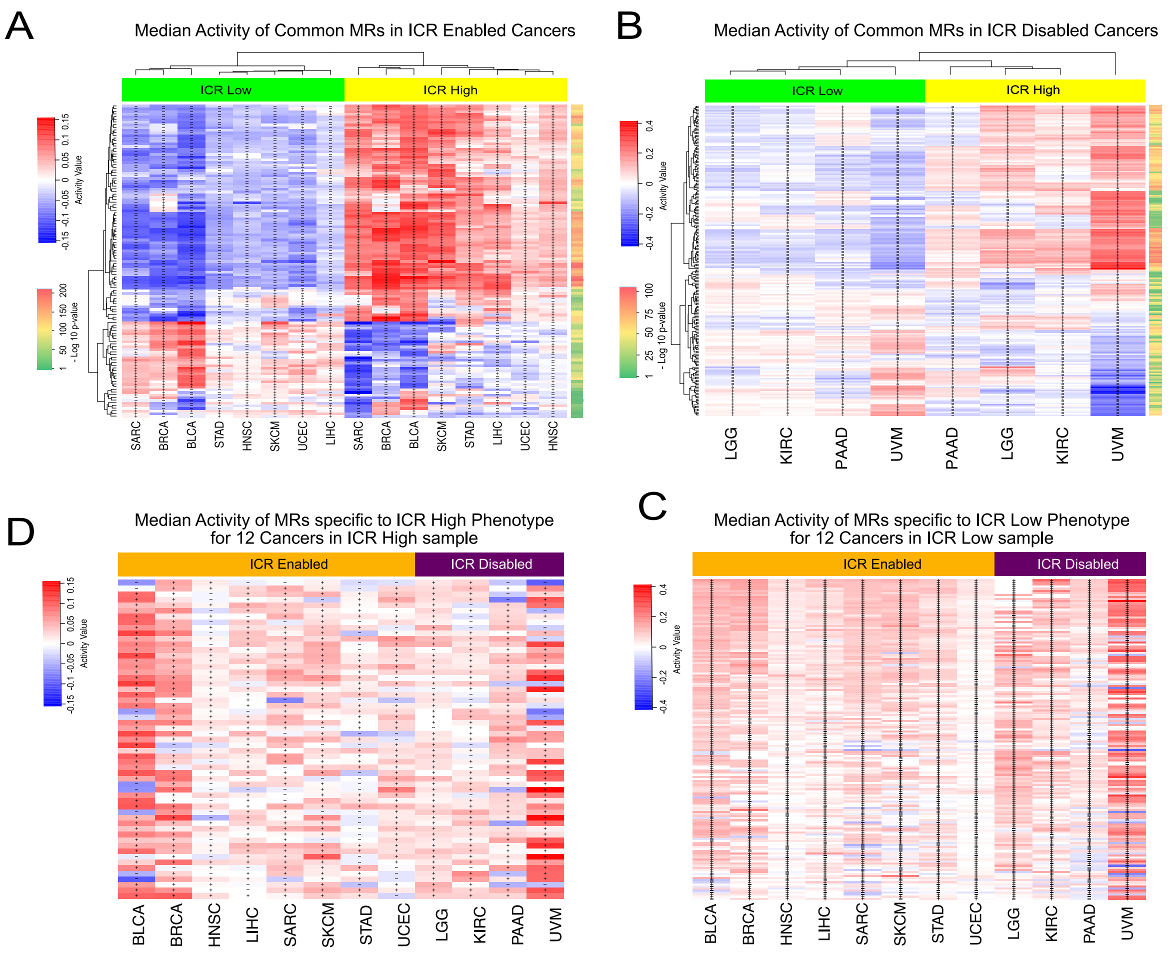

Fig. 5. A) Median activity in ICR-H ('yellow') and ICR-L ('green') samples for each of the 8 ICR-E cancers were highlighted for consensus MRs (118 in total) in $\geq$ 50% of the cancer subtypes (see Supp. Table S5). The 'yellow' rectangle included MRs which had high + median activity in ICR-H samples and - median activity in ICR-L samples and were specific to the ICR-H phenotype. Similarly, the 'green' rectangle highlighted the MRs (32 in total) ~~which~~ that were specific to the ICR-L phenotype. B) Median activity in ICR-H versus ICR-L samples for each of the 4 ICR-D cancers were illustrated for the consensus MRs (234 in total) in $\geq$ 50% of the cancer tissues (see Supp. Table S7). C) 155 MRs specific to the ICR-H phenotype and their median activity in ICR-H samples for the 12 ICR-E and ICR-D cancers were showcased here (see Supp. Table S9). D) 57 MRs specific to the ICR-L phenotype and their median activity in ICR-L samples for the 12 ICR-E and ICR-D cancers (see Supp. Table S10).

significance). These 32 MRs were considered to be specific to the ICR-L phenotype for the ICR-E cancers. A similar analysis was performed for ICR-D cancers, as observed in Figure 5B, with the same selection criterion (50% i.e. 2 out of 4 ICR-D cancers). More details about the set of consensus MRs identified for ICR-D cancers ~~was~~ were provided in the Supplementary. A total of 84 of MRs had high median activity in ICR-L samples and low median activity in ICR-H samples in at least 50% of the ICR-D cancers (see Supp. Table S8 for significance) and were considered to be specific to the ICR-L phenotype for the ICR-D cancers.

We took a union of the MRs identified to be specific to the ICR-H for the 8 ICR-E cancers as well as the 4 ICR-D cancers and considered only those MRs whose median activity in ICR-H cancer samples was > 0. This resulted in a total of 155 MRs (see Supp. Table S9), which were considered to be specific to ICR-H phenotype across all the 12 cancers of interest. Figure 5~~C~~D highlighted the median activity of each of these MRs across all the 12 ICR cancers. **Several of these MRs (IRF1, STAT1, CXCL10, TBX21 and FOXP3) were part of the 20 ICR gene signature whose high expression indicated active immune engagement i.e. the ICR-H phenotype**. We performed a similar analysis for the ICR-L phenotype as demonstrated in Figure 5~~D~~C. This lead to a total of 57 MRs (see Supp. Table S10), which were considered to be specific to the ICR-L phenotype across all the 12 cancers of interest. Figure 5~~D~~C highlighted the median activity of each of these MRs across all the 12 ICR cancers. Thus, we identified the set of 155 MRs and 57 MRs ~~to be specific to~~ specific to the ICR-H (immune-active) and ICR-L (immune-silent) phenotype respectively and could now perform downstream pathway enrichment analysis to identify molecular mechanisms potentially governing the immune-exclusion functions.

TGBF1I1 as Main Negative Immune Modulator Preventing Favourable Response

We observed in Supp. Table S9, a set of 7 MRs with different median activity patterns between the ~~ICR-H~~ICR-EH and ICR-DH cancer samples across the ~~12~~ 8 ICR-E and the 4 ICR-D cancers of interest. Some of these MRs are not necessarily a TR in each of the 12 ~~ICR related~~ cancers and hence are given a median activity of 0. Each of these 7 MRs had a low negative median activity in a majority of the 8 ICR-E cancers and high positive median activity in a majority of the 4 ICR-D cancers, as depicted in Figure 6A (see Supp. Table S9 for statistical significance).

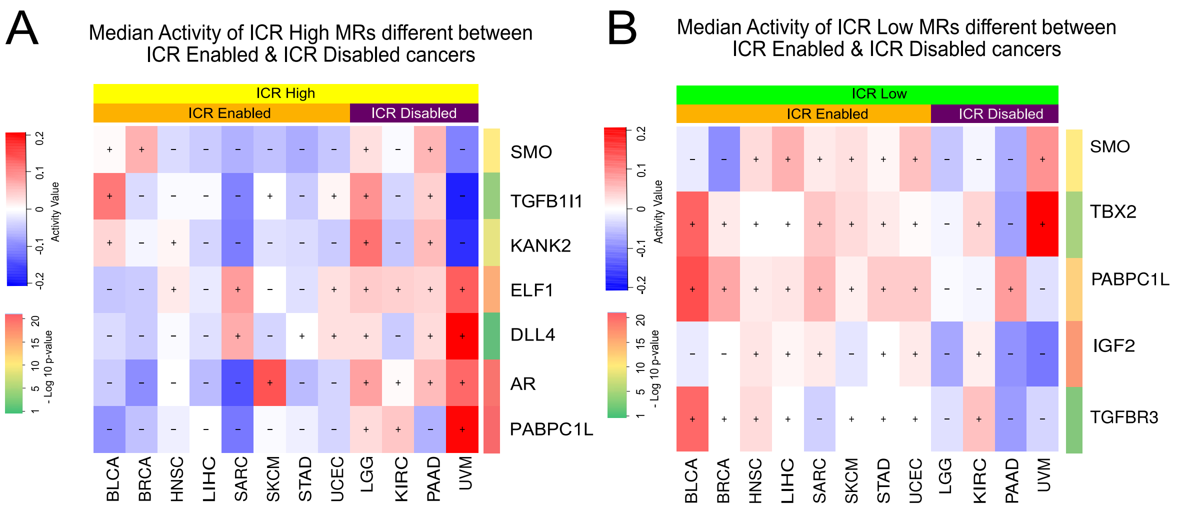

Fig. 6. A) MRs having different median activity patterns in ICR-H samples for ICR-E versus ICR-D cancers (see Supp. Table S9). B) MRs having different median activity patterns in ICR-L samples for ICR-E versus ICR-D cancers (see Supp. Table S10). MRs which were not a TR for a cancer, were given a median activity of 0 (e.g. AR doesn't satisfy the quality control criterion to be a TR for HNSC, KIRC and had 0 median activity and TGFBR3 doesn't satisfy the quality control criterion to be TR for BRCA, STAD and had 0 median activity).

These 7 MRs included SMO, **TGFB1I1**, ELF1, KANK2, DLL4, AR and PABPC1L and could potentially provide insights about the difference in survival prognosis between the ICR-EH and ICR-DH tumor samples. **Interestingly, the MR TGF-β appears to be positively activated in the ICR-D cancers, whereas it has a median negative activity in majority of the ICR-E cancers.** TGF-β is a known immune suppressor [59] that inhibits the proliferation of T-cells as well as cytokine production via FOXP3-dependent and independent mechanisms. Thus, its high activation can give some insights for the poor survival prognosis in the case of ICR-H samples belonging to the ICR-D cancers.

Similarly, we observed in Supp. Table S10, a set of 5 MRs with different median activity patterns between the ICR-L samples across the 12 ICR cancers. Each of these 5 MRs had a low negative median activity in a majority of the 4 ICR-D cancers and high positive median activity in a majority of the 8 ICR-E cancers, as depicted in Figure 6B (see Supp. Table S10 for statistical significance). These 5 MRs included SMO, TBX2, TGFBR3, PABPC1L and IGF2. The deactivation of these MRs could be associated with better survival prognosis in the ICR-D cancers, whereas their high activity in ICR-E cancers could be associated with poor survival outcomes as shown in [3].

We additionally correlated the activity of MRs with survival outcomes. Interestingly 4 out of the 6 MRs with high median activity in ICR-DH (**TGFB1I1**, KANK2, PABPC1L and SMO) could segregate the entire cohort according to survival outcome (p-value < 0.05, Bonferroni corrected), all being associated with shorter survival. This effect was coherent across the group of all 12 ICR cancers and within ICR-E and ICR-D cancer groups (see Supp. Fig S6C), corroborating their intrinsic immuno-suppressive role. Furthermore, ICR prognostication was dependent on the expression of TGFB1I1, as ICR was associated with a favourable outcome only in presence of low ~~activity of~~ TGFB1I1 activity (Bonferroni corrected p-value = 0.03, see Supp. Figs S6D and S6E). Overall, this suggested that TGFB1I1 could be the main immunomodulator and a potential target for immune conversion.

Enrichment Analysis

Once we had identified the MRs ~~which~~ that were specific to the ICR-H (155 MRs) and ICR-L (57) ~~phenotype respectively~~ phenotypes across all the 12 cancer subtypes of interest, we performed downstream (enrichment) analysis using ConsensusPathDB [41]. ~~Firstly~~First, we considered all the 155 MRs specific to the ICR-H phenotype as enriched genes and the background to be the set of all target genes (23,216 genes). We then utilized the over-expression analysis framework of ConsensusPathDB for determining enriched pathways, protein complexes, and gene ontology (GO) categories. We identified a total of 40 protein complexes, 826 GO terms, and 237 pathways that were significantly enriched (FDR-adjusted p-value ≤ 0.05) for the MRs specific to the ICR-H phenotype. The enriched protein complexes and GO terms specific to ICR-H MRs were detailed in the Supplementary.

The top significantly enriched pathways associated with MRs particular to the ICR-H phenotype involve Immune System (R-HSA168256), Cytokine Signaling in Immune System (R-HSA-1280215), Interferon Signaling (R-HSA-913531), C-type lectin receptor signaling pathway (path:hsa04625), Interleukin-4 and Interleukin-13 signaling (WP4066), etc. as depicted in Supp. Fig S8A. The MRs ~~which~~ that were part of each enriched pathway were illustrated in Supp. Fig S8B, where the intensity represents the median activity for that MR across all ~~the~~ 12 cancer tissues of interest. **Interestingly, we observed that the majority of the top significantly enriched pathways are hallmark pathways of immune engagement [7], justifying the ICR-H phenotype, where the high activity of these MRs indicated active immune engagement and at least a partial rejection of the cancer tissue [9].**

A similar analysis was performed for the 57 MRs specific to the ICR-L phenotype. On over-expression analysis, we detected a total of 4 protein complexes, 131 GO terms, and 30 pathways to be significantly enriched (FDR-adjusted p-value < 0.05) for the MRs specific to the ICR-L phenotype (only 33 ~~out~~ of 57 MRs are involved in one or more enriched pathway). The enriched protein complexes included Brg1-associated complex II from CORUM, PDPK1:PIP3:PKC zeta from Reactome, emerin C32 and AF4.com from PINdb as depicted in Supp. Table S11.

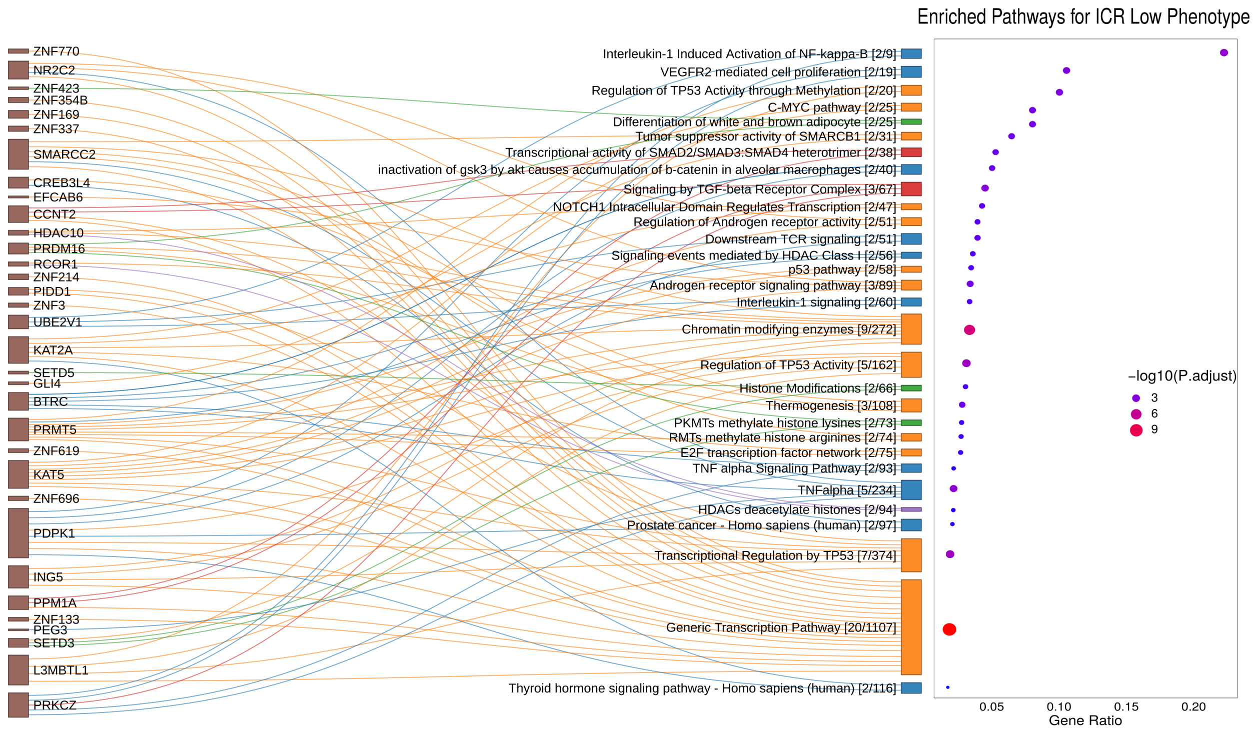

Fig. 7. Sankey plot showcasing MRs specific to the ICR-L phenotype (only 33 out of 57 MRs) were involved in each of the enriched pathways obtained via ConsensusPathDB as a result of overexpression analysis. The dot plot showed the ratio between MRs specific to ICR-L phenotype and the total number of genes in each enriched pathway (FDR-adjusted p $\leq$ 0.05).

The significantly enriched GO terms along with their category level stratification for the ICR-L phenotype ~~was~~ were showcased in Supp. Fig S7B.

The top significantly enriched pathways particular to the ICR-L phenotype include the Generic Transcription Pathway (R-HSA-212436), Transcriptional Regulation of TP53 (R-HSA-3700989), NOTCH1 Intracellular Domain Regulates Transcription (R-HSA-2122947), TNF-α (WP231) and Interleukin-1 (R-HSA-9020702) signaling pathways, Signaling by TGF-β Receptor Complex (R-HSA-170834) etc. We identified 5 clusters with the predominant clusters belonging to transcriptional regulation and interleukin signaling pathways as depicted in Figure 7. From Figure 7, we observed that the maximum ratio on the x-axis reached a value of $\approx$ 0.25, indicating that at max only one-fourth of the genes in a pathway were overexpressed (i.e. MRs specific to the ICR-L phenotype across 12 cancers). Figure 7 showcased that MRs such SMARCC2, KAT2A, KAT5, L3MBTL1, PRMT5 and HDAC10 were the ones which are involved in Regulation of TP53 Activity, NOTCH signaling pathway and generic transcription pathways, whereas MRs such as BTRC, PRKCZ, PDPK1 were the ones associated with Interleukin-1 and TNF-α signaling pathways. Interestingly, MRs such as PRDM16 and ZNF423 lead to enrichment of obesity-related pathways, differentiation of white and brown adipocyte (WP2895) and MRs SETD3 and SETD5 lead to enrichment of Histone modifications (WP2369). Moreover, the MR SALL2 has appeared as a new player in cancer [60] due to its role in the regulation of cell proliferation and survival, its interaction with viral oncogenes, and its association with the TP53 tumor suppressor and MYC oncogene, thereby ~~,~~ demanding more investigation.

Validation of MRs for ICR-N cancers & PRECOG datasets

We performed a validation experiment by comapring the activity patterns of the consensus MRs determined by our framework to be specific to the ICR-H and ICR-L tumor samples in all ICR neutral (ICR-N) cancers. We performed hierarchical clustering of the MRs specific to ICR-L phenotype based on their activity patterns in ICR-N tumor samples. A similar hierarchical clustering was performed for the MRs specific to the ICR-H phenotype and the two dendrograms were assimilated together, as illustrated in Figure 8A. We observed that the MRs, which were specific to the ICR-L phenotype (55 out of 57) had predominantly high activity patterns in all ICR-L samples independent of the type of cancer, whereas they had low activity patterns in the majority of the ICR-H samples for all the 20 ICR-N cancers in TCGA (see Figure 8A and Supp. Table S12 for statistical significance). Similarly, for the MRs associated with the ICR-H phenotype, we observed that a majority of these MRs (145 out of 155) had high activities in the ICR-H samples while they had negative activities in the majority of the ICR-L samples as demonstrated in Figure 8A (see Supp. Table 12 for statistical significance).

Moreover, an additional validation on the set of 8 datasets (BLCA, BRCA, COAD, GBM, HNSC, LUAD, OV and SKCM cancers) obtained from the PRECOG repository was conducted. For ~~an~~ a MR whose gene expression is not available in a particular dataset, we considered its activity value to be 0 for the ICR-H and ICR-L samples in that dataset. We again observed that the MRs which were specific to ICR-L phenotype (53 out of 57) had predominantly high activity patterns in all ICR-L samples independent of the type of cancer, whereas they had low activity patterns in ~~the~~ a majority of the ICR-H samples in the PRECOG datasets (see Figure 8B and Supp. Table S13 for statistical significance). Similarly, for the MRs associated with the ICR-H phenotype, we observed that a majority of these MRs (148 out of 155) had high activities in the ICR-H samples while they had negative activities in the majority of the ICR-L samples as demonstrated in Figure 8B (see Supp. Table 13 for statistical significance). Finally, we highlight in Supp. Fig. 9, the reverse activity patterns of top

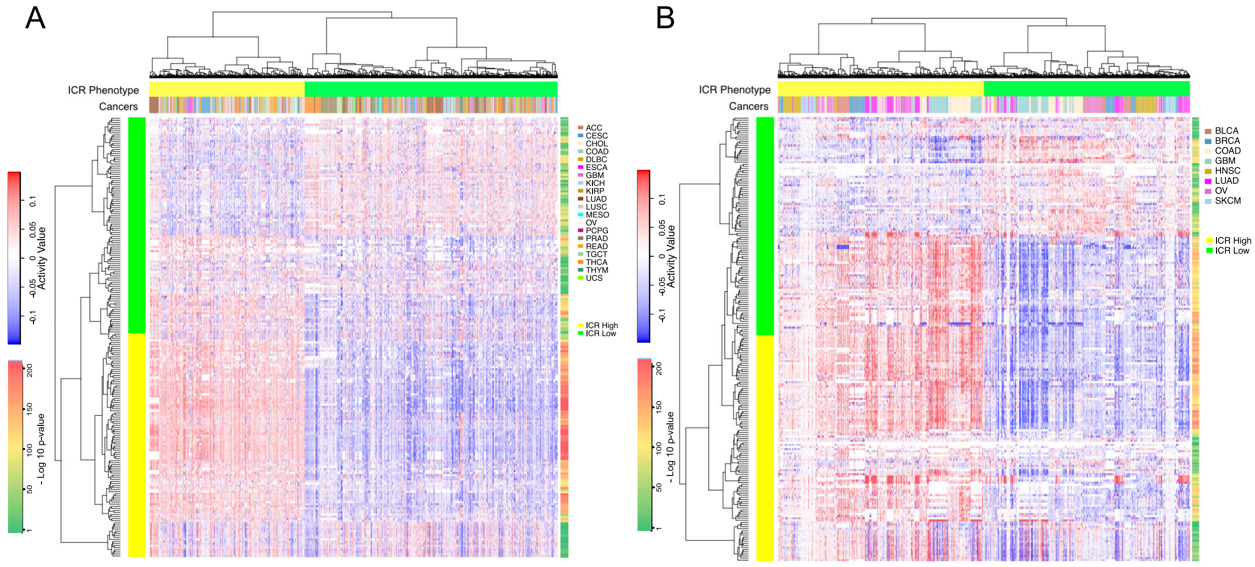

Fig. 8. A) Validation of activity patterns of MRs specific to the ICR-H and ICR-L ~~phenotype~~ phenotypes with statistical significance obtained via analysis of the 12 ICR-E and ICR-D cancers ~~on~~ with the 20 ICR-N cancers. The 20 ICR-N cancers were Adrenocortical carcinoma (ACC), Cervical squamous cell carcinoma and endocervical adenocarcinoma (CESC), Cholangiocarcinoma (CHOL), COAD, Lymphoid Neoplasm Diffuse Large B-cell Lymphoma (DLBC), Esophageal carcinoma (ESCA), GBM, Kidney Chromophobe (KICH), Kidney renal papillary cell carcinoma (KIRP), LUAD, Lung squamous cell carcinoma (LUSC), Mesothelioma (MESO), OV, Pheochromocytoma and Paraganglioma (PCPG), Prostate adenocarcinoma (PRAD), Rectum adenocarcinoma (READ), Testicular Germ Cell Tumors (TGCT), Thyroid carcinoma (THCA), Thymoma (THYM) and Uterine Carcinosarcoma (UCS). B) Validation of the activity patterns of MRs specific to the ICR-H and ICR-L ~~phenotype~~ phenotypes with statistical significance obtained via analysis of the 12 ICR-E and ICR-D cancers on 8 different datasets in the PRECOG repository.

MRs identified specifically for each cancer subtype, c, in the TCGA by the RGBM + FGSEA MRA pipeline (i.e. the MRs per cancer as depicted in Figure 3) in the corresponding PRECOG repository dataset.

These two in-silico validations confirm that the MRs, which we determined using our consensus framework, were specific to the ICR-L phenotype and should likely be involved in immune exclusion functions. Thus, the enriched pathways associated with these MRs could potentially represent molecular mechanisms driving the immune-excluded cancer phenotype and could be targeted to design therapeutic strategies.

4 Discussion

In recent years TR activities estimated from RNA-Seq data have attracted much attention in cancer research [18, 26, 30, 31]. Although different methodologies [18, 26, 35] ~~had~~ have been used to derive TR activity profiles based on different definitions of TR regulons, the common notion was that mRNA levels of the target genes of a TR could be used to determine its activity. Moreover, TRs (MRs) which were differentially activated w.r.t. a phenotype of interest could be treated as prognostic markers and could ~~as well~~ reveal novel mechanisms associated with the tumor microenvironment. However, the exploration of MRs as therapeutic targets, alone or in combination with other genomic markers ~~was~~ is a recent phenomenon only [18, 26, 30, 31, 35].

Here, we designed and applied 4 different MRA pipelines using the TCGA RNA-Seq data to discover differentially activated TRs (MRs) w.r.t. the Immunologic Constant of Rejection phenotype (ICR-H vs ICR-L). We took a consensus of the MRs identified by these varied MRA pipelines for our goal of identifying key driver MRs for the immune-silent (ICR-L) cancer phenotype. Our network-based framework led to the discovery of 155 MRs specific to the ICR-H phenotype and 57 MRs specific to the ICR-L phenotype. Downstream analysis of the MRs specific to ICR-H using ConsensusPathDB showed significant enrichment of protein complexes such as the IRF1 and IRF9 complex with the CXCL10 promoter, DTX3L-PARP9-STAT1 complex, CD4: IL16 complex and pathways that ~~were~~ are hallmark pathways of an active immune response.

~~Since, the~~ The primary goal of our work was to identify key driver genes and their associated mechanisms for an immune excluded cancer phenotype (ICR-L)~~, downstream~~ . Downstream analysis of MRs specific to ICR-L using ConsensusPathDB resulted in enrichment of the BRG1-associated protein complex which has a known role in oncogenesis [61]. Moreover, we identified TGF-β, NOTCH1, Interleukin-1 and TNF-α pathways to be significantly enriched w.r.t. the MRs particular to the ICR-L phenotype. Some of the MRs associated with the ICR-L phenotype lead to significant enrichment of the β-catenin pathway, whose signaling was known to prevent antitumor immunity in melanoma [16] and other tumors [62] and was associated with an immune-silent phenotype ~~,~~ due to lack of CCL4 mediated chemotaxis of effector cells. NOTCH inhibitors are currently in clinical trials and demonstrated clinical activity in heavily pretreated metastatic cancer patients [63].

Similarly, TGF-β (TGFB1I1) is a known immune suppressor [59] and ~~it's~~ its high activation in ICR-D cancers suggests the occurence of phenomenon, such as immune exhaustion, leading to poor survival rates in these ICR-H tumor samples. This observation is in agreement with very recent data in mice demonstrating that blocking TGFB1 overcomes resistance to immune checkpoint inhibition [64]. The list of MRs generated by our analysis might be exploited for future targeted therapy combinations aimed at converting immune-silent to immune-active tumors, therefore, potentially extending the benefit of immunotherapy.

It is noteworthy that the ~~MR~~MRs, PABPC1L and SMO, are the only MRs that have high positive median activity in ICR-DH tumors, whereas it has negative median activity in ICR-DL tumors. Moreover, it has negative activity in ICR-EH tumors while having high positive median activity across the majority of the ICR-EL tumors. Thus, PABPC1L and SMO are

potential biomarkers that demand further investigation to better understand their prognostic role within the context of ICR.

Briefly, our results demonstrate that TR activity profiles inferred from RNA-Seq data using RGBM + FGSEA, RGBM + GSVA, RGBM + Viper and ARACNE + Viper MRA pipelines can be used to discover key MRs associated with an immune excluded phenotype. In silico validation of these consensus MRs ~~was~~ were performed in ICR-N cancers and a set of 8 different datasets collected from the PRECOG repository, suggesting that these MRs can be used as promising therapeutic markers. Finally, as ~~data were~~ the data was generated form bulk transcriptome, next steps would include the dissection of the origin of the identified MRs using single cell sequencing techniques and spatial ~~transcriptomic~~ transcriptomics [6], the contribution of somatic mutations and germline variants [65], the validation at protein level [66], and their functional analysis in experimental models.

Key Messages

- Network analysis coupled with the availability of large-scale genomic data leads to identification of key driver genes for an immune-silent cancer phenotype.
- Master Regulators such as L3MBTL1, SALL2, BTRC, PRKCZ, KAT2A and SMARCC2 are positively active for the immune-silent cancer phenotype in pan-cancer settings.
- Downstream pathway analysis leads to detection of NOTCH1, TGF-β, Interleukin-1 and TNF-α signaling pathways that were coherently associated with absence of a protective immune response, potentially representing a target for cancer immunologic conversion.

Biographical Note

- **Raghvendra Mall** is Scientist at QCRI. His expertise lies in Machine Learning and Computational Biology. His research focuses on inferring gene regulatory networks and differential network analysis in complex biological networks.
- **Mohamad Saad** is a Scientist at QCRI. His area of expertise is Statistical Genetics and Bioinformatics. His research work focuses on GWAS and imputation of missing genotypes in population- and family-based designs.
- **Jessica Roelands** is a PhD-student affiliated with Sidra Medicine and Leiden University. Her research aims to define immunogenomic drivers of cancer immune responsiveness with a specific focus on colon cancer.
- **Darawan Rinchai** is an expert in the fields of immunology and bioinformatics. Her research focuses on defining immune-biomarkers associated with immune checkpoint blockade administration.
- **Khalid Kunji** is a software engineer at QCRI with a background in biophysics and mathematics. His work includes protein property prediction, family-based imputation, and other work in bioinformatics.
- **Hossam Al Meer** is a Research Associate in Bioinformatics at QCRI with a BSc in Computer Science from UCL and several years of software development experience in industry.
- **Wouter Hendrickx** is the PI of the Functional Cancer Omics lab at Sidra Medicine. He has extensive experience in cancer research, with emphasis on tumor microenvironment, disease progression and immune phenotypes.
- **Francesco M. Marincola**: President and Chief-Scientific-Officer, Refuge Biotechnologies, Menlo Park, California, Editor-in-Chief, Journal of Translational Medicine. Previously Distinguished-Research-Fellow, AbbVie Corporation; Chief-Research-Officer, Sidra, Qatar; Tenured Investigator, National Institutes of Health.
- **Michele Ceccarelli** is full professor of Computer Science and Engineering at University of Naples "Federico II" where he serves as chair of PhD program in Computational and Quantitative Biology. Michele has made several seminal contributions in Cancer Genomics and Computational Systems Biology.
- **Davide Bedognetti** is Director of Cancer Research Department at Sidra Medicine, Doha, Qatar and Associate Professor at University of Genova, Italy and Adjunct Associate Professor at Hamad Bin Khalifa University, Doha, Qatar. His research focuses on defining determinants of cancer immune responsiveness.

Funding and Acknowledgement

This research has been supported by Qatar Foundation, Qatar National Research Fund (grant numbers: NPRP-10-0126-170262 and NPRP11S-0121-180351 awarded to D.B.), by Sidra Precision Medicine Program (SDR400123 and SDR100035 awarded to D.B.), and by the AIRC IG 2018-ID 21846 grant awarded to M.C.

References

[1] Leisha A Emens, Paolo A Ascierto, Phillip K Darcy, Sandra Demaria, Alexander MM Eggermont, William L Redmond, Barbara Seliger, and Francesco M Marincola. Cancer immunotherapy: opportunities and challenges in the rapidly evolving clinical landscape. *European Journal of Cancer*, 81:116–129, 2017.

[2] Jun Gong, Alexander Chehrazi-Raffle, Srikanth Reddi, and Ravi Salgia. Development of pd-1 and pd-l1 inhibitors as a form of cancer immunotherapy: a comprehensive review of registration trials and future considerations. *Journal for immunotherapy of cancer*, 6(1):8, 2018.

[3] Jessica Roelands, Wouter Hendrickx, Gabriele Zoppoli, Raghvendra Mall, Mohamad Saad, Kyle Halliwill, Giuseppe Curigliano, Darawan Rinchai, Julie Decock, Lucia G Delogu, Tolga Turan, Josue Samayoa, Lotfi Chouchane, Alberto Ballestrero, Ena Wang, Pascal Finetti, François Bertucci, Lance D Miller, Jérôme Galon, Francesco M Marincola, Peter J K Kuppen, Michele Ceccarelli, and Davide Bedognetti. Oncogenic states dictate the prognostic and predictive connotations of intratumoral immune response. *Journal for ImmunoTherapy of Cancer*, 8(1), 2020.

[4] Razvan Cristescu, Robin Mogg, Mark Ayers, Andrew Albright, Erin Murphy, Jennifer Yearley, Xinwei Sher, Xiao Qiao Liu, Hongchao Lu, Michael Nebozhyn, et al. Pan-tumor genomic biomarkers for pd-1 checkpoint blockade–based immunotherapy. *Science*, 362(6411), 2018.

[5] Vésteinn Thorsson, David L Gibbs, Scott D Brown, Denise Wolf, Dante S Bortone, Tai-Hsien Ou Yang, Eduard Porta-Pardo, Galen F Gao, Christopher L Plaisier, James A Eddy, et al. The immune landscape of cancer. *Immunity*, 48(4):812–830, 2018.

[6] Davide Bedognetti, Michele Ceccarelli, Lorenzo Galluzzi, Rongze Lu, Karolina Palucka, Josue Samayoa, Stefani Spranger, Sarah Warren, Kwok-Kin Wong, Elad Ziv, et al. Toward a comprehensive view of cancer immune responsiveness: a synopsis from the sitc workshop. *Journal for immunotherapy of cancer*, 7(1):1–23, 2019.

[7] Davide Bedognetti, Wouter Hendrickx, Michele Ceccarelli, Lance D Miller, and Barbara Seliger. Disentangling the relationship between tumor genetic programs and immune responsiveness. *Current opinion in immunology*, 39:150–158, 2016.

[8] Jérôme Galon, Helen K Angell, Davide Bedognetti, and Francesco M Marincola. The continuum of cancer immunosurveillance: prognostic, predictive, and mechanistic signatures. *Immunity*, 39(1):11–26, 2013.

[9] Ena Wang, Davide Bedognetti, and Francesco M Marincola. Prediction of response to anticancer immunotherapy using gene signatures. *Journal of clinical oncology: official journal of the American Society of Clinical Oncology*, 31(19):2369, 2013.

[10] Wouter Hendrickx, Ines Simeone, Samreen Anjum, Younes Mokrab, François Bertucci, Pascal Finetti, Giuseppe Curigliano, Barbara Seliger, Luigi Cerulo, Sara Tomei, et al. Identification of genetic determinants of breast cancer immune phenotypes by integrative genome-scale analysis. *Oncoimmunology*, 6(2):e1253654, 2017.

[11] Tolga Turan, Deepti Kannan, Maulik Patel, J Matthew Barnes, Sonia G Tanlimco, Rongze Lu, Kyle Halliwill, Sarah Kongpachith, Douglas E Kline,

Wouter Hendrickx, et al. Immune oncology, immune responsiveness and the theory of everything. *Journal for immunotherapy of cancer*, 6(1):50, 2018.

[12] Mariya Rozenblit, Wouter Hendrickx, Adriana Heguy, Luis Chiriboga, Cynthia Loomis, Karina Ray, Farbod Darvishian, Mikala Egeblad, Sandra Demaria, Francesco M Marincola, et al. Transcriptomic profiles conducive to immune-mediated tumor rejection in human breast cancer skin metastases treated with imiquimod. *Scientific Reports*, 9(1):8572, 2019.

[13] François Bertucci, Pascal Finetti, Ines Simeone, Wouter Hendrickx, Ena Wang, Francesco M Marincola, Patrice Viens, Emilie Mamessier, Michele Ceccarelli, Daniel Birnbaum, et al. The immunologic constant of rejection classification refines the prognostic value of conventional prognostic signatures in breast cancer. *British journal of cancer*, 119(11):1383–1391, 2018.

[14] Davide Bedognetti. A multi-layer molecular fresco of the immune diversity across hematologic malignancies. *Cancer Cell*, 38(3):313–316, 2020.

[15] Olli Dufva, Petri Pölönen, Oscar Brück, Mikko AI Keränen, Jay Klievink, Juha Mehtonen, Jani Huuhtanen, Ashwini Kumar, Disha Malani, Sanna Siitonen, et al. Immunogenomic landscape of hematological malignancies. *Cancer cell*, 38(3):380–399, 2020.

[16] Stefani Spranger, Riyue Bao, and Thomas F Gajewski. Melanoma-intrinsic β-catenin signalling prevents anti-tumour immunity. *Nature*, 523(7559):231–235, 2015.

[17] Anusha Kalbasi and Antoni Ribas. Tumour-intrinsic resistance to immune checkpoint blockade. *Nature Reviews Immunology*, 20(1):25–39, 2020.

[18] Luz Garcia-Alonso, Francesco Iorio, Angela Matchan, Nuno Fonseca, Patricia Jaaks, Gareth Peat, Miguel Pignatelli, Fiammetta Falcone, Cyril H Benes, Ian Dunham, et al. Transcription factor activities enhance markers of drug sensitivity in cancer. *Cancer research*, 78(3):769–780, 2018.

[19] Francesco Iorio, Theo A Knijnenburg, Daniel J Vis, Graham R Bignell, Michael P Menden, Michael Schubert, Nanne Aben, Emanuel Gonçalves, Syd Barthorpe, Howard Lightfoot, et al. A landscape of pharmacogenomic interactions in cancer. *Cell*, 166(3):740–754, 2016.

[20] Matthew H Bailey, Collin Tokheim, Eduard Porta-Pardo, Sohini Sengupta, Denis Bertrand, Amila Weerasinghe, Antonio Colaprico, Michael C Wendl, Jaegil Kim, Brendan Reardon, et al. Comprehensive characterization of cancer driver genes and mutations. *Cell*, 173(2):371–385, 2018.

[21] Matias M Falco, Marta Bleda, José Carbonell-Caballero, and Joaquín Dopazo. The pan-cancer pathological regulatory landscape. *Scientific reports*, 6:39709, 2016.

[22] Wei Keat Lim, Eugenia Lyashenko, and Andrea Califano. Master regulators used as breast cancer metastasis classifier. In *Biocomputing 2009*, pages 504–515. World Scientific, 2009.

[23] Andrea Califano and Mariano J Alvarez. The recurrent architecture of tumour initiation, progression and drug sensitivity. *Nature reviews Cancer*, 17(2):116, 2017.

[24] Alexander Lachmann, Federico M Giorgi, Gonzalo Lopez, and Andrea Califano. Aracne-ap: gene network reverse engineering through adaptive partitioning inference of mutual information. *Bioinformatics*, 32(14):2233–2235, 2016.

[25] Alexandre Irrthum, Louis Wehenkel, Pierre Geurts, et al. Inferring regulatory networks from expression data using tree-based methods. *PloS one*, 5(9):e12776, 2010.

[26] Raghvendra Mall, Luigi Cerulo, Luciano Garofano, Veronique Frattini, Khalid Kunji, Halima Bensmail, Thais S Sabedot, Houtan Noushmehr, Anna Lasorella, Antonio Iavarone, et al. Rgbm: regularized gradient boosting machines for identification of the transcriptional regulators of discrete glioma subtypes. *Nucleic acids research*, 46(7):e39–e39, 2018.

[27] Raghvendra Mall, Luigi Cerulo, Halima Bensmail, Antonio Iavarone, and Michele Ceccarelli. Detection of statistically significant network changes in complex biological networks. *BMC systems biology*, 11(1):32, 2017.

[28] Raghvendra Mall, Ehsan Ullah, Khalid Kunji, Fulvio D'Angelo, Halima Bensmail, and Michele Ceccarelli. Differential community detection in paired biological networks. In *Proceedings of the 8th ACM International Conference on Bioinformatics, Computational Biology, and Health Informatics*, pages 330–339, 2017.

[29] Raghvendra Mall, Ehsan Ullah, Khalid Kunji, Michele Ceccarelli, and Halima Bensmail. An unsupervised disease module identification technique in biological networks using novel quality metric based on connectivity, conductance and modularity. *F1000Research*, 7(378):378, 2018.

[30] Véronique Frattini, Stefano M Pagnotta, Jerry J Fan, Marco V Russo, Sang Bae Lee, Luciano Garofano, Jing Zhang, Peiguo Shi, Genevieve Lewis, Heloise Sanson, et al. A metabolic function of fgfr3-tacc3 gene fusions in cancer. *Nature*, 553(7687):222, 2018.

[31] Fulvio DâĽ™Angelo, Michele Ceccarelli, Luciano Garofano, Jing Zhang, Véronique Frattini, Francesca P Caruso, Genevieve Lewis, Kristin D Alfaro, Luc Bauchet, Giulia Berzero, et al. The molecular landscape of glioma in patients with neurofibromatosis 1. *Nature medicine*, 25(1):176, 2019.

[32] Jason K Sa, Nakho Chang, Hye Won Lee, Hee Jin Cho, Michele Ceccarelli, Luigi Cerulo, Jinlong Yin, Sung Soo Kim, Francesca P Caruso, Mijeong Lee, et al. Transcriptional regulatory networks of tumor-associated macrophages that drive malignancy in mesenchymal glioblastoma. *Genome biology*, 21(1):1–17, 2020.

[33] Daniel Marbach, James C Costello, Robert Küffner, Nicole M Vega, Robert J Prill, Diogo M Camacho, Kyle R Allison, Manolis Kellis, James J Collins, and Gustavo Stolovitzky. Wisdom of crowds for robust gene network inference. *Nature methods*, 9(8):796–804, 2012.

[34] Mariano J Alvarez, Yao Shen, Federico M Giorgi, Alexander Lachmann, B Belinda Ding, B Hilda Ye, and Andrea Califano. Functional characterization of somatic mutations in cancer using network-based inference of protein activity. *Nature genetics*, 48(8):838–847, 2016.

[35] Mariano J Alvarez, Yao Shen, Federico M Giorgi, Alexander Lachmann, B Belinda Ding, B Hilda Ye, and Andrea Califano. Network-based inference of protein activity helps functionalize the genetic landscape of cancer. *Nature genetics*, 48(8):838, 2016.

[36] Aravind Subramanian, Pablo Tamayo, Vamsi K Mootha, Sayan Mukherjee, Benjamin L Ebert, Michael A Gillette, Amanda Paulovich, Scott L Pomeroy, Todd R Golub, Eric S Lander, et al. Gene set enrichment analysis: a knowledge-based approach for interpreting genome-wide expression profiles. *Proceedings of the National Academy of Sciences*, 102(43):15545–15550, 2005.

[37] Sonja Hänzelmann, Robert Castelo, and Justin Guinney. Gsva: gene set variation analysis for microarray and rna-seq data. *BMC bioinformatics*, 14(1):7, 2013.

[38] Alexey Sergushichev. An algorithm for fast preranked gene set enrichment analysis using cumulative statistic calculation. *BioRxiv*, https://doi.org/10.1101/060012:06–12, 2016.

[39] Mehmet Eren Ahsen, Yoojin Chun, Alexander Grishin, Galina Grishina, Gustavo Stolovitzky, Gaurav Pandey, and Supinda Bunyavanich. Netfactor, a framework for identifying transcriptional regulators of gene expression-based biomarkers. In *Proceedings of the 11th ACM International Conference on Bioinformatics, Computational Biology and Health Informatics*, pages 1–13, 2020.

[40] Andrew J Gentles, Aaron M Newman, Chih Long Liu, Scott V Bratman, Weiguo Feng, Dongkyoon Kim, Viswam S Nair, Yue Xu, Amanda Khuong, Chuong D Hoang, et al. The prognostic landscape of genes and infiltrating immune cells across human cancers. *Nature medicine*, 21(8):938–945, 2015.

[41] Atanas Kamburov, Konstantin Pentchev, Hanna Galicka, Christoph Wierling, Hans Lehrach, and Ralf Herwig. Consensuspathdb: toward a more complete picture of cell biology. *Nucleic acids research*, 39(suppl_1):D712–D717, 2010.

[42] Michael Ashburner, Catherine A Ball, Judith A Blake, David Botstein, Heather Butler, J Michael Cherry, Allan P Davis, Kara Dolinski, Selina S Dwight, Janan T Eppig, et al. Gene ontology: tool for the unification of biology. *Nature genetics*, 25(1):25–29, 2000.

[43] Evan O Paull, Alvaro Aytes, Sunny J Jones, Prem S Subramaniam, Federico M Giorgi, Eugene F Douglass, Somnath Tagore, Brennan Chu, Alessandro Vasciaveo, Siyuan Zheng, et al. A modular master regulator landscape controls cancer transcriptional identity. *Cell*, 184(2):334–351, 2021.

[44] Joshua Broyde, David R Simpson, Diana Murray, Evan O Paull, Brennan W Chu, Somnath Tagore, Sunny J Jones, Aaron T Griffin, Federico M Giorgi, Alexander Lachmann, et al. Oncoprotein-specific molecular interaction maps (sigmaps) for cancer network analyses. *Nature Biotechnology*, pages 1–10, 2020.

[45] Vartika Mishra, Diane B Re, Virginia Le Verche, Mariano J Alvarez, Alessandro Vasciaveo, Arnaud Jacquier, Paschalis-Tomas Doulias, Todd M Greco, Monica Nizzardo, Dimitra Papadimitriou, et al. Systematic elucidation of neuron-astrocyte interaction in models of amyotrophic lateral sclerosis using multi-modal integrated bioinformatics workflow. *Nature communications*, 11(1):1–20, 2020.

[46] Benjamin M Bolstad, Rafael A Irizarry, Magnus Åstrand, and Terence P. Speed. A comparison of normalization methods for high density oligonucleotide array data based on variance and bias. *Bioinformatics*, 19(2):185–193, 2003.

[47] Tolga Turan, Sarah Kongpachith, Kyle Halliwill, Jessica Roelands, Wouter Hendricks, Francesco M Marincola, Thomas J Hudson, Howard J Jacob, Davide Bedognetti, Josue Samayoa, et al. A balance score between immune stimulatory and suppressive microenvironments identifies mediators of tumour immunity and predicts pan-cancer survival. *British journal of cancer*, pages 1–10, 2020.

[48] Ena Wang, Andrea Worschech, and Francesco M Marincola. The immunologic constant of rejection. *Trends in immunology*, 29(6):256–262, 2008.

[49] Gordon K Smyth. Linear models and empirical bayes methods for assessing differential expression in microarray experiments. *Statistical applications in genetics and molecular biology*, 3(1), 2004.

[50] Yoav Benjamini and Yosef Hochberg. Controlling the false discovery rate: a practical and powerful approach to multiple testing. *Journal of the Royal statistical society: series B (Methodological)*, 57(1):289–300, 1995.

[51] Ralf Herwig, Christopher Hardt, Matthias Lienhard, and Atanas Kamburov. Analyzing and interpreting genome data at the network level with consensuspathdb. *Nature protocols*, 11(10):1889, 2016.

[52] Atanas Kamburov, Ulrich Stelzl, Hans Lehrach, and Ralf Herwig. The consensuspathdb interaction database: 2013 update. *Nucleic acids research*, 41(D1):D793–D800, 2013.

[53] Da Wei Huang, Brad T Sherman, and Richard A Lempicki. Systematic and integrative analysis of large gene lists using david bioinformatics resources. *Nature protocols*, 4(1):44, 2009.

[54] Alison M Taylor, Juliann Shih, Gavin Ha, Galen F Gao, Xiaoyang Zhang, Ashton C Berger, Steven E Schumacher, Chen Wang, Hai Hu, Jianfang Liu, et al. Genomic and functional approaches to understanding cancer aneuploidy. *Cancer cell*, 33(4):676–689, 2018.

[55] Dvir Aran, Marina Sirota, and Atul J Butte. Systematic pan-cancer analysis of tumour purity. *Nature communications*, 6(1):1–12, 2015.

[56] Qiuzhen Liu, Sara Tomei, Maria Libera Ascierto, Valeria De Giorgi, Davide Bedognetti, Cuilian Dai, Lorenzo Uccellini, Tara Spivey, Zoltan Pos, Jaime Thomas, et al. Melanoma nos1 expression promotes dysfunctional ifn signaling. *The Journal of clinical investigation*, 124(5):2147–2159, 2014.

[57] Tara L Spivey, Valeria De Giorgi, Yingdong Zhao, Davide Bedognetti, Zoltan Pos, Qiuzhen Liu, Sara Tomei, Maria Libera Ascierto, Lorenzo Uccellini, Jennifer Reinboth, et al. The stable traits of melanoma genetics: an alternate approach to target discovery. *BMC genomics*, 13(1):1–11, 2012.

[58] Joseph L Benci, Lexus R Johnson, Ruth Choa, Yuanming Xu, Jingya Qiu, Zilu Zhou, Bihui Xu, Darwin Ye, Katherine L Nathanson, Carl H June, et al. Opposing functions of interferon coordinate adaptive and innate immune responses to cancer immune checkpoint blockade. *Cell*, 178(4):933–948, 2019.

[59] Akihiko Yoshimura and Go Muto. Tgf-β function in immune suppression. In *Negative Co-Receptors and Ligands*, pages 127–147. Springer, 2010.

[60] Viviana E Hermosilla, Matias I Hepp, David Escobar, Carlos Farkas, Elizabeth N Riffo, Ariel F Castro, and Roxana Pincheira. Developmental sall2 transcription factor: a new player in cancer. *Carcinogenesis*, 38(7):680–690, 2017.

[61] Pan Wang, Xinhua Song, Dan Cao, Kairong Cui, Jingxiao Wang, Kirsten Utpatel, Runze Shang, Haichuan Wang, Li Che, Matthias Evert, et al. Oncogene-dependent function of brg1 in hepatocarcinogenesis. *Cell death & disease*, 11(2):1–11, 2020.

[62] Jason J Luke, Riyue Bao, Randy F Sweis, Stefani Spranger, and Thomas F Gajewski. Wnt/β-catenin pathway activation correlates with immune exclusion across human cancers. *Clinical Cancer Research*, 25(10):3074–3083, 2019.

[63] C Massard, A Azaro, J-C Soria, U Lassen, C Le Tourneau, D Sarker, C Smith, U Ohnmacht, G Oakley, BKR Patel, et al. First-in-human study of ly3039478, an oral notch signaling inhibitor in advanced or metastatic cancer. *Annals of Oncology*, 29(9):1911–1917, 2018.

[64] Grégoire de Streel, Charlotte Bertrand, Nicolas Chalon, Stéphanie Liénart, Orian Bricard, Sara Lecomte, Julien Devreux, Mélanie Gaignage, Gitte De Boeck, Lore Mariën, et al. Selective inhibition of tgf-β1 produced by garp-expressing tregs overcomes resistance to pd-1/pd-l1 blockade in cancer. *Nature communications*, 11(1):1–15, 2020.

[65] Rosalyn W Sayaman, Mohamad Saad, Vésteinn Thorsson, Donglei Hu, Wouter Hendrickx, Jessica Roelands, Eduard Porta-Pardo, Younes Mokrab, Farshad Farshidfar, Tomas Kirchhoff, et al. Germline genetic contribution to the immune landscape of cancer. *Immunity*, 54(2):367–386, 2021.

[66] Liang-Bo Wang, Alla Karpova, Marina A Gritsenko, Jennifer E Kyle, Song Cao, Yize Li, Dmitry Rykunov, Antonio Colaprico, Joseph H Rothstein, Runyu Hong, et al. Proteogenomic and metabolomic characterization of human glioblastoma. *Cancer Cell*, 2021.
