# Supplementary material for: Network-based identification of key master regulators associated with an immune-silent cancer phenotype
Source: Brief Bioinform. 2021 May 13;22(6):bbab168. doi: 10.1093/bib/bbab168 (PMC8574720; doi:10.1093/bib/bbab168)
Supplement: Supplementary_Information_for_Network_based_Identification_of_Key_MRs_for_ICR_bbab168 [file supplementary_information_for_network_based_identification_of_key_mrs_for_icr_bbab168.zip › Supplementary_Information_for_Network_based_Identification_of_Key_MRs_for_ICR_bbab168.pdf]

Supplementary Information for

Network-based Identification of Key Master Regulators associated with an Immune-Silent Cancer Phenotype

Raghvendra Mall, Mohamad Saad, Jessica Roelands, et al

Corresponding Author: Raghvendra Mall and Davide Bedogentti.
Email: rmall@hbku.edu.qa and dbedognetti@sidra.org

This PDF file includes:

Figs. S1 to S9
Tables S1 to S13
SI References

1. Methods

A. Inferring Gene Regulatory Networks. Regularized Gradient Boosting Machine (RGBM) (1) belongs to the class of machine learning techniques based on feature selection where the expression vector of each target genes (i.e t) is considered as dependent variable ($Y_t = g_t^c$) and the expression matrix corresponding to the list of transcription regulators (TR) are the independent variables (X_{TR}). The goal of RGBM is to detect linear/non-linear TR-target interactions using a gradient boosting procedure (2) with a decision tree (3) as a base learner. ARACNE (4) on the other-hand is based on concepts of mutual information ($\mathrm{MI}(g_{TR}^c, g_t^c)$) and prevents indirect transitive interactions using an information-theoretic property, the data processing inequality. Using a bootstrapping procedure, ARACNE can also provide the strength (in terms of statistical significance) of a TR-target interaction.

B. Scoring TR activities. In RGBM, the regulon of a TR (see Fig 1B in main manuscript) was divided into positively regulated targets and negatively regulated targets by performing a Pearson correlation between the expression of the TR (g_{TR}^c) and the expression of the target genes (g_t^c) in its regulon across all the samples for that cancer c (see Fig 1C in main manuscript). The targets with positive correlations were considered as activated targets and the targets with negative correlations were identified as repressed targets in the TR's regulon. Mathematically, the activity of each TR (Fig 1D) can be defined as:

$$Act^c(\mathrm{TR}, i) = \frac{1}{U}\sum_{k=1}^{U} g_{k,i}^{c,+} - \frac{1}{V}\sum_{j=1}^{V} g_{j,i}^{c,-}.$$

Here $Act^c(\mathrm{TR}, i)$ represents the activity of a TR in the ith tumor sample for a particular cancer c, U and V represent the number of positive and negative targets in that TR regulon respectively and $g_{k,i}^{c,+}$,corresponds to the mRNA levels of the kth activated target gene whereas $g_{j,i}^{c,-}$ stands for the expression of the jth repressed target gene in the tumor sample i obtained from the regulon of that TR in cancer c. This simplistic formula for TR activity calculation was shown to be effective for the identification of differentially active TRs (MRs) in (1).

C. Gene-Set Enrichment Analysis. In VIPER (5), a normalized enrichment score (NES) is computed analytically, based on the assumption that in the null situation, the target genes are uniformly distributed on the gene expression signature. Since there is extensive co-regulation of gene expression taking place in the cell, this assumption never holds true, and this is the reason why a null model based on sample permutations is used. In order to generate NES for TRs in ICR High samples, we use the ICR Medium samples as a set of reference samples, and the corresponding null model based on sample permutations can be obtained with the function 'viperSignature' in the 'viper' R package (https://doi.org/doi:10.18129/B9.bioc.viper). Similarly, to generate the NES for TRs in ICR Low samples, we again use the ICR Medium samples as a set of reference samples.

FGSEA (6) implements a special algorithm to calculate the empirical NES null distributions simultaneously for all the gene-set sizes (TR regulons), which allows up to several hundred times faster execution time compared to original GSEA (7) implementation. This also enables FGSEA to provide statistical significance associated with the NES scores for TRs.

2. Results

A. GRN Comparison. The median number of edges common to RGBM and ARACNE in the reverse engineered GRNs for a cancer c was $69,056$ ($[46368, 88568]$). We used the Jaccard coefficient to calulcate similarity between inferred networks defined as:

$$\mathcal{J} = \frac{n(\mathcal{G}_{\mathrm{RGBM}}^c \cap \mathcal{G}_{\mathrm{ARACNE}}^c)}{n(\mathcal{G}_{\mathrm{RGBM}}^c \cup \mathcal{G}_{\mathrm{ARACNE}}^c)}$$

The median value of Jaccard coefficient ($\mathcal{J}$) (8) was 0.2185 ([0.139,0.233]). Here G_{RGBM}^c represented the GRN inferred via RGBM and G_{ARACNE}^c correspond to the GRN determined using ARACNE. The Jaccard coefficient is a measure of similarity between two networks (in terms of common TR-target gene regulations or edges) and takes values between $[0, 1]$, where higher values indicate more similarity. From Supp. Tables S1 and S2, we observed that for c with a large number of samples, the inferred GRNs tend to have higher Jaccard coefficient. This suggested that with the availability of more samples per c, different GRN inference techniques could potentially converge to similar sets of edges (TR-target interactions).

B. Consensus MRs. We obtained a total of 661, 542, 452, 342, 437, 150, 560 and 236 consensus MRs for ICR-E cancers BLCA, BRCA, HNSC, LIHC (Liver hepatocellular carcinoma), SARC (Sarcoma), SKCM, STAD (Stomach adenocarcinoma) and UCEC (Uterine Corpus Endometrial Carcinoma) respectively. Similarly, we obtained a total of 616, 453, 481 and 327 consensus MRs for ICR-D cancers LGG, KIRC (Kidney renal clear cell carcinoma), PAAD (Pancreatic adenocarcinoma) and UVM (Uveal Melanoma) respectively.

Table S1. Standard network properties of the gene regulatory networks inferred by RGBM for the 12 cancer subtypes of interest. For all the GRNs, the average number of target genes that a TR regulates is in between [45,75], whereas the average number of TRs regulating one target gene is in between [7,12]. The total number of differentially active master regulators (MRs) for ICR Enabled cancers (in bold) with |NES|>1.0 are in the range [475,773] with average being approximately 628 whereas in the case of ICR Disabled cancer (in italics), the total number of MRs vary in the range [474,858] with the average being approximately 665 (higher than ICR Enabled cancers). The average clustering coefficient for the inferred GRNs for all cancers is around 0.1 except for SARC (0.006) where it is exceptionally small indicating the absence of communities/clusters. The network diameter of all the inferred GRNs for the 12 cancers lies in the range [10.17] whereas the average path length across these networks is consistently around 4.5. We compare the GRNs inferred by RGBM with those reconstructed using ARACNE and highlight the edges in common (Intersection Edges). For cancer types with availability of large number of ICR High and ICR Low samples (see Supp. Table S2), the number of interactions between TRs and target genes are lower in comparison to those cancer types with fewer samples (SKCM, PAAD and UVM), thereby, reducing the chances of having false-positive regulations.

Cancer Type	Total Edges	Avg. Outdegree (Targets regulated by a TR)	Avg. Indegree (TRs regulating a target)	No of Top MRs	Avg. Clustering Coefficient	Network Diameter	Avg. Path Length
**BLCA**	225337	61.333	9.71	773	0.117	11	4.37
**BRCA**	167522	45.6	7.22	700	0.151	17	5.22
**HNSC**	206207	56.13	8.88	622	139	15	4.66
**LIHC**	226440	61.63	9.74	600	0.111	14	4.31
**SARC**	250236	68.11	10.78	559	0.006	10	4.08
**SKCM**	264501	71.99	11.39	475	0.091	11	3.96
**STAD**	228356	62.15	9.84	772	0.127	14	4.37
**UCEC**	225648	61.42	9.72	521	0.113	13	4.31
LGG	213307	58.06	9.19	725	0.134	12	4.44
KIRC	227083	61.81	9.78	604	0.118	15	4.38
PAAD	259085	70.52	11.16	858	0.111	11	4.06
UVM	259288	70.57	11.17	474	0.095	12	3.97

Cancer Type	Intersection Edges	Union Edges	ARACNE Specific Edges	RGBM Specific Edges	Jaccard Coefficient
**BLCA**	66,757	307,065	82,038	158,580	0.213
**BRCA**	46,368	198,654	32,213	121,154	0.233
**HNSC**	62,910	269,828	64,658	143,297	0.233
**LIHC**	68,660	312,131	86,274	157,780	0.220
**SARC**	76,662	391,360	141,265	173,574	0.196
**SKCM**	88,568	544,816	280,315	175,933	0.163
**STAD**	73,505	318,866	90,759	154,851	0.231
**UCEC**	66,546	287,756	62,793	159,102	0.231
LGG	62,869	285,408	72,476	150,438	0.220
KIRC	69,452	320,704	94,209	157,631	0.217
PAAD	83,035	490,588	231,515	176,050	0.169
UVM	82,542	602,939	343,651	176746	0.139

Table S2. Total number of samples belonging to each of the 12 histologies. Here the cancer types in bold represent the ICR Enabled cancers whereas the italicized cancer types correspond to the ICR Disabled cancers.

Cancer Type	Total Samples	Total ICR High Samples	Total ICR Medium Samples	Total ICR Low Samples
**BLCA**	408	149	127	132
**BRCA**	1085	154	764	167
**HNSC**	500	166	130	204
**LIHC**	371	63	165	143
**SARC**	259	47	104	108
**SKCM**	103	19	33	51
**STAD**	374	54	212	108
**UCEC**	174	72	56	46
LGG	511	101	197	213
KIRC	530	74	327	129
PAAD	177	40	100	37
UVM	80	7	50	23

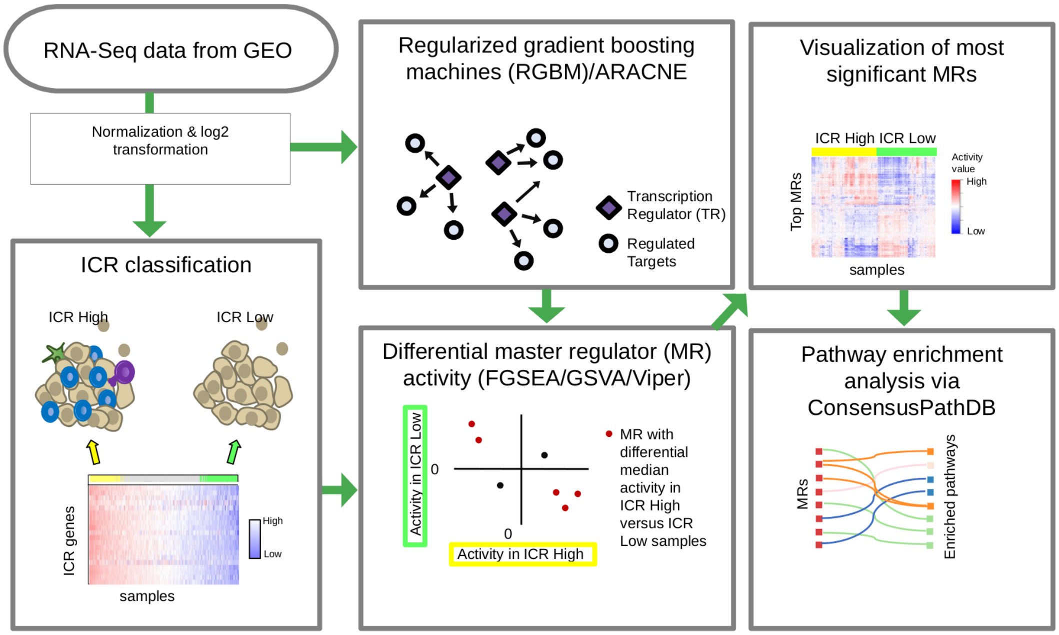

(a) Flow diagram of the pipeline followed in our network-based approach.

Comparison of RGBM and RGENIE with a other of inference methods on DREAM 3 and DREAM 4 networks of size 100

Methods	Data used	DREAM 3 experiments									
		Network 1		Network 2		Network 3		Network 4		Network 5	
		AU_{pr}	AU_{roc}	AU_{pr}	AU_{roc}	AU_{pr}	AU_{roc}	AU_{pr}	AU_{roc}	AU_{pr}	AU_{roc}
RGBM (LS-Boost)	KO,KD,WT	**0.699**	0.903	**0.888**	**0.965**	**0.597**	0.900	**0.571**	**0.861**	**0.460**	**0.787**
RGBM (LAD-Boost)	KO,KD,WT	0.683	0.903	0.870*	0.963*	0.562*	0.900	0.535*	0.853*	0.400	0.770
ENNET	KO,KD,WT,MTS	0.627	0.901	0.865^{+}	0.963^{+}	0.552^{+}	0.892	0.522^{+}	0.842	0.384	0.765
RGENIE	KO,KD,WT	0.521	0.870	0.821^{-}	0.899	0.456	0.812	0.478^{-}	0.778	0.356	0.718
GENIE	KO,KD,WT	0.430	0.850	0.782	0.883	0.372	0.729	0.423	0.724	0.314	0.656
iRafNet	KO,KD,WT	0.528	0.878	0.812	0.901	0.484	0.864	0.482	0.772	0.364	0.736
ARACNE	KO,KD,WT	0.348	0.781	0.656	0.813	0.285	0.669	0.396	0.662	0.274	0.583
Winner (72)	KO, WT	0.694	**0.948**	0.806	0.960	0.493	**0.915**	0.469	0.853	0.433	0.783
**Methods**	**Data Used**	DREAM 4 Experiments									
		**Network 1**		**Network 2**		**Network 3**		**Network 4**		**Network 5**	
		AU_{pr}	AU_{roc}	AU_{pr}	AU_{roc}	AU_{pr}	AU_{roc}	AU_{pr}	AU_{roc}	AU_{pr}	AU_{roc}
RGBM (LS-Boost)	KO,KD,WT,MTS	**0.709**	**0.936**	**0.561**	0.878*	**0.525**	**0.911**	**0.616**	**0.903**	**0.450**	**0.893**
RGBM (LAD-Boost)	KO,KD,WT,MTS	0.682*	0.924*	0.525*	**0.895**	0.490*	0.907*	0.566*	**0.903**	0.413*	0.885*
ENNET	KO,KD,WT	0.604^{+}	0.893	0.456^{+}	0.856^{+}	0.421^{+}	0.865^{+}	0.506^{+}	0.878^{+}	0.264^{+}	0.828^{+}
RGENIE	KO,WT	0.448	0.902	0.330	0.792	0.374	0.834^{-}	0.362^{-}	0.840	0.218^{-}	0.773^{-}
GENIE	KO,WT	0.338	0.864	0.309	0.748	0.277	0.782	0.267	0.808	0.114	0.720
iRafNet	KO,TS	0.552	0.901	0.337	0.799	0.414	0.835	0.421	0.847	0.298	0.792
ARACNE	KO,KD,WT	0.279	0.781	0.256	0.691	0.205	0.669	0.196	0.699	0.074	0.583
Winner (73)	KO	0.536	0.914	0.377	0.801	0.390	0.833	0.349	0.842	0.213	0.759

Here, we provide the mean AU_{pr} and AU_{roc} values for 10 random runs of different inference methods. Here, KO, knockout; KD, knockdown; WT, wildtype; MTS, modif ed smoothed version of the time-series data. The best results are highlighted in bold. *, +, − represent the quality metric values where RGBM (LAD-Boost), ENNET and RGENIE techniques, respectively outperform the winner of DREAM 3 and DREAM 4 challenges.

Comparison of RGBM and RGENIE with inference methods on DREAM 5 networks of varying sizes

Methods	Data used	DREAM 5 experiments					
		Network 1		Network 3		Network 4	
		AU_{pr}	AU_{roc}	AU_{pr}	AU_{roc}	AU_{pr}	AU_{roc}
RGBM (LS-Boost)	KO,Exp	**0.537**	0.846*	0.086	0.633*	**0.048**	**0.546**
RGBM (LAD-Boost)	KO,Exp	0.513*	0.842*	0.084	0.628*	0.047*	0.544*
ENNET	KO,Exp	0.432^{+}	**0.857**	0.069	0.632^{+}	0.021	0.532^{+}
iRafNet	KO,MTS,Exp	0.364	0.813	**0.112**	**0.641**	0.021	0.523
RGENIE	Exp	0.343^{-}	0.821^{-}	0.104^{-}	0.623^{-}	0.022^{-}	0.524^{-}
GENIE (Winner)	Exp	0.291	0.814	0.094	0.619	0.021	0.517
TIGRESS (15)	KO,Exp	0.301	0.782	0.069	0.595	0.020	0.517
CLR (18)	Exp	0.217	0.666	0.050	0.538	0.018	0.505
ARACNE	Exp	0.099	0.545	0.029	0.512	0.017	0.500

Here, we provide the mean AU_{pr} and AU_{roc} values for 10 random runs of different inference methods. Here, KO, knockout; KD, knockdown; WT, wildtype; MTS, modif ed smoothed version of the time-series data; Exp, steady-state gene expression. The best results are highlighted in bold. *, + and − represent the quality metric values where RGBM, ENNET and RGENIE techniques respectively defeat the winner of DREAM 5 challenge, i.e. GENIE.

(b) Comparison of RGBM with other state-of-the-art gene regulatory network reconstruction techniques including GENIE (3) and ARACNE (4) obtained from RGBM paper (1). Here it is highlighted that how RGBM outperforms techniques such as GENIE and ARACNE on several synthetic datasets.

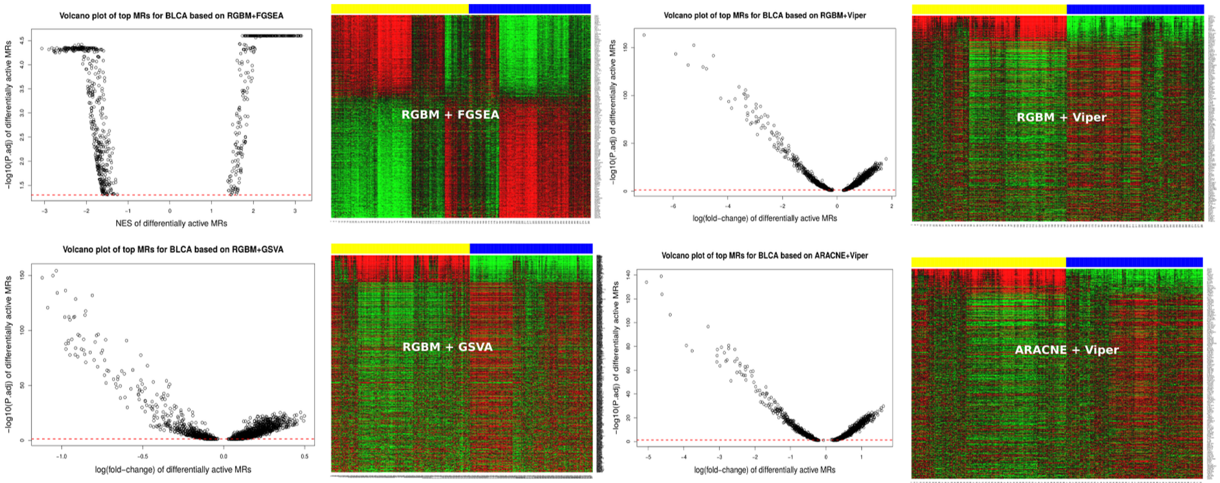

(c) Comparison of 4 different methods including RGBM + FGSEA, RGBM + GSVA, RGBM + Viper and ARACNE + Viper to identify the most differentially active MRs between ICR High and ICR Low samples for ICR Enabled cancer type BLCA.

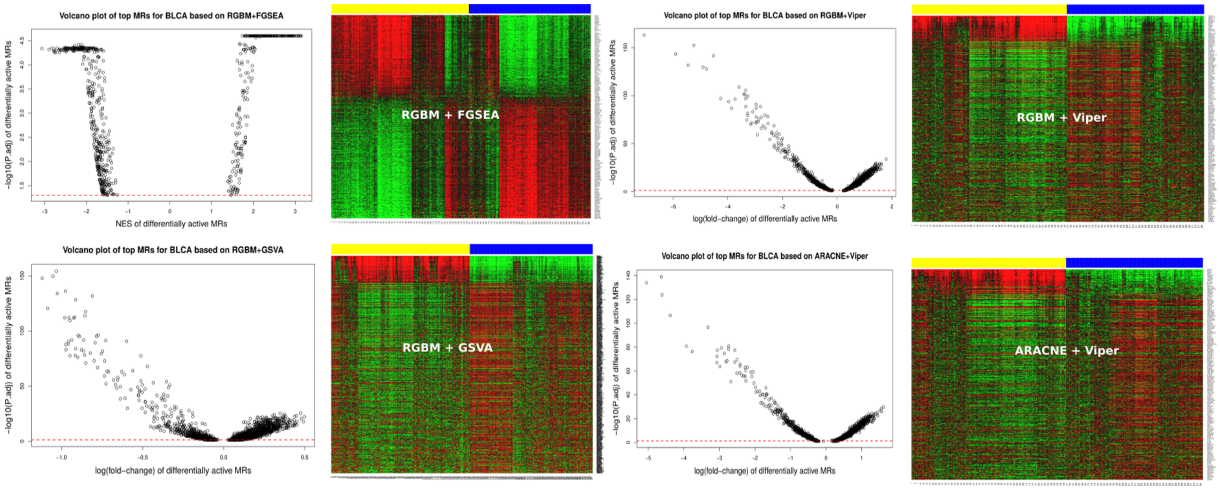

(d) Comparison of 4 different methods including RGBM + FGSEA, RGBM + GSVA, RGBM + Viper and ARACNE + Viper to identify the most differentially active MRs between ICR High and ICR Low samples for ICR Disabled cancer type LGG.

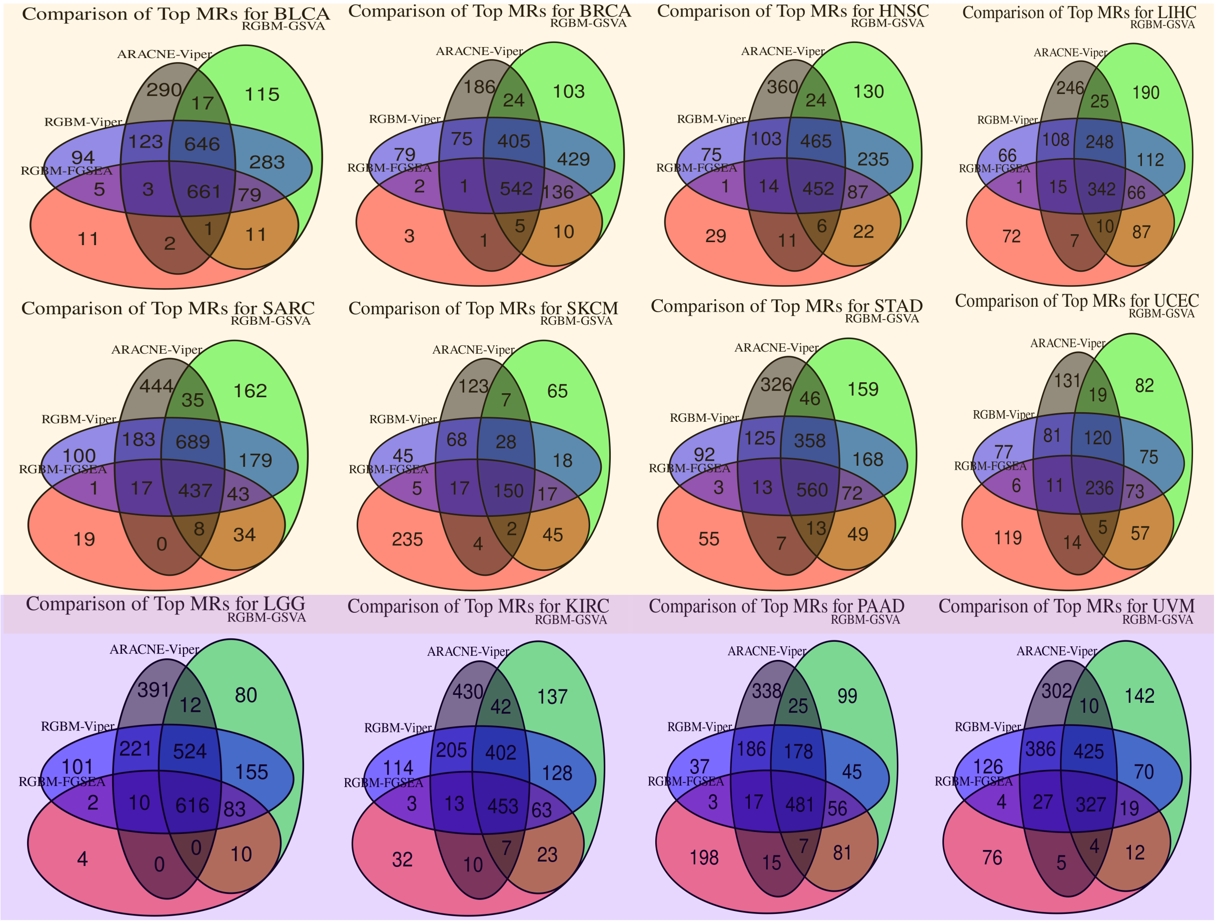

(e) The MRs which are common to the 4 different pipelines used for identification of the differential MRs w.r.t. their activity between ICR High and ICR Low samples for the 12 cancers of interest (including 8 ICR Enabled cancers and 4 ICR Disabled cancers).

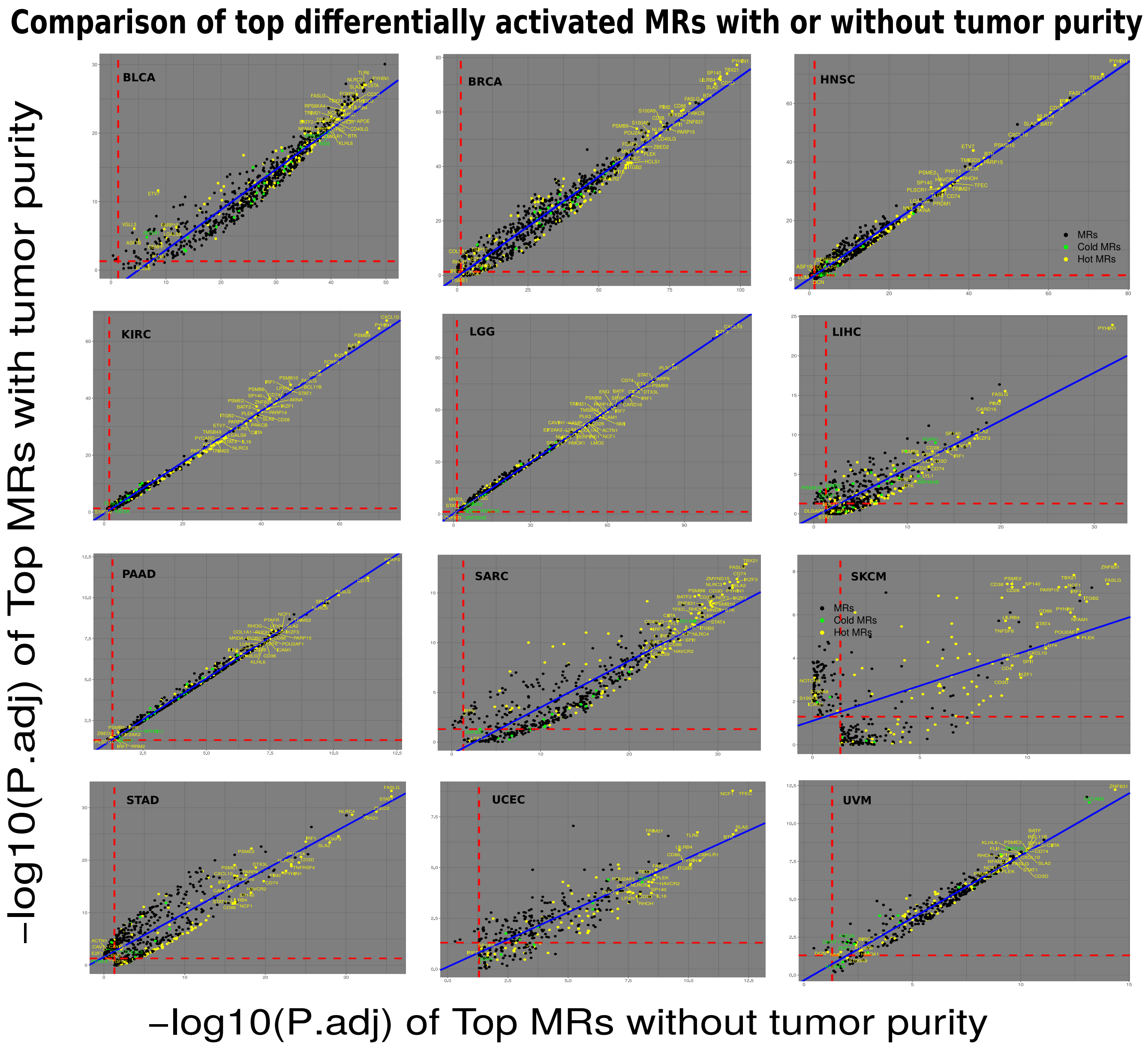

(f) We illustrate that the top differentially active MRs w.r.t. ICR High vs ICR Low identified without considering tumor purity remain intact when we take tumor purity (in each tumor sample estimated using ABSOLUTE algorithm (9)) into consideration for the majority of the 12 cancers of interest (except for cancer with small sample sizes such as SKCM and UCEC). Here the 'red' dotted lines highlight the FDR-adjusted p-value of 0.05 for significance. The 'yellow' and 'green' MRs correspond to the top MRs identified by our consensus framework to be specific to ICR High (hot MRs) and ICR Low (cold MRs) phenotype respectively and having either the lowest (bottom 10 percentile) or the highest (top 10 percentile) -$\log_{10}$(p-values).

Comparison of top differentially activated MRs with or without tumor purity

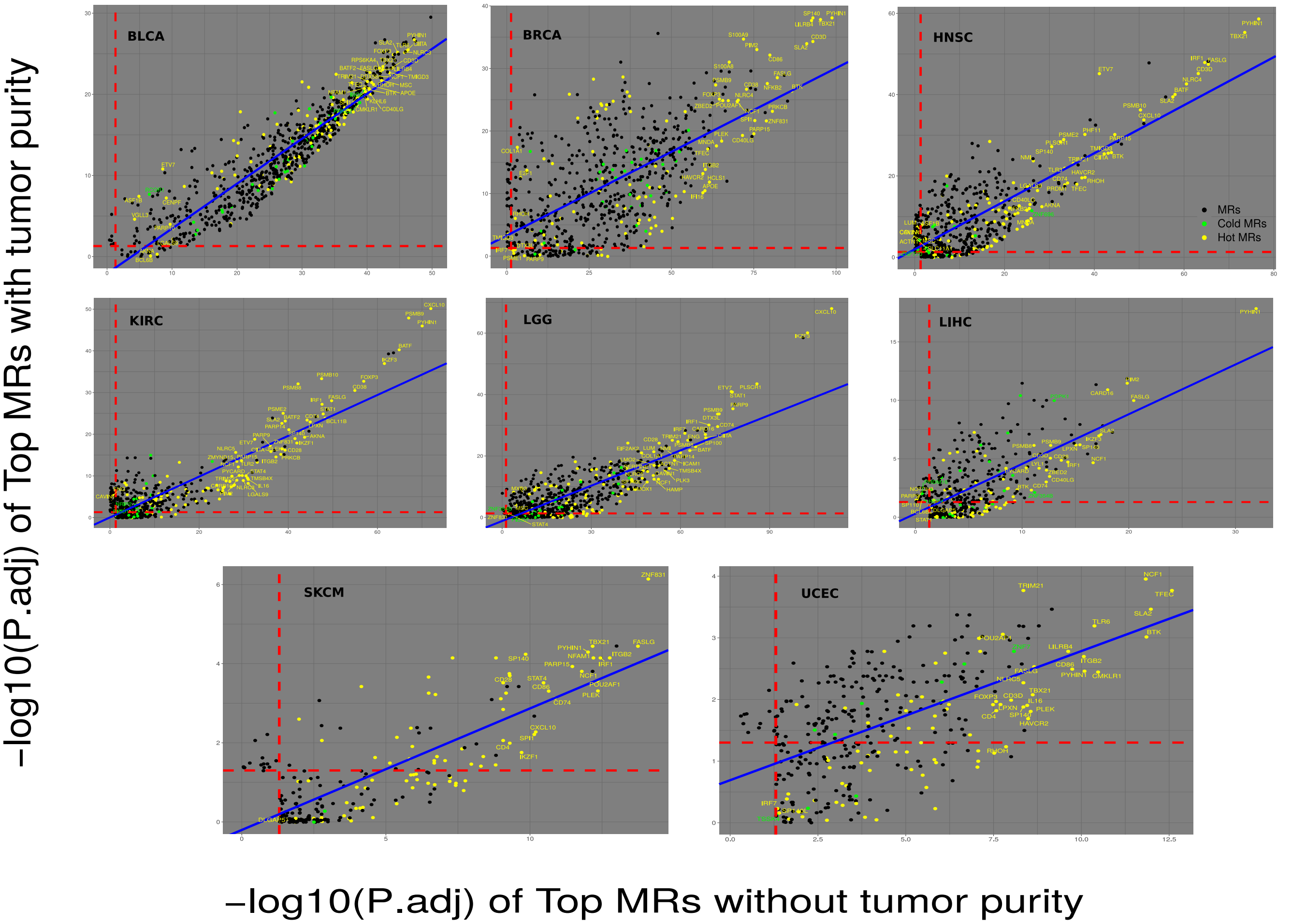

(g) We illustrate that the top differentially active MRs w.r.t. ICR High vs ICR Low identified without considering tumor purity remain intact when we take tumor purity (in each tumor sample estimated using the consensus algorithm proposed in (10)) into consideration for the majority of the 12 cancers of interest (except for cancer with small sample sizes such as SKCM and UCEC). Here the 'red' dotted lines highlight the FDR-adjusted p-value of 0.05 for significance. The 'yellow' and 'green' MRs correspond to the top MRs identified by our consensus framework to be specific to ICR High (hot MRs) and ICR Low (cold MRs) phenotype respectively and having either the lowest (bottom 10 percentile) or the highest (top 10 percentile) $-\log_{10}$(p-values).

Fig. S1. The pipeline used to identify the set of MRs which have significantly differential activities between ICR High and ICR Low samples for each cancer of interest.

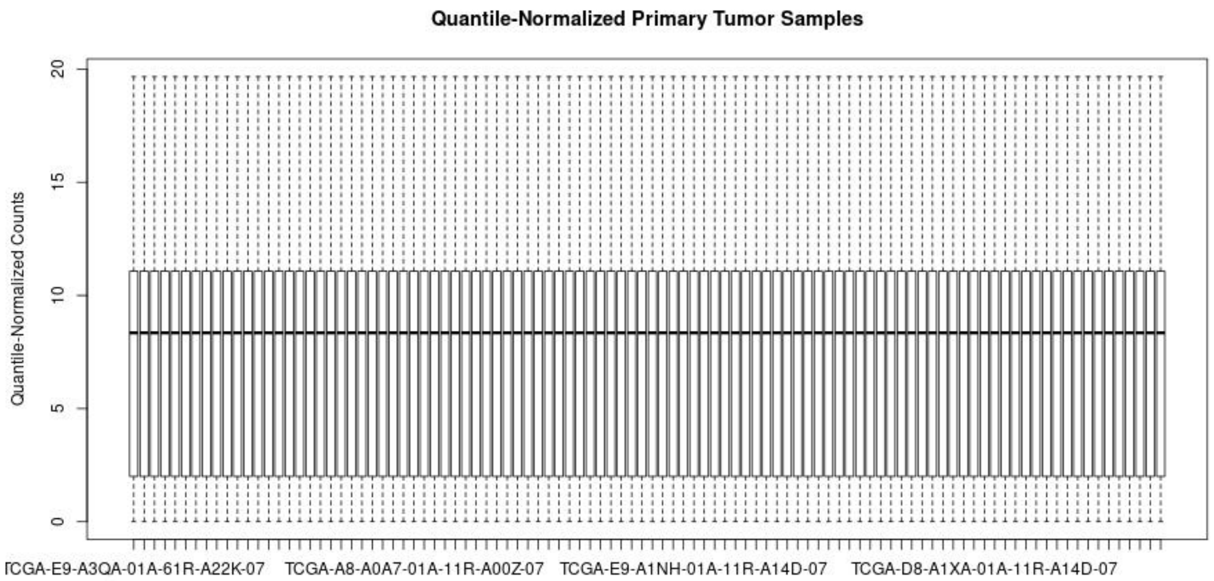

Fig. S2. Boxplot showcasing that variance in expression of all the genes for each tumor sample remains constant within a cancer, thereby resulting in quantile-normalized primary tumor samples for that cancer.

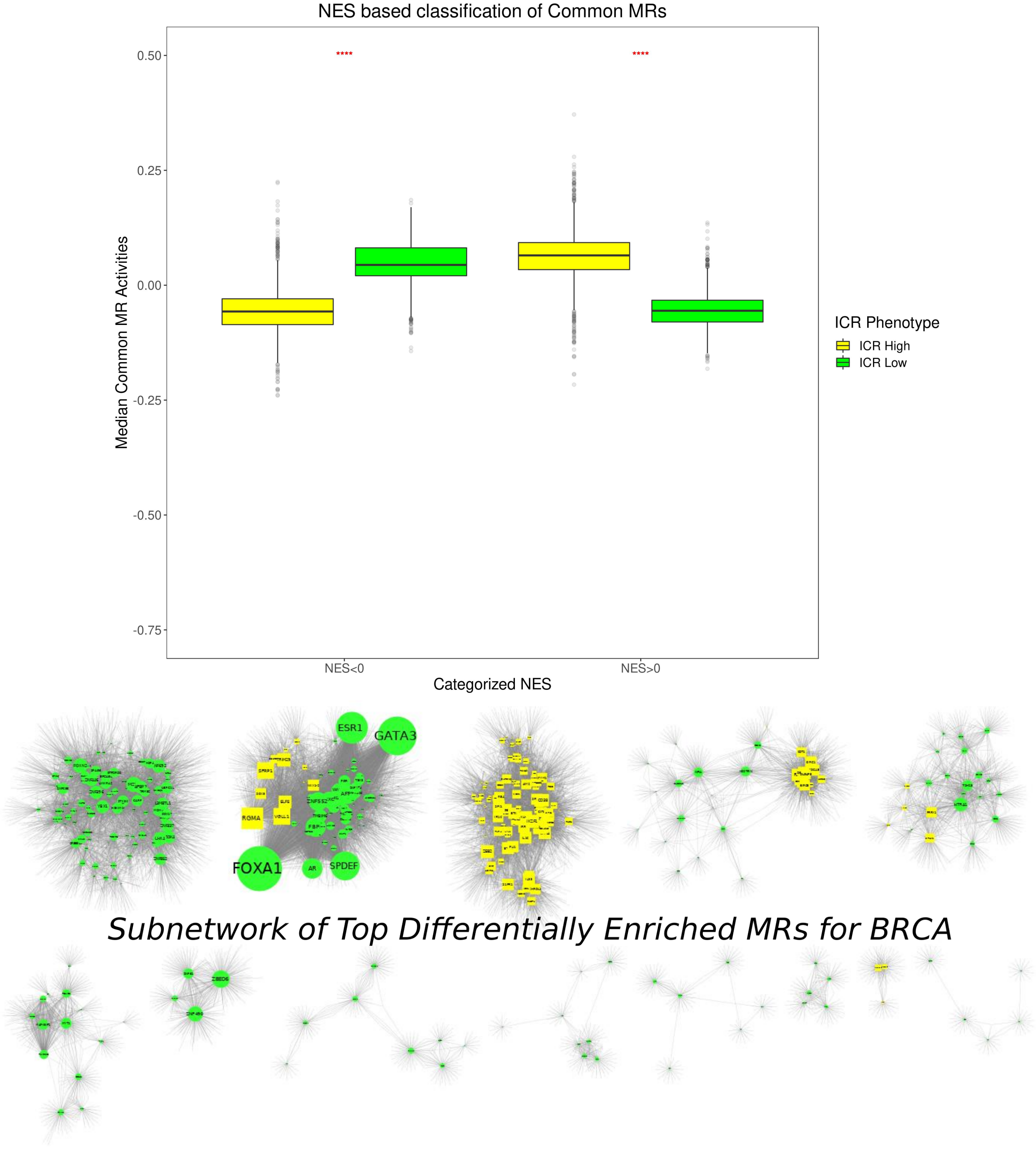

Fig. S3. Here we highlight that when the normalized enrichment scores (NES) for TRs are positive then, these TRs have high positive activity in ICR High samples and high negative activity in ICR Low samples. Thus, TRs with positive NES score are more specific to the ICR High phenotype. Similarly, when the NES are negative for TRs then, these TRs have high positive activity in ICR Low samples and high negative activity in ICR High samples. Thus, TRs with negative NES are more specific to the ICR Low phenotype (p-value < 1e-5). The bottom figure corresponds to subnetwork of MRs for 'BRCA'. The 'green' colored MRs are specific to ICR Low and 'yellow' colored MRs are specific to ICR High phenotype. The presence of communities of MRs and their corresponding target genes distinct to ICR High and ICR Low phenotype respectively can be observed.

Table S3. Top MRs which are common across all the ICR Enabled cancers are ranked based on fold change (FC) between average of median activity in ICR High vs average of median activity in ICR Low samples for all the ICR Enabled cancers using Wilcoxon ranksum test. We use FDR correction to obtain Padjust using the Benjamini & Hochberg method.

MR	P-value	Padjust	Mean1	Mean2	FC_Mean
SLA2	0.00094	0.00101	0.099	-0.076	0.175
PYHIN1	0.00094	0.00101	0.091	-0.078	0.169
SP140	0.00094	0.00101	0.084	-0.074	0.158
CD3D	0.00094	0.00101	0.081	-0.073	0.154
FASLG	0.00094	0.00101	0.083	-0.069	0.152
TBX21	0.00094	0.00101	0.083	-0.066	0.148
CD86	0.00094	0.00101	0.08	-0.067	0.146
IKZF1	0.00094	0.00101	0.073	-0.07	0.143
POU2AF1	0.00094	0.00101	0.075	-0.067	0.142
ZNF831	0.00094	0.00101	0.072	-0.069	0.141
LILRB4	0.00094	0.00101	0.073	-0.067	0.141
NCF1	0.00094	0.00101	0.077	-0.063	0.14
BTK	0.00094	0.00101	0.072	-0.068	0.14
CD28	0.00094	0.00101	0.07	-0.068	0.138
ITGB2	0.00094	0.00101	0.068	-0.068	0.136
MNDA	0.00094	0.00101	0.069	-0.065	0.134
PLEK	0.00094	0.00101	0.072	-0.062	0.134
CD40LG	0.00094	0.00101	0.068	-0.066	0.133
PIM2	0.00094	0.00101	0.074	-0.058	0.132
HAVCR2	0.00094	0.00101	0.069	-0.063	0.132
SPI1	0.00094	0.00101	0.066	-0.066	0.131
CXCL10	0.00094	0.00101	0.072	-0.052	0.124
TFEC	0.00094	0.00101	0.062	-0.062	0.124
IRF1	0.00094	0.00101	0.068	-0.055	0.123
CD74	0.00094	0.00101	0.059	-0.06	0.119
NLRC3	0.00094	0.00101	0.062	-0.056	0.119
TNFSF8	0.00094	0.00101	0.062	-0.056	0.118
CD4	0.00094	0.00101	0.054	-0.062	0.116
PSME2	0.00094	0.00101	0.069	-0.046	0.115
PSMB8	0.00094	0.00101	0.065	-0.049	0.114
LGALS9	0.00094	0.00101	0.065	-0.049	0.114
PSMB9	0.00094	0.00101	0.062	-0.051	0.113
CIITA	0.00094	0.00101	0.057	-0.054	0.111
BATF2	0.00094	0.00101	0.067	-0.042	0.109
LYL1	0.00094	0.00101	0.052	-0.057	0.109
PRKCB	0.00094	0.00101	0.053	-0.054	0.107
ETV7	0.00094	0.00101	0.067	-0.035	0.102
STAT1	0.00195	0.00195	0.058	-0.044	0.101
NLRP3	0.00094	0.00101	0.045	-0.05	0.096
TRIM22	0.00094	0.00101	0.04	-0.052	0.092
SP100	0.00094	0.00101	0.051	-0.041	0.091
DDX58	0.00136	0.00142	0.05	-0.041	0.091
FLI1	0.00094	0.00101	0.037	-0.045	0.082
PARP9	0.00195	0.00195	0.038	-0.032	0.07

Table S4. Top MRs which are common across all the ICR Disabled cancers are ranked based on fold change (FC) between average of median activity in ICR High vs average of median activity in ICR Low samples for all the ICR Disabled cancers using Wilcoxon ranksum test. We use FDR correction to obtain Padjust using the Benjamini & Hochberg method.

MRs	P-value	Padjust	Mean1	Mean2	FC_Mean
CD74	0.03038	0.03671	0.156	-0.103	0.259
CIITA	0.03038	0.03671	0.136	-0.073	0.208
NLRC5	0.03038	0.03671	0.126	-0.081	0.206
NCF1	0.03038	0.03671	0.124	-0.082	0.206
STAT1	0.03038	0.03671	0.119	-0.074	0.194
CXCL10	0.03038	0.03671	0.12	-0.074	0.194
PSMB9	0.03038	0.03671	0.12	-0.068	0.188
IKZF1	0.03038	0.03671	0.113	-0.071	0.184
NFAM1	0.03038	0.03671	0.114	-0.07	0.184
TRIM22	0.03038	0.03671	0.105	-0.077	0.183
PARP9	0.03038	0.03671	0.116	-0.063	0.179
TFEC	0.03038	0.03671	0.117	-0.062	0.179
PSMB8	0.03038	0.03671	0.111	-0.065	0.176
IRF1	0.0606	0.06277	0.112	-0.062	0.174
KLHL6	0.03038	0.03671	0.103	-0.071	0.174
PARP14	0.03038	0.03671	0.108	-0.059	0.167
CD28	0.03038	0.03671	0.085	-0.081	0.166
CD4	0.03038	0.03671	0.099	-0.063	0.163
PTAFR	0.03038	0.03671	0.1	-0.061	0.161
PARP15	0.03038	0.03671	0.099	-0.06	0.159
LILRB4	0.03038	0.03671	0.087	-0.06	0.146
PSME2	0.0606	0.06277	0.091	-0.052	0.143
CD38	0.0606	0.06277	0.081	-0.061	0.142
LGALS9	0.0606	0.06277	0.093	-0.048	0.141
FLI1	0.03038	0.03671	0.09	-0.047	0.138
NLRP3	0.03038	0.03671	0.075	-0.046	0.121
TMIGD3	0.03038	0.03671	0.062	-0.055	0.117
S100A8	0.03038	0.03671	0.035	-0.044	0.079
S1PR1	0.31232	0.31232	0.044	-0.027	0.07

Table S5. List of 118 MRs common to at least 4 out of 8 ICR Enabled Cancers (Selection Probability p=0.5). We showcase the cancers for which these MRs are differentially active.

MR	List of ICR Cancers	No. ICR Cancers
CD4	BLCA BRCA HNSC LIHC SARC SKCM STAD UCEC	8
ITGB2	BLCA BRCA HNSC LIHC SARC SKCM STAD UCEC	8
CD86	BLCA BRCA HNSC LIHC SARC SKCM STAD UCEC	8
PLEK	BLCA BRCA HNSC LIHC SARC SKCM STAD UCEC	8
NLRP3	BLCA BRCA HNSC LIHC SARC SKCM STAD UCEC	8
SPI1	BLCA BRCA HNSC LIHC SARC SKCM STAD UCEC	8
BTK	BLCA BRCA HNSC LIHC SARC SKCM STAD UCEC	8
TRIM22	BLCA BRCA HNSC LIHC SARC SKCM STAD UCEC	8
IKZF1	BLCA BRCA HNSC LIHC SARC SKCM STAD UCEC	8
NCF1	BLCA BRCA HNSC LIHC SARC SKCM STAD UCEC	8
MNDA	BLCA BRCA HNSC LIHC SARC SKCM STAD UCEC	8
FLI1	BLCA BRCA HNSC LIHC SARC SKCM STAD UCEC	8
HAVCR2	BLCA BRCA HNSC LIHC SARC SKCM STAD UCEC	8
CD28	BLCA BRCA HNSC LIHC SARC SKCM STAD UCEC	8
LGALS9	BLCA BRCA HNSC LIHC SARC SKCM STAD UCEC	8
LILRB4	BLCA BRCA HNSC LIHC SARC SKCM STAD UCEC	8
TFEC	BLCA BRCA HNSC LIHC SARC SKCM STAD UCEC	8
NLRC3	BLCA BRCA HNSC LIHC SARC SKCM STAD UCEC	8
SLA2	BLCA BRCA HNSC LIHC SARC SKCM STAD UCEC	8
CD3D	BLCA BRCA HNSC LIHC SARC SKCM STAD UCEC	8
IRF1	BLCA BRCA HNSC LIHC SARC SKCM STAD UCEC	8
PYHIN1	BLCA BRCA HNSC LIHC SARC SKCM STAD UCEC	8
ZNF831	BLCA BRCA HNSC LIHC SARC SKCM STAD UCEC	8
PSME2	BLCA BRCA HNSC LIHC SARC SKCM STAD UCEC	8
PSMB9	BLCA BRCA HNSC LIHC SARC SKCM STAD UCEC	8
PSMB8	BLCA BRCA HNSC LIHC SARC SKCM STAD UCEC	8
STAT1	BLCA BRCA HNSC LIHC SARC SKCM STAD UCEC	8
CIITA	BLCA BRCA HNSC LIHC SARC SKCM STAD UCEC	8
SP140	BLCA BRCA HNSC LIHC SARC SKCM STAD UCEC	8
TBX21	BLCA BRCA HNSC LIHC SARC SKCM STAD UCEC	8
BATF2	BLCA BRCA HNSC LIHC SARC SKCM STAD UCEC	8
POU2AF1	BLCA BRCA HNSC LIHC SARC SKCM STAD UCEC	8
CD40LG	BLCA BRCA HNSC LIHC SARC SKCM STAD UCEC	8
ETV7	BLCA BRCA HNSC LIHC SARC SKCM STAD UCEC	8
CD74	BLCA BRCA HNSC LIHC SARC SKCM STAD UCEC	8
FASLG	BLCA BRCA HNSC LIHC SARC SKCM STAD UCEC	8
PARP9	BLCA BRCA HNSC LIHC SARC SKCM STAD UCEC	8
DDX58	BLCA BRCA HNSC LIHC SARC SKCM STAD UCEC	8
CXCL10	BLCA BRCA HNSC LIHC SARC SKCM STAD UCEC	8
PIM2	BLCA BRCA HNSC LIHC SARC SKCM STAD UCEC	8
TNFSF8	BLCA BRCA HNSC LIHC SARC SKCM STAD UCEC	8
LYL1	BLCA BRCA HNSC LIHC SARC SKCM STAD UCEC	8
SP100	BLCA BRCA HNSC LIHC SARC SKCM STAD UCEC	8
PRKCB	BLCA BRCA HNSC LIHC SARC SKCM STAD UCEC	8
IL16	BLCA BRCA HNSC LIHC SARC STAD UCEC	7
RHOH	BLCA HNSC LIHC SARC SKCM STAD UCEC	7
ZBED2	BLCA BRCA LIHC SARC SKCM STAD UCEC	7
CARD16	BLCA BRCA HNSC LIHC SARC STAD UCEC	7
NLRC5	BLCA BRCA HNSC LIHC SARC SKCM STAD	7
RELB	BLCA BRCA HNSC LIHC SKCM STAD UCEC	7
PARP14	BLCA BRCA HNSC SARC SKCM STAD UCEC	7
AKNA	BLCA BRCA HNSC LIHC SKCM STAD UCEC	7
FOXP3	BLCA BRCA HNSC SARC SKCM STAD UCEC	7
PSMB10	BLCA HNSC LIHC SARC SKCM STAD UCEC	7
APOE	BLCA BRCA HNSC SARC STAD UCEC	6
TMIGD3	BLCA BRCA LIHC SKCM STAD UCEC	6
TRIM21	BLCA HNSC LIHC SARC SKCM STAD	6

Continued on next page

Table S5 – *Continued from previous page*

MR	List of ICR Cancers	No. ICR Cancers
PLSCR1	BLCA BRCA HNSC LIHC SARC STAD	6
PARP10	BLCA HNSC SARC SKCM STAD UCEC	6
DDX39B	BLCA BRCA LIHC SARC STAD UCEC	6
SALL2	BLCA BRCA HNSC SARC STAD UCEC	6
NMI	BRCA HNSC SARC SKCM STAD UCEC	6
ZEB2	BLCA BRCA HNSC LIHC UCEC	5
TGFB1I1	BLCA BRCA HNSC SARC STAD	5
SP110	BLCA HNSC SKCM STAD UCEC	5
SLC11A1	BLCA BRCA SARC SKCM UCEC	5
DTX3L	BLCA SARC SKCM STAD UCEC	5
OSM	BLCA LIHC SARC SKCM STAD	5
EIF2AK2	BLCA HNSC LIHC SKCM STAD	5
HSF4	BLCA BRCA LIHC SARC STAD	5
SS18L1	BLCA HNSC LIHC SARC STAD	5
ZNF789	BLCA BRCA LIHC SARC STAD	5
OVOL1	BLCA BRCA LIHC STAD UCEC	5
RAI1	BLCA BRCA HNSC LIHC SARC	5
ZNF354B	BLCA BRCA LIHC SARC UCEC	5
TLR3	BLCA HNSC SARC SKCM STAD	5
HELZ2	BLCA HNSC SARC SKCM STAD	5
PTTG1	BLCA BRCA HNSC LIHC STAD	5
TEAD2	BRCA HNSC SARC STAD UCEC	5
IGF2	BRCA HNSC SARC STAD UCEC	5
RHOG	BRCA LIHC SARC STAD UCEC	5
PSME1	BRCA LIHC SARC SKCM STAD	5
ACTN1	BLCA HNSC SARC STAD	4
MSC	BLCA BRCA STAD UCEC	4
CDC45	BLCA BRCA LIHC STAD	4
AURKB	BLCA BRCA LIHC STAD	4
S100A8	BLCA BRCA LIHC SARC	4
PABPC1L	BLCA BRCA STAD UCEC	4
TRIM52	BLCA BRCA HNSC LIHC	4
ZNF169	BLCA BRCA LIHC SARC	4
ZNF337	BLCA BRCA LIHC SARC	4
DMTF1	BLCA BRCA LIHC SARC	4
NR2C2	BLCA BRCA LIHC SARC	4
CDH1	BLCA HNSC LIHC STAD	4
TBX2	BLCA BRCA HNSC STAD	4
JUP	BLCA HNSC LIHC STAD	4
ZNF805	BLCA BRCA LIHC SARC	4
DLGAP5	BLCA BRCA LIHC STAD	4
S100A9	BLCA BRCA LIHC SARC	4
RRM2	BLCA BRCA LIHC STAD	4
CREB3L4	BLCA BRCA HNSC STAD	4
HEXIM2	BLCA BRCA LIHC SARC	4
DAB2	BLCA HNSC SARC UCEC	4
ZNF7	BLCA HNSC SKCM UCEC	4
BIRC5	BLCA BRCA LIHC STAD	4
SMARCC2	BLCA BRCA LIHC SARC	4
ZSCAN32	BLCA BRCA LIHC SKCM	4
RPS14	BLCA HNSC LIHC STAD	4
ZNF423	BLCA BRCA LIHC STAD	4
APBB3	BRCA HNSC LIHC SARC	4
ZNF770	BRCA HNSC LIHC SARC	4
PYGO1	BRCA HNSC SARC STAD	4
TCEAL3	BRCA HNSC SARC STAD	4
ARL2BP	BRCA HNSC LIHC SARC	4
IRF7	HNSC SARC SKCM STAD	4
SMO	HNSC SARC STAD UCEC	4

Continued on next page

Table S5 – *Continued from previous page*

MR	List of ICR Cancers	No. ICR Cancers
PYCARD	HNSC LIHC SARC STAD	4
TAF2	HNSC SKCM STAD UCEC	4

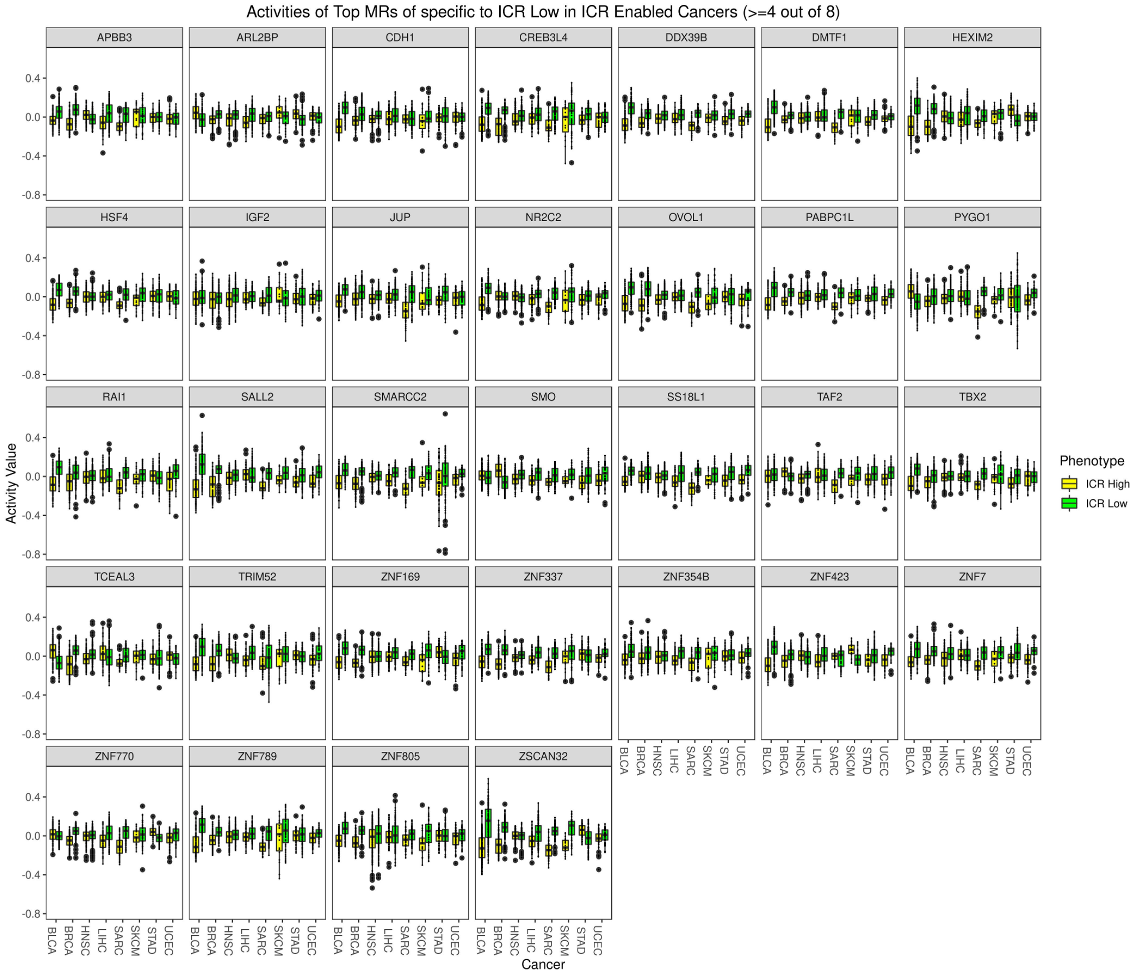

Fig. S4. Boxplots comparing 32 common MR activities (MRs in at least 4 out of 8 ICR Enabled cancers but are TRs with regulon size $\geq$ 10 target genes in all 8 cancers) between the ICR High and the ICR Low samples for each of the 8 ICR Enabled cancer types. Predominantly, every one of these MRs have high activity in ICR Low samples and low activity in ICR High samples for at least 4 out of 8 cancer tissues (Difference between mean activities in ICR High samples and the mean of the activities in ICR Low samples across the 8 ICR Enabled cancers is < 0 and is statistically significant w.r.t. Wilcoxon ranksum test i.e. FDR Adjusted P-value < 0.05). Thus, these MRs are considered to be specific to ICR Low phenotype.

Table S6. List of 117 significant MRs for ICR Enabled cancers and are ranked based on fold change (FC) between activities in ICR High vs activities in ICR Low samples across all the 8 ICR Enabled cancers using Wilcoxon ranksum test.

MR	FC_Median	FDR Adjusted P-value
SLA2	0.164	1.89040617212528e-194
PYHIN1	0.155	2.61369286749188e-211
SP140	0.154	7.72354672556269e-161
CD3D	0.149	1.59276686004567e-183
TBX21	0.148	1.80301432383196e-199
FASLG	0.146	2.37610723264157e-202
FOXP3	0.146	4.69994481936225e-151
BTK	0.141	2.3063834753307e-161
CD86	0.138	2.17094654014363e-151
LILRB4	0.138	5.20884445559781e-155
ZNF831	0.138	1.51856054458993e-145
MNDA	0.134	4.78869345282125e-135
CD40LG	0.133	2.69199480491459e-149
IKZF1	0.132	1.01544714727255e-119
SPI1	0.132	1.86664548677196e-134
TFEC	0.132	5.92088231191292e-141
NCF1	0.13	1.0320805719677e-159
PIM2	0.13	1.51856054458993e-145
CD28	0.127	7.20460436051533e-116
PLEK	0.126	2.95072787077671e-136
CXCL10	0.125	2.63609869573832e-143
ITGB2	0.125	5.08383701871894e-128
POU2AF1	0.124	6.27037214856681e-124
TRIM21	0.123	2.34015407046057e-125
ZBED2	0.12	7.75951328272334e-86
IRF1	0.119	1.4934368185186e-165
HAVCR2	0.118	6.94773914083119e-150
RHOG	0.112	3.86993883777646e-116
TNFSF8	0.112	2.47180877806302e-101
APOE	0.11	4.59828733786067e-76
CIITA	0.109	1.98560569909881e-126
NLRC3	0.109	1.01074214280633e-119
CARD16	0.108	2.19697614049764e-123
CD4	0.108	1.69813591703916e-95
CD74	0.108	8.22435926866056e-120
S100A8	0.108	1.77378067235811e-99
PLSCR1	0.107	1.41547137676764e-95
PSMB8	0.107	7.20460436051533e-116
PSME2	0.107	4.24112682584836e-110
NLRC5	0.106	7.46850582440936e-128
PSMB10	0.106	5.08383701871894e-128
S100A9	0.106	1.83271079199434e-98
PRKCB	0.105	7.4783918308964e-92
PSMB9	0.105	2.51637660437774e-130
STAT1	0.105	2.31101622679454e-104
LGALS9	0.103	2.80834182117842e-105
TMIGD3	0.103	4.56663032545212e-78
BATF2	0.1	1.83592766365221e-91
IRF7	0.1	1.60902555234146e-57
LYL1	0.098	1.14511190917552e-101
NMI	0.098	9.46588115689498e-56
ETV7	0.097	2.42999062258095e-103
MSC	0.097	1.18447410942323e-74
RHOH	0.097	1.18447410942323e-74
SP110	0.096	1.81181989456572e-40
TRIM22	0.094	1.83592766365221e-91
OSM	0.093	3.26435458802796e-82

Continued on next page

Table S6 – *Continued from previous page*

MR	FC_Median	FDR Adjusted P-value
RELB	0.093	2.0939673447732e-86
IL16	0.092	9.12639303572681e-74
SP100	0.091	3.6005298600059e-78
DDX58	0.09	5.62473692018708e-77
PARP14	0.089	6.6468632429981e-104
NLRP3	0.085	2.51405909670267e-67
AKNA	0.084	2.86219111266103e-61
PTTG1	0.084	2.86219111266103e-61
FLI1	0.075	3.35775588314465e-58
RRM2	0.074	2.87737983469095e-37
PYCARD	0.071	3.24231724203874e-41
TLR3	0.07	1.00448921246194e-56
DAB2	0.069	1.35110038927383e-30
PARP10	0.068	5.60108908072613e-45
SLC11A1	0.068	4.23233817120897e-57
AURKB	0.067	1.09448737292422e-38
HELZ2	0.066	5.17348210146846e-43
DLGAP5	0.065	4.94024450392205e-38
ZEB2	0.064	4.8696028057076e-44
DTX3L	0.062	2.00325992290363e-52
PARP9	0.06	7.05578572419513e-57
PSME1	0.056	6.76052564515741e-34
ACTN1	0.046	2.04474638823371e-14
BIRC5	0.045	6.44099492654131e-24
EIF2AK2	0.045	6.90937432410871e-22
CDC45	0.044	6.5996624475803e-19
TEAD2	0.029	3.19232299610979e-06
TGFB1I1	0.011	0.00405585056315986
ARL2BP	-0.014	0.000686184812979057
SMO	-0.02	0.00275919269295844
PYGO1	-0.023	4.49123529445471e-05
TCEAL3	-0.023	4.48261430357215e-06
TAF2	-0.025	2.71500245594137e-09
IGF2	-0.029	2.33514705416091e-08
ZNF770	-0.034	1.6649651960007e-19
NR2C2	-0.036	2.21013113580498e-23
ZNF423	-0.045	6.48324293079246e-19
ZNF354B	-0.049	3.55117783571639e-30
APBB3	-0.05	6.76425544656246e-30
DMTF1	-0.054	1.2278758284415e-45
TBX2	-0.054	4.90362112861681e-39
CDH1	-0.055	3.02642420400856e-38
TRIM52	-0.059	1.83482840233635e-31
HSF4	-0.063	9.1576405209831e-45
HEXIM2	-0.066	1.80233526534487e-29
RAI1	-0.066	1.59161799881158e-38
JUP	-0.067	9.3239888879074e-43
SS18L1	-0.068	2.15535816713701e-48
PABPC1L	-0.073	2.72214254443875e-62
ZNF169	-0.074	2.81941247017186e-44
ZNF805	-0.074	1.03305643666662e-38
ZNF789	-0.075	2.26727077148448e-52
ZNF337	-0.077	5.17976367637602e-73
ZNF7	-0.077	5.82195823104261e-57
ZSCAN32	-0.08	2.06864900352898e-60
SMARCC2	-0.084	6.22384673752519e-66
DDX39B	-0.085	2.22913469839856e-81
OVOL1	-0.088	5.44231150931805e-66
CREB3L4	-0.098	4.16208049708269e-63

Continued on next page

Table S6 – *Continued from previous page*

MR	FC_Median	FDR Adjusted P-value
SALL2	-0.105	4.66566885305639e-69

C. Consensus MRs Specific to ICR-H and ICR-L Phenotypes. A total of 234 MRs were obtained and their corresponding cancer subtypes were described in Supp. Table S7. Moreover, we identified that 214 of these 234 MRs had differential activity (FDR-adjusted p-values ≤ 0.05 using Wilcoxon test) between the ICR-H and ICR-L samples of the 4 ICR-D cancers taken together (see Supp. Table S8). 84 of these MRs had high median activity in ICR-L samples and low median activity in ICR-H samples in at least 2 out of the 4 ICR-D cancers (see Supp. Table S8 for significance). These 84 MRs were considered to be specific to the ICR-L phenotype for the 4 ICR-D cancers. The activity profiles of these 84 MRs for each of the 4 ICR-D cancers were illustrated in Supp. Fig S5.

Table S7. List of 234 MRs common to at least 2 out of 4 ICR Disabled Cancers (Selection Probability p=0.5). We also showcase the cancers for which these MRs are differentially active.

MR	List of ICR Cancers	No. ICR Cancers
CIITA	LGG KIRC PAAD UVM	4
TFEC	LGG KIRC PAAD UVM	4
CD4	LGG KIRC PAAD UVM	4
PARP9	LGG KIRC PAAD UVM	4
IKZF1	LGG KIRC PAAD UVM	4
LILRB4	LGG KIRC PAAD UVM	4
HCLS1	LGG KIRC PAAD UVM	4
PTAFR	LGG KIRC PAAD UVM	4
CD74	LGG KIRC PAAD UVM	4
NCF1	LGG KIRC PAAD UVM	4
LGALS9	LGG KIRC PAAD UVM	4
NLRP3	LGG KIRC PAAD UVM	4
PARP14	LGG KIRC PAAD UVM	4
IRF1	LGG KIRC PAAD UVM	4
CD28	LGG KIRC PAAD UVM	4
TMIGD3	LGG KIRC PAAD UVM	4
IKZF3	LGG KIRC PAAD UVM	4
TRIM22	LGG KIRC PAAD UVM	4
STAT1	LGG KIRC PAAD UVM	4
KLHL6	LGG KIRC PAAD UVM	4
PSMB9	LGG KIRC PAAD UVM	4
CXCL10	LGG KIRC PAAD UVM	4
FLI1	LGG KIRC PAAD UVM	4
NFAM1	LGG KIRC PAAD UVM	4
S100A8	LGG KIRC PAAD UVM	4
PSMB8	LGG KIRC PAAD UVM	4
NLRC5	LGG KIRC PAAD UVM	4
PARP15	LGG KIRC PAAD UVM	4
S1PR1	LGG KIRC PAAD UVM	4
CD38	LGG KIRC PAAD UVM	4
BCL11B	LGG KIRC PAAD UVM	4
ZBTB47	LGG KIRC PAAD UVM	4
PSME2	LGG KIRC PAAD UVM	4
CARD16	LGG KIRC UVM	3
TRIM21	LGG KIRC UVM	3
ENG	LGG KIRC PAAD	3
HAVCR2	LGG PAAD UVM	3
NLRC4	LGG KIRC PAAD	3
BATF	LGG KIRC UVM	3
HTATIP2	LGG PAAD UVM	3
BCL6B	LGG KIRC PAAD	3
PRKCH	LGG KIRC PAAD	3
MECOM	LGG KIRC UVM	3
HMOX1	LGG KIRC UVM	3
DTX3L	LGG PAAD UVM	3

Continued on next page

Table S7 – *Continued from previous page*

MR	List of ICR Cancers	No. ICR Cancers
TNFRSF4	LGG PAAD UVM	3
HAMP	LGG KIRC PAAD	3
TLR2	LGG KIRC PAAD	3
ICAM1	LGG KIRC PAAD	3
S100A9	LGG PAAD UVM	3
CEBPA	LGG KIRC UVM	3
LEF1	LGG PAAD UVM	3
TSSK4	LGG KIRC PAAD	3
HDAC10	LGG KIRC PAAD	3
TCERG1	LGG KIRC PAAD	3
HDAC11	LGG KIRC UVM	3
PDE2A	LGG KIRC PAAD	3
PRKCB	LGG KIRC PAAD	3
RHOG	LGG KIRC PAAD	3
ETV7	LGG KIRC UVM	3
FABP4	LGG KIRC PAAD	3
TMSB4X	LGG KIRC UVM	3
EDNRB	LGG KIRC PAAD	3
BCL3	LGG KIRC UVM	3
STAT2	LGG KIRC UVM	3
CCNT2	LGG PAAD UVM	3
TMEM100	LGG KIRC PAAD	3
APOE	LGG KIRC PAAD	3
KLF9	LGG KIRC PAAD	3
SMO	LGG KIRC PAAD	3
MSC	LGG KIRC PAAD	3
PRDM1	KIRC PAAD UVM	3
LPXN	KIRC PAAD UVM	3
ZNF831	KIRC PAAD UVM	3
CMKLR1	KIRC PAAD UVM	3
PIM2	KIRC PAAD UVM	3
PSMB10	KIRC PAAD UVM	3
BATF2	KIRC PAAD UVM	3
POU2AF1	KIRC PAAD UVM	3
STAT4	KIRC PAAD UVM	3
NOTCH3	KIRC PAAD UVM	3
CAVIN1	LGG PAAD	2
TGFB2	LGG KIRC	2
PLSCR1	LGG PAAD	2
SP140L	LGG UVM	2
CAV1	LGG PAAD	2
RPS6KA1	LGG KIRC	2
DCN	LGG PAAD	2
TLR6	LGG KIRC	2
PYCARD	LGG KIRC	2
ASF1B	LGG KIRC	2
DEPDC1	LGG KIRC	2
MYBL2	LGG KIRC	2
LUM	LGG PAAD	2
FOXS1	LGG UVM	2
CCNA2	LGG KIRC	2
CENPF	LGG KIRC	2
FOXM1	LGG KIRC	2
NOTCH4	LGG KIRC	2
DLGAP5	LGG KIRC	2
DLL4	LGG UVM	2
COL1A1	LGG PAAD	2
TLR3	LGG UVM	2
CYP1B1	LGG PAAD	2

Continued on next page

Table S7 – *Continued from previous page*

MR	List of ICR Cancers	No. ICR Cancers
SERPINE1	LGG PAAD	2
PLK3	LGG PAAD	2
DAB2	LGG PAAD	2
LMO2	LGG UVM	2
CDC6	LGG KIRC	2
RPS6KA4	LGG KIRC	2
SP110	LGG PAAD	2
VGLL3	LGG PAAD	2
ING5	LGG KIRC	2
S100A1	LGG KIRC	2
NRIP2	LGG KIRC	2
TRIM52	LGG PAAD	2
HSF4	LGG PAAD	2
SFRP2	LGG PAAD	2
ZEB2	LGG PAAD	2
STK3	LGG KIRC	2
MTERF2	LGG PAAD	2
ZNF337	LGG PAAD	2
BTRC	LGG UVM	2
L3MBTL1	LGG PAAD	2
GTF2H4	LGG PAAD	2
APBB1	LGG KIRC	2
HLF	LGG KIRC	2
KAT2A	LGG PAAD	2
ZNF133	LGG PAAD	2
RNF141	LGG KIRC	2
SOX6	LGG KIRC	2
ZNF536	LGG PAAD	2
ZNF276	LGG KIRC	2
LHX6	LGG PAAD	2
CRY2	LGG KIRC	2
MAPRE3	LGG KIRC	2
MICAL2	LGG UVM	2
BCL2L12	LGG UVM	2
PRKCZ	LGG KIRC	2
MAML3	LGG KIRC	2
ZNF354B	LGG PAAD	2
E2F1	LGG KIRC	2
CDH13	LGG KIRC	2
PDPK1	LGG KIRC	2
IRF3	LGG KIRC	2
OGT	LGG KIRC	2
CEBPB	LGG PAAD	2
KLF15	LGG KIRC	2
PLK1	LGG KIRC	2
IFI16	LGG PAAD	2
UBE2V1	LGG PAAD	2
PTTG1	LGG KIRC	2
GLI4	LGG PAAD	2
FBXL15	LGG PAAD	2
NFKB2	LGG KIRC	2
PFKM	LGG KIRC	2
NMI	LGG UVM	2
ZGPAT	LGG PAAD	2
LHX4	LGG PAAD	2
EMX2	LGG KIRC	2
AR	LGG PAAD	2
ZNF169	LGG PAAD	2
PRMT5	LGG KIRC	2

Continued on next page

Table S7 – *Continued from previous page*

MR	List of ICR Cancers	No. ICR Cancers
MAFF	LGG KIRC	2
NUFIP1	LGG PAAD	2
ELF3	LGG PAAD	2
PARP10	LGG UVM	2
SETD5	LGG UVM	2
BOLA1	LGG PAAD	2
PIDD1	LGG PAAD	2
IPPK	LGG KIRC	2
HEY1	LGG KIRC	2
ORC1	LGG KIRC	2
PHF11	LGG KIRC	2
PSME1	LGG UVM	2
KMT5C	LGG PAAD	2
PHB	LGG KIRC	2
TRIP13	LGG UVM	2
GATAD1	LGG PAAD	2
NFKBIL1	LGG UVM	2
E2F7	LGG KIRC	2
ELF1	LGG KIRC	2
ZNF770	LGG KIRC	2
HIRA	LGG PAAD	2
PCBP3	LGG PAAD	2
GRHL3	LGG KIRC	2
MEIS3	LGG KIRC	2
ZNF408	LGG UVM	2
PINK1	LGG KIRC	2
SAP18	LGG PAAD	2
KAT5	LGG KIRC	2
ZNF852	LGG KIRC	2
SIRT5	LGG KIRC	2
IRF9	KIRC UVM	2
PABPC1L	KIRC PAAD	2
CDK5RAP3	KIRC PAAD	2
GAS7	KIRC PAAD	2
AKNA	KIRC PAAD	2
ADAM8	KIRC UVM	2
PEG3	KIRC UVM	2
RUNX1T1	KIRC UVM	2
PRDM16	KIRC UVM	2
ZNF366	KIRC PAAD	2
SOX17	KIRC PAAD	2
ZNF423	KIRC PAAD	2
ZNF619	KIRC UVM	2
EPAS1	KIRC PAAD	2
PLPP3	KIRC PAAD	2
PPM1A	KIRC PAAD	2
TEAD2	KIRC UVM	2
NTRK3	KIRC PAAD	2
BMP6	KIRC PAAD	2
SETD3	KIRC PAAD	2
ESRRG	KIRC UVM	2
ZNF214	KIRC PAAD	2
MXD3	KIRC PAAD	2
IKBKG	KIRC UVM	2
ALKBH4	KIRC PAAD	2
ZNF696	KIRC PAAD	2
KANK2	KIRC PAAD	2
ZMYND15	KIRC UVM	2
ZNF3	KIRC PAAD	2

Continued on next page

Table S7 – *Continued from previous page*

MR	List of ICR Cancers	No. ICR Cancers
EIF2AK2	KIRC PAAD	2
KLF13	KIRC PAAD	2
RCOR1	KIRC PAAD	2
TGFBR3	KIRC PAAD	2
ZNF91	KIRC PAAD	2
SIRT7	KIRC PAAD	2
ZNF581	KIRC UVM	2
THOC1	PAAD UVM	2
EFCAB6	PAAD UVM	2
ZNF19	PAAD UVM	2
ZNF7	PAAD UVM	2
COMMD5	PAAD UVM	2

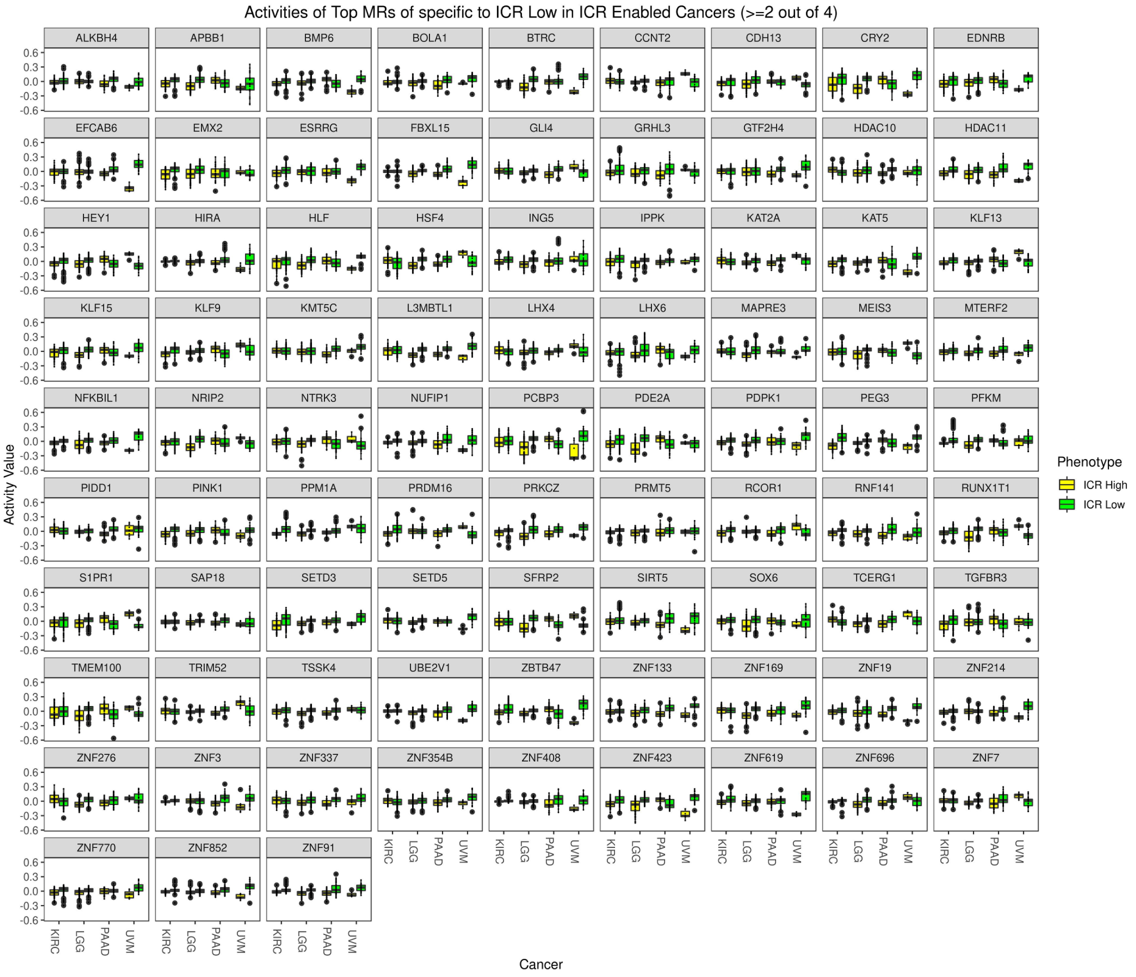

Fig. S5. Boxplots comparing 84 common MR activities (MRs in at least 2 out of 4 ICR Disabled cancers but are TRs with regulon size $\geq$ 10 target genes in all 4 cancers) between the ICR High and the ICR Low samples for each of the 4 ICR Enabled cancer types. Predominantly, every one of these MRs have high activity in ICR Low samples and low activity in ICR High samples for at least 2 out of 4 cancer tissues (Difference between mean of the activities in ICR High samples and the mean of the activities in ICR Low samples across the 4 ICR Disabled cancers is < 0 and is statistically significant w.r.t. Wilcoxon ranksum test for FDR Adjusted P-value < 0.05). Thus, these MRs are considered to be specific to ICR Low phenotype.

Table S8. List of 214 MRs for ICR Disabled cancers and are ranked based on fold change (FC) between activities in ICR High vs activities in ICR Low samples across all 4 ICR Disabled cancers using Wilcoxon ranksum test.

MR	FC_Median	FDR Adjusted P-value
CD74	0.213	9.68474806910364e-83
IKZF3	0.206	7.08593139899144e-87
BATF	0.203	1.41888326418268e-68
PSMB9	0.201	4.88489726347782e-78
CIITA	0.192	8.88042257589214e-73
CXCL10	0.182	7.08593139899144e-87
IRF1	0.182	5.55059061069545e-74
NCF1	0.182	1.07785556817273e-70
CD28	0.174	1.94636088539621e-70
STAT1	0.17	2.21921102089951e-82
PARP9	0.165	9.34317167093825e-75
CARD16	0.164	5.42942191023182e-63
PSMB8	0.162	2.01739589934513e-63
NFAM1	0.16	5.54623425938461e-60
NLRC5	0.158	1.48105685966635e-64
LUM	0.155	1.02284638401389e-32
PARP14	0.155	3.05728913326844e-73
LILRB4	0.152	6.46833664532087e-47
IFI16	0.151	3.89939044888252e-53
LPXN	0.151	1.14901922057592e-49
TLR2	0.15	2.40968399882976e-49
IKZF1	0.149	2.29270397018973e-55
CD4	0.148	2.55624857852132e-57
ETV7	0.147	3.72556421158327e-51
HCLS1	0.146	1.20543035039144e-56
SP140L	0.146	2.45267065051384e-47
COL1A1	0.144	1.32451646855468e-36
TRIM22	0.14	5.78328349626378e-64
PARP15	0.138	5.42942191023182e-63
PSMB10	0.136	3.52773800356725e-48
TMSB4X	0.134	1.01402175543775e-60
TFEC	0.132	3.08243847551768e-36
LGALS9	0.13	1.02312785256106e-48
PLSCR1	0.128	1.7766951805347e-37
BATF2	0.127	6.94264489879132e-39
NLRC4	0.127	4.69177036127025e-53
HAMP	0.126	2.21991717129337e-47
PSME2	0.125	7.04345854763932e-46
HAVCR2	0.123	3.50278624780171e-40
KLHL6	0.122	1.93045311299686e-56
DTX3L	0.121	1.9373495515162e-43
ICAM1	0.121	2.68213055315834e-59
PTAFR	0.121	7.85350026907788e-44
ENG	0.118	8.0344866327019e-32
RPS6KA1	0.118	3.46326880933066e-42
ZNF831	0.116	1.78624541854703e-18
HMOX1	0.112	6.99339126612788e-51
TMIGD3	0.112	9.63511514168786e-35
DAB2	0.111	9.17388819625404e-19
TLR6	0.111	2.20526498967909e-37
PYCARD	0.108	1.23739770641236e-34
TRIM21	0.108	1.08954907738014e-46
BCL3	0.107	1.86271033931745e-38
NMI	0.107	3.29323503133125e-41
CEBPA	0.106	2.24999248167256e-36
LEF1	0.105	4.26366873011147e-20
PARP10	0.104	7.85934068398065e-31

Continued on next page

Table S8 – *Continued from previous page*

MR	FC_Median	FDR Adjusted P-value
SERPINE1	0.102	7.36893230305862e-33
PTTG1	0.099	2.39152503377946e-24
PLK3	0.098	1.02267927304868e-42
CYP1B1	0.092	2.80120362642034e-20
DLGAP5	0.091	1.87928036078006e-19
CENPF	0.09	1.96484399041241e-18
NFKB2	0.09	4.04528936914627e-31
RHOG	0.09	2.64727338011711e-41
S100A9	0.09	6.66389397104998e-39
CAV1	0.088	7.45386239026953e-20
DEPDC1	0.088	4.02465135208627e-22
MYBL2	0.088	5.52014991971673e-15
LMO2	0.087	4.22697316572041e-28
TLR3	0.086	1.67275454100381e-24
SP110	0.085	3.34935275522166e-47
ORC1	0.084	3.46654888070842e-20
AR	0.083	1.30901881929734e-07
CAVIN1	0.083	5.78793535907864e-23
STAT2	0.083	7.28609393896121e-35
CCNA2	0.082	2.73988487800561e-16
STAT4	0.082	6.06787119768509e-14
AKNA	0.081	2.2384189274597e-28
CD38	0.08	2.2250182351911e-18
FLI1	0.079	3.81177713015987e-37
NLRP3	0.077	1.14106231711567e-31
PRDM1	0.077	3.91008163217685e-40
CEBPB	0.076	9.68181978935758e-24
FOXS1	0.076	1.4869678383284e-21
IRF3	0.076	1.28981467473016e-17
PHF11	0.074	1.68271526558495e-19
PSME1	0.074	5.32637162684531e-28
POU2AF1	0.073	7.60838522566743e-24
DCN	0.072	1.20680425638735e-13
TGFB2	0.071	2.55085236211236e-18
ASF1B	0.07	1.86848593712007e-16
CDC6	0.07	3.14955904542947e-14
S100A8	0.07	3.11311178492008e-32
GAS7	0.068	7.69460393065619e-16
RPS6KA4	0.068	3.83225811654221e-17
KANK2	0.067	2.46527245747943e-07
E2F7	0.066	1.06180143259416e-18
EIF2AK2	0.065	1.17006191333791e-40
FOXM1	0.065	6.39149471674587e-12
ZMYND15	0.065	1.66527843287494e-14
ELF1	0.061	3.6963247626066e-20
HTATIP2	0.061	3.24187368927566e-10
TNFRSF4	0.061	2.80120362642034e-20
BCL2L12	0.059	1.92229711150875e-10
MSC	0.059	9.68785849557501e-12
PLK1	0.058	9.41350638685498e-09
STK3	0.054	3.18489731587445e-10
BCL11B	0.053	1.06903233538681e-07
PRKCB	0.051	0.000484422305479129
VGLL3	0.049	1.55796637291686e-09
PIM2	0.043	2.20440915325954e-13
PRKCH	0.04	1.39515126933686e-13
CMKLR1	0.039	4.43687091304703e-21
DLL4	0.038	1.53002906712539e-05
MECOM	0.037	2.91425656868576e-05

Continued on next page

Table S8 – *Continued from previous page*

MR	FC_Median	FDR Adjusted P-value
MXD3	0.033	5.76680724909852e-08
SMO	0.033	2.35757782316622e-08
TEAD2	0.033	0.000242563945669362
BCL6B	0.032	7.97771201695212e-05
ADAM8	0.031	9.09574166388002e-06
TRIP13	0.03	0.00135282714792755
SIRT7	0.027	1.04217530228197e-05
NOTCH3	0.026	1.70347515974279e-05
PABPC1L	0.026	3.04303827664506e-05
E2F1	0.022	0.001386313440597
IRF9	0.017	2.15510043018493e-05
APOE	0.015	0.00571384272494136
COMMD5	0.01	0.000307671134934315
NOTCH4	0.008	0.0356904260740047
TGFBR3	-0.016	0.0168702247380092
EFCAB6	-0.017	2.72484182935665e-05
CCNT2	-0.02	0.00265391248007965
HIRA	-0.02	7.45762941223468e-12
HDAC10	-0.022	0.0356904260740047
KMT5C	-0.022	0.00393775719006912
TRIM52	-0.022	0.00385750706095971
LHX4	-0.023	0.0428660120447634
PRDM16	-0.023	3.04303827664506e-05
ZNF408	-0.023	1.44964862890204e-10
RCOR1	-0.026	1.23275257056667e-08
ZNF214	-0.026	8.3299666041383e-07
ALKBH4	-0.027	2.0120005654107e-06
PIDD1	-0.028	0.00255761052131938
SAP18	-0.028	4.11036241386734e-07
ZNF3	-0.028	3.52745058283966e-06
ZNF354B	-0.028	0.012977797872973
ZNF7	-0.03	3.4621888366037e-07
KLF13	-0.031	3.30727312850569e-05
TCERG1	-0.031	4.84119872804587e-05
UBE2V1	-0.031	2.15045774082522e-14
KAT2A	-0.033	0.000860610794306152
SETD5	-0.033	2.85561675829626e-07
GLI4	-0.034	5.69106710891276e-07
GTF2H4	-0.034	0.00233347446610732
KLF9	-0.036	6.22247454377484e-07
MAPRE3	-0.037	3.58112826910196e-11
ZNF852	-0.038	1.08798995891484e-18
BTRC	-0.04	5.68123601795649e-29
MEIS3	-0.04	3.49877671858986e-06
NTRK3	-0.041	3.7635348595427e-07
BMP6	-0.042	1.2201105361839e-08
FBXL15	-0.042	2.08326395127088e-21
ZNF337	-0.042	3.05216076908658e-07
S1PR1	-0.043	0.00117842956696837
EDNRB	-0.044	1.07776578369266e-06
ESRRG	-0.044	4.26076724742221e-08
ZNF770	-0.044	9.54631059621371e-18
PRMT5	-0.045	1.06978282003934e-13
ZBTB47	-0.048	4.72428916592353e-13
BOLA1	-0.049	5.50806624945908e-14
CDH13	-0.049	6.66478293308555e-09
HEY1	-0.049	3.6298691219436e-06
NUFIP1	-0.049	6.3161010690972e-19
SOX6	-0.049	1.61712844475646e-09

Continued on next page

Table S8 – *Continued from previous page*

MR	FC_Median	FDR Adjusted P-value
ZNF91	-0.05	3.69120615430424e-34
IPPK	-0.054	6.98270781332347e-19
LHX6	-0.054	1.11337335510644e-06
ZNF619	-0.054	1.95060697347103e-16
KAT5	-0.056	6.35819662041635e-15
ZNF696	-0.057	2.11468990784246e-24
PFKM	-0.059	7.23636782827384e-24
TSSK4	-0.06	2.98415097108246e-18
ZNF276	-0.06	1.49417360860254e-07
SIRT5	-0.061	3.84284936200138e-15
MTERF2	-0.062	1.07858988334861e-17
PDPK1	-0.062	7.53358564410645e-21
ING5	-0.065	1.2485191218329e-18
NFKBIL1	-0.065	9.2144305908734e-23
GRHL3	-0.066	1.57296543051213e-08
PPM1A	-0.066	1.56114372241445e-32
ZNF133	-0.066	1.03583483707125e-16
ZNF19	-0.068	4.28292523023734e-15
PEG3	-0.069	1.31420278242008e-29
ZNF423	-0.069	4.85345469242188e-20
PINK1	-0.073	1.26876074671732e-18
RNF141	-0.076	2.6656853258007e-28
SETD3	-0.076	3.56218373651232e-22
SFRP2	-0.076	9.51809087863127e-11
RUNX1T1	-0.081	3.44833611972003e-18
HLF	-0.082	2.76898133798413e-17
NRIP2	-0.083	1.17513856966879e-16
HDAC11	-0.084	1.44201830264145e-31
HSF4	-0.085	1.73612816115137e-12
KLF15	-0.088	2.9604210619535e-18
PCBP3	-0.091	1.25086191214391e-14
APBB1	-0.093	1.6286150097753e-22
EMX2	-0.093	1.98415332648457e-13
TMEM100	-0.096	5.34650818356148e-08
ZNF169	-0.097	2.07720632122216e-25
L3MBTL1	-0.101	8.02333963853908e-29
PRKCZ	-0.108	3.35297344918055e-35
PDE2A	-0.109	4.15588365905479e-21
CRY2	-0.161	9.40376093126022e-27

Table S9. List of 162 MRs when performing analysis w.r.t. ICR High phenotype. Out of these 155 MRs have high median activity in ICR High samples (specific to ICR High) for the 12 cancers of interest and 7 MRs have high median activity in ICR Disabled cancers but low median activity in ICR Enabled cancers (highlighted in bold). The significance of difference in activities in ICR Enabled cancer samples vs ICR Disabled cancer samples is highlighted using the Wilcoxon ranksum test.

MR	FC_Median	Median_ICR_Enabled	Median_ICR_Disabled	FDR Adjusted P-value
SLA2	0.087	0.087	0	0.003711766062647
PYHIN1	0.087	0.087	0	4.02427828633242e-06
SP140	0.064	0.082	0.017	0.000343810680100754
TBX21	0.081	0.081	0	5.55324241923791e-14
FASLG	0.07	0.079	0.008	0.0356906956133615
FOXP3	0.054	0.079	0.025	4.95448464663692e-06
CD3D	0.078	0.078	0	1.03818379864573e-39
TLR6	0.003	0.075	0.071	0.811993775001751
LILRB4	-0.016	0.074	0.09	0.144903499024325
BTK	0.051	0.073	0.022	0.00075412340382534
CD86	0.072	0.072	0	1.7005030654895e-31
TRIM21	0.01	0.072	0.063	0.178984763974162
ZNF831	0.018	0.071	0.052	0.138637896839997
CXCL10	-0.035	0.071	0.106	5.57995735441302e-12
TFEC	-0.012	0.07	0.081	0.0939612578777315
NCF1	-0.033	0.07	0.103	7.60967036123198e-08
MNDA	0.069	0.069	0	5.68266611928883e-07
SPI1	0.069	0.069	0	2.44232585634666e-23
PIM2	0.047	0.069	0.022	6.45452401353632e-10
NLRC4	-0.004	0.069	0.073	0.316503484080799
STAT4	0.041	0.069	0.027	2.74397241227073e-05
PLEK	0.068	0.068	0	0.00113531762729646
ZBED2	0.068	0.068	0	4.79781006531967e-24
CD40LG	0.067	0.067	0	3.28559017742786e-10
IKZF1	-0.026	0.067	0.094	0.00349195911510396
CD28	-0.029	0.066	0.095	3.58874982672294e-06
ITGB2	0.057	0.065	0.008	0.00113531762729646
IRF1	-0.041	0.065	0.106	1.13613588096465e-10
ZMYND15	0.021	0.065	0.045	7.4014490677091e-05
POU2AF1	0.022	0.063	0.041	0.000939265857816592
HAVCR2	-0.008	0.063	0.071	0.24021935435976
PLSCR1	0.003	0.062	0.059	0.25813386980644
PRDM1	0.015	0.061	0.047	0.0258254152395049
RHOG	0.01	0.06	0.05	0.0989860155877965
APOE	0.049	0.06	0.011	1.64146746300515e-09
PSMB8	-0.034	0.06	0.094	0.000222265641735926
STAT1	-0.046	0.059	0.106	8.89827132221266e-13
BATF2	-0.017	0.059	0.076	0.201792902590926
ETV7	-0.024	0.059	0.084	0.000448219074361112
RPS6KA4	0.014	0.059	0.045	0.000105009697570251
TNFSF8	0.03	0.058	0.029	0.779035162752087
CARD16	-0.034	0.058	0.092	3.74843827398996e-07
S100A8	0.031	0.058	0.026	7.27163334250446e-05
PSME2	-0.018	0.058	0.077	0.0371822856777997
NLRC5	-0.043	0.058	0.101	1.92970947228956e-07
PSMB10	-0.027	0.058	0.086	0.0170466490900833
PSMB9	-0.066	0.058	0.124	5.87717408997773e-16
IRF7	0.039	0.058	0.02	0.205462924810969
NFKB2	0.002	0.058	0.056	0.183851715381774
NLRC3	0.057	0.057	0	0.000198104389684631
LGALS9	-0.021	0.057	0.078	0.000343810680100754
TNFRSF4	0.034	0.057	0.023	0.000218266824900465
S100A9	0.014	0.056	0.042	0.0418474224244253
SP110	-0.001	0.056	0.057	0.252898895848452
CIITA	-0.062	0.055	0.118	1.44716534369841e-16

Continued on next page

Table S9 – *Continued from previous page*

MR	FC__Median	Median__ICR__Enabled	Median__ICR__Disabled	FDR Adjusted P-value
TMIGD3	-0.013	0.055	0.067	0.374283080197499
NMI	-0.013	0.055	0.068	0.22889920000177
PARP15	-0.033	0.055	0.088	2.99844425589249e-07
PRKCB	0.029	0.053	0.024	1.6196484774862e-06
CD4	-0.036	0.052	0.088	3.20627246397115e-06
CD74	-0.077	0.051	0.127	9.72127694625229e-25
MSC	0.025	0.051	0.026	0.000934590111889505
RHOH	0.051	0.051	0	1.6872189799051e-12
OSM	0.017	0.051	0.034	0.260068396431532
SP100	-0.031	0.051	0.082	2.06105279488908e-09
RELB	0.05	0.05	0	1.14255264835372e-14
PARP14	-0.046	0.05	0.096	2.92324529698177e-11
PTTG1	0.005	0.049	0.044	0.717312061118206
RRM2	0.009	0.049	0.04	0.947655667530999
DDX58	0.047	0.047	0	2.82208921947436e-06
LYL1	-0.009	0.045	0.054	0.525533471111291
CMKLR1	0.017	0.045	0.028	0.00293518522219371
TRIM22	-0.044	0.044	0.087	3.03811434858759e-12
AURKB	0.044	0.044	0	6.2423017683058e-05
MYBL2	0.002	0.044	0.042	0.856223141293583
TLR2	-0.049	0.043	0.091	6.61053142593766e-07
CCNA2	-0.003	0.043	0.045	0.47972451886133
HELZ2	-0.012	0.042	0.054	0.00155741863299682
ICAM1	-0.029	0.042	0.071	0.00114344903549752
SLC11A1	-0.02	0.041	0.061	0.001044701818085
CEBPB	0.002	0.041	0.039	0.871609262046838
PYCARD	-0.026	0.04	0.067	1.3227035020184e-05
DLGAP5	-0.01	0.04	0.05	0.170632824494345
NLRP3	-0.013	0.039	0.052	0.0295828657016058
AKNA	-0.015	0.039	0.054	0.106980897493625
IL16	-0.041	0.037	0.079	2.61161345175868e-06
TLR3	-0.015	0.037	0.052	0.0323025198539572
DTX3L	-0.035	0.037	0.071	8.21638535085964e-08
ORC1	-0.011	0.037	0.049	0.62353880205931
PARP10	-0.029	0.036	0.065	0.0134202831093015
LPXN	-0.053	0.035	0.088	1.80617631302831e-08
PSME1	-0.015	0.034	0.049	0.123915663036177
BIRC5	0.034	0.034	0	6.13468941414913e-05
BCL2L12	-0.007	0.033	0.04	0.0473597636518319
DAB2	-0.038	0.032	0.071	0.00227582165886934
PARP9	-0.065	0.032	0.097	7.65276592070633e-24
PLK1	-0.003	0.032	0.035	0.666930453657902
FLI1	-0.02	0.031	0.051	0.0100643755131777
ENG	-0.025	0.031	0.056	0.00227582165886934
CAV1	-0.01	0.031	0.041	0.103713684457301
PHF11	-0.013	0.031	0.044	0.0971693551615077
EIF2AK2	-0.006	0.03	0.036	0.0202142935242603
CDC45	0.03	0.03	0	0.00321095533458763
CD38	-0.021	0.028	0.048	0.126022628712869
COMMD5	0.024	0.028	0.004	0.0027018290107188
ASF1B	-0.014	0.025	0.04	0.0271938645872068
STK3	-0.006	0.025	0.031	0.199082738214626
KLHL6	-0.052	0.024	0.076	7.92338429085891e-06
DCN	-0.015	0.023	0.038	0.218794698013816
LUM	-0.061	0.022	0.083	1.41219597765441e-10
DEPDC1	-0.017	0.022	0.04	0.00113349894676407
FOXS1	-0.011	0.022	0.032	0.178984763974162
ACTN1	-0.051	0.021	0.072	3.05339575508954e-08
IRF3	-0.028	0.021	0.049	0.00120068906095263

Continued on next page

Table S9 – *Continued from previous page*

MR	FC_Median	Median_ICR_Enabled	Median_ICR_Disabled	FDR Adjusted P-value
GAS7	-0.021	0.021	0.041	0.0314399803681484
CYP1B1	-0.027	0.02	0.048	0.0031445675338866
FOXM1	-0.006	0.02	0.026	0.170632824494345
TEAD2	-0.004	0.019	0.023	0.586838466682856
IFI16	-0.07	0.019	0.089	6.8329202855726e-08
RPS6KA1	-0.055	0.018	0.073	5.41052112860907e-09
CENPF	-0.025	0.018	0.043	7.619246460604e-05
E2F7	-0.003	0.017	0.02	0.0177125188025584
E2F1	-0.002	0.016	0.018	0.602293184068575
SERPINE1	-0.039	0.014	0.054	7.28645225826126e-07
TGFB2	-0.025	0.011	0.036	0.000129416519760507
CDC6	-0.024	0.011	0.035	0.00916279570380372
TRIP13	0.006	0.011	0.005	0.777109761785069
HCLS1	-0.08	0.01	0.09	1.49777413915525e-18
COL1A1	-0.066	0.01	0.075	4.00038287867369e-13
BCL11B	-0.024	0.01	0.034	0.62353880205931
NFAM1	-0.088	0.009	0.097	1.16869527647307e-19
CAVIN1	-0.021	0.009	0.03	0.00047561039839409
BCL6B	0	0.008	0.008	0.997401932562736
IRF9	-0.008	0.008	0.017	0.316503484080799
SIRT7	-0.012	0.006	0.018	0.02675392047184
PTAFR	-0.075	0.002	0.077	1.44375869236148e-12
IKZF3	-0.122	0.001	0.123	7.97685241836754e-48
NOTCH3	-0.01	0.001	0.011	0.165452132219225
BATF	-0.117	0	0.117	1.60947525206948e-45
SP140L	-0.089	0	0.089	1.39742281880237e-31
TMSB4X	-0.08	0	0.08	3.31855703274959e-54
HAMP	-0.072	0	0.072	1.24575512780785e-18
HMOX1	-0.069	0	0.069	1.17294327376419e-10
BCL3	-0.054	0	0.054	3.47080650019954e-09
CEBPA	-0.07	0	0.07	1.3606028583701e-27
LEF1	-0.054	0	0.054	1.51618041931505e-06
PLK3	-0.052	0	0.052	3.41261849861889e-09
LMO2	-0.041	0	0.041	1.35274089107849e-09
STAT2	-0.056	0	0.056	3.20627246397115e-06
HTATIP2	-0.026	0	0.026	0.0212726248144011
VGLL3	-0.018	0	0.018	0.0266603109503057
PRKCH	-0.023	0	0.023	0.00300719045255996
MECOM	-0.003	0	0.003	0.000673808240435722
MXD3	-0.024	0	0.024	0.00302206143122695
ADAM8	-0.021	0	0.021	0.00349310996049077
**TGFB1I1**	-0.027	-0.005	0.022	0.00047561039839409
**ELF1**	-0.047	-0.006	0.042	1.32113906208911e-11
**KANK2**	-0.046	-0.009	0.037	6.3459977125446e-05
**DLL4**	-0.031	-0.014	0.017	0.00180921907835634
**SMO**	-0.026	-0.015	0.01	3.46382380144791e-05
**AR**	-0.075	-0.019	0.056	2.20722855455306e-17
**PABPC1L**	-0.063	-0.04	0.023	1.73204821142261e-18

Table S10. List of 62 MRs when performing analysis w.r.t. ICR Low phenotype. Out of these 57 MRs have high median activity in ICR Low samples (specific to ICR Low) for the 12 cancers of interest and 5 MRs have low median activity in ICR Disabled cancers but high median activity in ICR Enabled cancers (highlighted in bold). The significance of difference in activities in ICR Enabled cancer samples vs ICR Disabled cancer samples is highlighted using the Wilcoxon ranksum test.

MR	FC_Median	Median_ICR_Enabled	Median_ICR_Disabled	FDR Adjusted P-value
ZNF169	-0.012	0.033	0.045	0.0961741072623114
L3MBTL1	-0.035	0.009	0.045	0.0249275896641441
SS18L1	-0.006	0.033	0.04	0.376133508966851
HSF4	-0.012	0.026	0.038	0.983315596278949

Continued on next page

Table S10 – *Continued from previous page*

MR	FC__Median	Median__ICR__Enabled	Median__ICR__Disabled	FDR Adjusted P-value
IPPK	-0.029	0.007	0.036	4.41229118059616e-09
ING5	0.005	0.038	0.034	0.558919371876408
PRKCZ	-0.009	0.022	0.031	0.945732668090683
SMARCC2	0.009	0.037	0.028	0.00362405182843046
PEG3	-0.025	0.002	0.028	1.85026071849894e-08
ZNF770	-0.01	0.017	0.027	0.170848884398629
ZNF696	0.001	0.027	0.025	0.321875294841204
NRIP2	-0.019	0.006	0.025	0.217919112975751
TSSK4	-0.017	0.006	0.024	0.302902264070943
ZNF133	-0.002	0.022	0.024	0.738717205831016
KAT2A	0.008	0.032	0.023	3.72881507655142e-05
ZBTB47	-0.021	0.002	0.023	0.00873883735894614
KAT5	0.005	0.027	0.023	0.122528386557446
NFKBIL1	-0.022	0.001	0.023	0.00250741997793499
PPM1A	0.009	0.032	0.023	0.305146367747523
SETD3	-0.015	0.009	0.023	0.000412584607673553
NR2C2	-0.002	0.02	0.022	0.738717205831016
ZNF423	-0.01	0.013	0.022	0.0251189734676256
APBB3	-0.001	0.022	0.022	0.122528386557446
ZNF337	0.015	0.038	0.022	0.00322870619107578
SALL2	0.022	0.043	0.022	1.40898004369224e-05
ZNF91	0.013	0.035	0.022	0.00113338322594776
ZNF619	0.006	0.027	0.022	0.983315596278949
ZNF789	0.011	0.032	0.021	0.000272827596229084
PDPK1	0.014	0.035	0.021	0.227735542232445
BTRC	0.01	0.03	0.02	0.945732668090683
PIDD1	0.008	0.027	0.019	0.0480773291060998
UBE2V1	-0.007	0.012	0.019	0.302902264070943
SETD5	0.002	0.02	0.019	0.17942603612332
SIRT5	-0.002	0.017	0.019	0.242769236148681
ZNF354B	0.007	0.024	0.017	0.000151059449274665
GLI4	0.004	0.02	0.017	0.166682488523844
PRMT5	-0.009	0.008	0.017	0.342606769383307
ZNF7	0.024	0.04	0.016	7.19786680395502e-07
ZNF3	0.013	0.028	0.016	0.00199759700229081
DMTF1	0.006	0.021	0.015	8.78108295819399e-05
TCERG1	-0.011	0.003	0.014	0.256343636404134
ZNF805	0.023	0.037	0.013	4.83628894417407e-07
RCOR1	0.005	0.019	0.013	0.217919112975751
ZNF852	-0.012	0.001	0.013	0.0817246063402012
NUFIP1	0.003	0.015	0.013	0.983315596278949
TCEAL3	-0.006	0.005	0.011	0.203038545157711
TRIM52	0.009	0.021	0.011	0.00612568000340995
LHX4	0.009	0.02	0.011	0.000239747534066145
CCNT2	0.028	0.039	0.01	7.76912784585066e-09
CREB3L4	0.038	0.047	0.009	6.18294778592565e-06
ZNF214	0.019	0.028	0.009	1.54414496975798e-06
BOLA1	0.012	0.022	0.009	0.022335781283865
HEXIM2	0.024	0.031	0.007	0.0054799303159783
HDAC10	-0.005	0.002	0.007	0.552742282142914
PRDM16	0.006	0.012	0.006	0.713120196375101
EFCAB6	0.023	0.026	0.003	0.00428495639509666
ALKBH4	0.017	0.019	0.002	0.0234291211039842
**TBX2**	0.017	0.016	-0.001	0.000837763011406681
**PABPC1L**	0.036	0.032	-0.003	2.02040031545213e-15
**TGFBR3**	0.009	0	-0.009	0.00993594112141662
**SMO**	0.028	0.005	-0.022	2.39444155480729e-06
**IGF2**	0.032	0.002	-0.03	3.11102827839288e-09

A

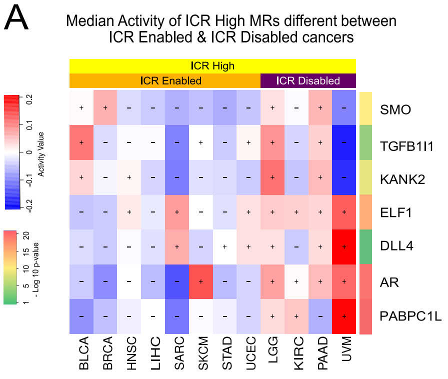

B

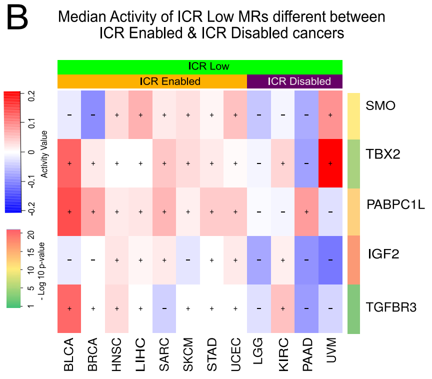

C

	All samples (ICR Enabled + ICR Disabled)					ICR Enabled samples					ICR Disabled samples				
MR	HR	*p* value	Bonferroni corrected p-value	CI lower	CI upper	HR	*p* value	Bonferroni corrected p-value	CI lower	CI upper	HR	*p* value	Bonferroni corrected p-value	CI lower	CI upper
TGFB1I1	3.29	1.00E-03	8.00E-03	1.62	6.68	1.88	0.022	1.78E-01	1.09	3.22	3.39	1.35E-02	1.08E-01	1.29	8.93
TBX2	1.02	9.51E-01	7.61E+00	0.47	2.26	1.66	0.060	4.76E-01	0.98	2.82	0.14	2.60E-03	2.08E-02	0.04	0.50
KANK2	3.20	3.44E-04	2.75E-03	1.69	6.06	1.90	0.037	2.98E-01	1.04	3.47	4.09	5.31E-04	4.25E-03	1.84	9.08
DLL4	1.21	6.17E-01	4.94E+00	0.58	2.53	1.12	0.729	5.83E+00	0.60	2.10	1.18	7.51E-01	6.01E+00	0.43	3.27
ELF1	0.69	3.58E-01	2.86E+00	0.32	1.52	0.70	0.202	1.62E+00	0.41	1.21	1.31	5.75E-01	4.60E+00	0.50	3.43
IGF2	2.40	1.64E-02	1.31E-01	1.17	4.92	1.98	0.019	1.49E-01	1.12	3.49	1.66	5.09E-01	4.07E+00	0.37	7.47
PABPC1L	9.04	3.80E-07	3.04E-06	3.86	21.13	1.37	0.252	2.02E+00	0.80	2.36	104.55	6.53E-18	5.22E-17	36.34	300.82
SMO	3.27	3.75E-03	3.00E-02	1.47	7.29	1.85	0.034	2.70E-01	1.05	3.26	4.08	3.66E-02	2.93E-01	1.09	15.27

D

	ICR High versus ICR Low In Master Regulator High Activity group					ICR High versus ICR Low In Master Regulator Low Activity group				
**MR**	***p value***	***p* value (Bonferroni corrected)**	**HR**	**CI lower**	**CI upper**	***p value***	***p* value (Bonferroni corrected)**	**HR**	**CI lower**	**CI upper**
TGFB1I1	0.11	0.88	1.18	0.96	1.44	0.0035	0.03	0.72	0.58	0.90
TBX2	0.28	1.0	1.13	0.91	1.40	0.0562	0.45	0.82	0.66	1.01
KANK2	0.21	1.0	1.14	0.93	1.39	0.0159	0.12	0.76	0.61	0.95
DLL4	0.89	1.0	0.99	0.80	1.21	0.3930	1.0	0.91	0.74	1.13
ELF1	0.46	1.0	1.08	0.88	1.33	0.0945	0.76	0.83	0.67	1.03
IGF2	0.94	1.0	1.01	0.82	1.24	0.2998	1.0	0.89	0.72	1.11
PABPC1L	0.19	1.0	1.15	0.93	1.42	0.4810	1.0	0.93	0.74	1.15
SMO	0.03	0.24	0.80	0.65	0.98	0.1130	0.90	1.19	0.96	1.48

E

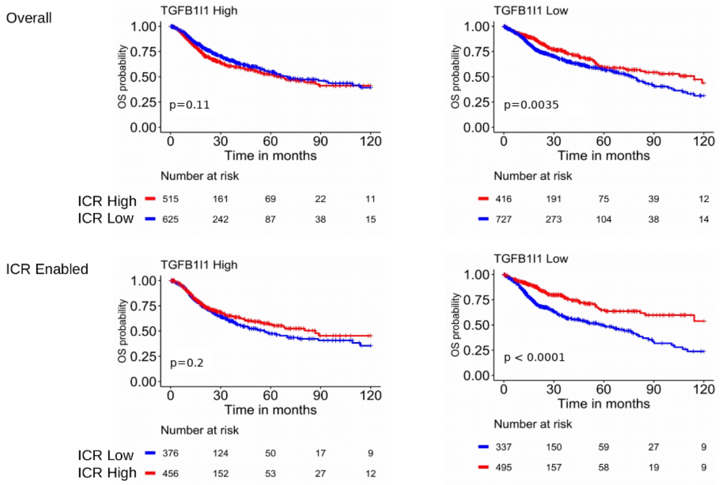

Fig. S6. A) MRs having different median activity patterns in ICR-H samples for ICR-E versus ICR-D cancers (see Supp. Table S9). B) MRs having different median activity patterns in ICR-L samples for ICR-E versus ICR-D cancers (see Supp. Table S10). MRs which were not a TR for a cancer c were given a median activity of 0 (e.g. AR doesn't satisfy the quality control criterion to be a TR for HNSC, KIRC and had 0 median activity and TGFBR3 doesn't satisfy the quality control criterion to be TR for BRCA, STAD and had 0 median activity). C) Statistics of cox proportional hazard regression analysis of MR activity with overall survival, for all samples, in samples of ICR-Enabled cancer types, and in samples of ICR-Disabled cancer types specifically. D) Cox proportional hazard regression analysis comparing survival of ICR High samples versus ICR Low samples by MR subgroup determined by median activity (MR activity high in left, MR activity low in right). E) Kaplan Meier curves showing overall survival (OS) probability of ICR High and ICR Low samples in different TGFB1I1 activity subgroups. P-values in the Kaplan Meier curve represent nominal cox regression p-values.

D. Enrichment Analysis. The 40 significantly enriched protein complexes identified by performing over-expression analysis of 155 consensus MRs specific to ICR-H were highlighted in Supp. Table S11. These included complexes such as IRF1 and IRF9 complex with the CXCL10 promoter from InnateDB (11), DTX3L-PARP9-STAT1 complex from Corum (12) and CD4: IL16 (1212- 1332) from Reactome (13) databases. Several of the genes involved in these complexes were genes that were part of the ICR signature gene panel, thereby providing positive validation as mentioned earlier. We demonstrated the significantly enriched GO Terms and their categories: 1) biological processes, 2) molecular functions and 3) cellular components, in Supp. Fig S7A. The top biological processes included nucleobase-containing compound biosynthetic process, regulation of biosynthetic process, cellular nitrogen compound biosynthetic process, regulation of the primary metabolic process, heterocycle metabolic process, etc. and were primarily associated with the metabolic processes in the cell.

Additionally, we clustered the enriched pathways by estimating similarity in the set of enriched pathways using the extent of overlap between the MRs involved in 2 such pathways. After obtaining the similarity matrix, we performed clustering using spectral clustering (14) to distinguish pathways into cohesive groups (11 in the case of the ICR-H phenotype). The pathways were color-coded by the cluster to which they belonged and ordered based on the ratio of the MRs involved in a pathway to the total number of genes involved in that pathway (see Supp. Fig S8A).

Table S11. The enriched protein complexes based on the MRs specific to ICR Low and ICR High respectively for all the 12 cancers are highlighted here. We showcase the source database from where the protein complex is identified, the gene involved in the protein complex, the size of the protein complex and the effective size represents from the set of genes (23,216 genes) how many match with the genes in the protein complex. The first 4 rows highlight the protein complexes specific to ICR Low whereas the all other rows highlight the protein complexes peculiar to ICR High phenotype respectively.

P-value	P-adj value	Complex Name	Source	Gene Names	Gene Ids	Size	Effective Size
1.00E-05	0.00017	PDPK1:PIP3:PKC zeta	Reactome	PDPK1; PRKCZ	5170; 5590	2	2
0.00022	0.00112	Brg1-associated complex II	CORUM	SMARCC2; PRMT5	6601; 10419	7	7
0.00092	0.00134	emerin C32	PINdb	SMARCC2; RCOR1	6601; 23186	14	14
0.00171	0.00182	AF4.com	PINdb	CCNT2; HEXIM2	905; 124790	20	19
0	5.00E-05	IRF1 and IRF9 complex with the CXCL10 promoter	InnateDB	CXCL10; IRF1; IRF9	3627; 3659; 10379	3	3
0	5.00E-05	ISGF3	Reactome	IRF9; STAT2; STAT1	6772; 6773; 10379	3	3
0	5.00E-05	DTX3L-PARP9-STAT1 complex	CORUM	DTX3L; PARP9; STAT1	6772; 83666; 151636	3	3
5.00E-05	0.00071	STAT dimer_Yp	INOH	STAT4; STAT2; STAT1	6772; 6773; 6775	7	7
8.00E-05	0.00071	p-T611 (S730, S739-FOXM1:MuvB:MYBL2:PLK1 Gene)	Reactome	PLK1; MYBL2; FOXM1	2305; 4605; 5347	8	8
0.00012	0.00071	26S proteasome	Reactome	PSMB10; PSME2; PSME1; PSMB9; PSMB8	5696; 5698; 5699; 5720; 5721	42	42
0.00013	0.00071	CD4:IL16(1212-1332)	Reactome	CD4; IL16	920; 3603	2	2
0.00013	0.00071	CD28:B7-2	Reactome	CD28; CD86	940; 942	2	2
0.00013	0.00071	p-T611 (S730, S739-FOXM1:CENPF Gene)	Reactome	CENPF; FOXM1	1063; 2305	2	2
0.00013	0.00071	p-T611-FOXM1:p-T210-PLK1	Reactome	PLK1; FOXM1	2305; 5347	2	2
0.00013	0.00071	IRF3-P:IRF7-P	Reactome	IRF3; IRF7	3661; 3665	2	2
0.00013	0.00071	HAVCR2:LGALS9	Reactome	LGALS9; HAVCR2	3965; 84868	2	2
0.00013	0.00071	p100:RELB	Reactome	RELB; NFKB2	4791; 5971	2	2
0.00013	0.00071	PA28 complex	CORUM	PSME2; PSME1	5720; 5721	2	2
0.00013	0.00071	S100A8:S100A9:Ca2+	Reactome	S100A9; S100A8	6279; 6280	2	2
0.00013	0.00071	p-STAT2:p-STAT1	Reactome	STAT2; STAT1	6772; 6773	2	2
0.00013	0.00071	p-Y693-STAT4:p-Y701-STAT1	Reactome	STAT4; STAT1	6772; 6775	2	2
0.00013	0.00071	TLR6:TLR2	Reactome	TLR2; TLR6	7097; 10333	2	2
0.00013	0.00071	DTX3L-PARP9 complex	CORUM	DTX3L; PARP9	83666; 151636	2	2
0.00023	0.0008	TCR/CD3/MHC II/CD4/LCK/ZAP-70/CBL/SLAP-2/Ubiquitin	PID	CD4; SLA2; CD3D	915; 920; 84174	13	11
0.0004	0.0008	Chromosomal passenger complex CPC (INCENP,BIRC5, AURKB)	CORUM	BIRC5; AURKB	332; 9212	3	3
0.0004	0.0008	FASLG/FAS (trimer)/Btk	PID	FASLG; BTK	356; 695	3	3
0.0004	0.0008	E2F1-p107-cyclinA complex	CORUM	CCNA2; E2F1	890; 1869	3	3
0.0004	0.0008	alphaM/beta2 Integrin/CD40L	PID	ITGB2; CD40LG	959; 3689	3	3
0.0004	0.0008	BRCA1-IRIS-pre-replication complex	CORUM	CDC6; ORC1	990; 4998	3	3
0.0004	0.0008	alphaL/beta2 Integrin/ICAM1	PID	ITGB2; ICAM1	3383; 3689	3	3
0.0004	0.0008	STAT1 and STAT3 complex with the IRF1 promoter	InnateDB	IRF1; STAT1	3659; 6772	3	3
0.0004	0.0008	IRF1 and IRF9 complex with the ISG15 promoter	InnateDB	IRF1; IRF9	3659; 10379	3	3
0.0004	0.0008	STAT1 and IRF9 complex with the ISG15 promoter	InnateDB	IRF9; STAT1	6772; 10379	3	3
0.0004	0.0008	IFNAR2 forms a complex with IRF9 and STAT2	InnateDB	IRF9; STAT2	6773; 10379	3	3
0.0004	0.0008	CASP8 forms a complex with NLRC4 (IPAF) and PYCARD (ASC)	InnateDB	NLRC4; PYCARD	29108; 58484	3	3
0.0004	0.0008	NLRP3 (Cryopyrin) forms a complex with PYCARD (ASC) and CASP1	InnateDB	PYCARD; NLRP3	29108; 114548	3	3
0.00129	0.00213	CDK:DDK:MCM10:active pre-replicative complex:CDC45	Reactome	CDC45; CDC6; ORC1	990; 4998; 8318	19	19
0.00194	0.00297	TGFB1/TGFBR2/Endoglin/TGFBR1/ALK1/CAV1	PID	ENG; CAV1	857; 2022	6	6
0.0027	0.00393	TCR/CD3/MHC II/CD4	PID	CD4; CD3D	915; 920	9	7
0.00272	0.00393	ECM proteins:Integrin alpha:Integrin beta:Cav-1:Fyn:Shc	INOH	DCN; COL1A1; LUM; ITGB2; CAV1	857; 1277; 1634; 3689; 4060	83	83
0.00357	0.00463	p-T611 (S730, S739-FOXM1:MuvB:MYBL2:CCNB1 Gene)	Reactome	MYBL2; FOXM1	2305; 4605	8	8
0.00565	0.00688	ETS_p:responsive element	INOH	SPI1; FLI1	2313; 6688	10	10
0.0127	0.01395	ECM proteins:Integrin alpha:Integrin beta	INOH	DCN; ITGB2; LUM; COL1A1	1277; 1634; 3689; 4060	78	78
0.04761	0.04857	GPCR ligand:GPCR	INOH	TGFB2; IL16; CD40LG; TMIGD3; CD86; FASLG	356; 942; 959; 3603; 7042; 57413	227	226

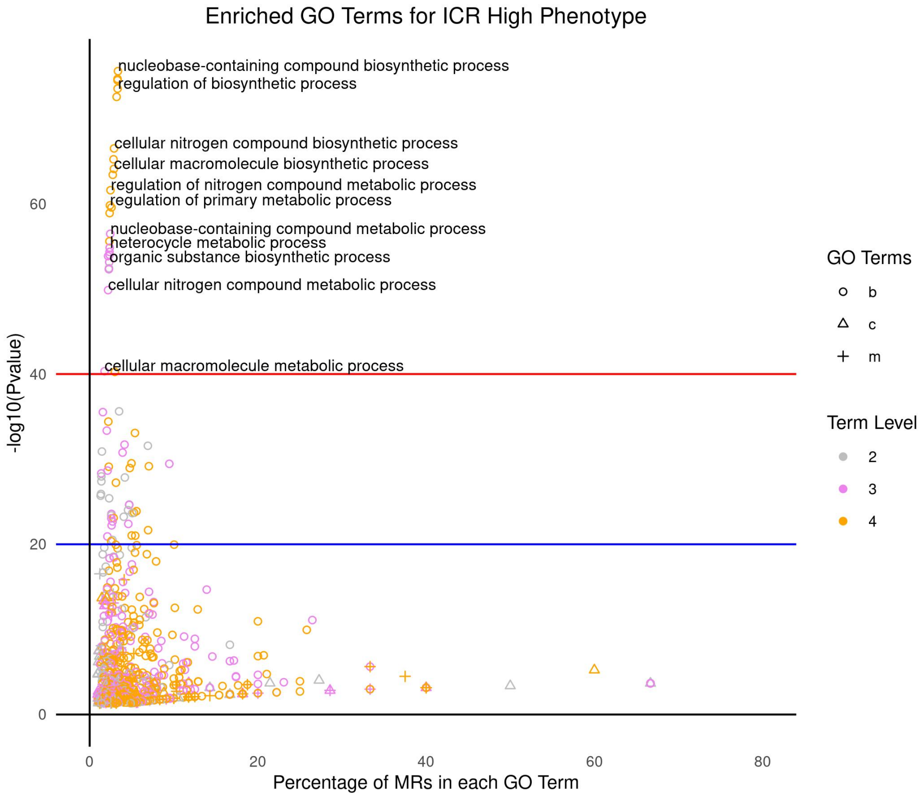

(a) Most significant GO Terms associated with common MRs specific to ICR High phenotype across the 12 cancer types

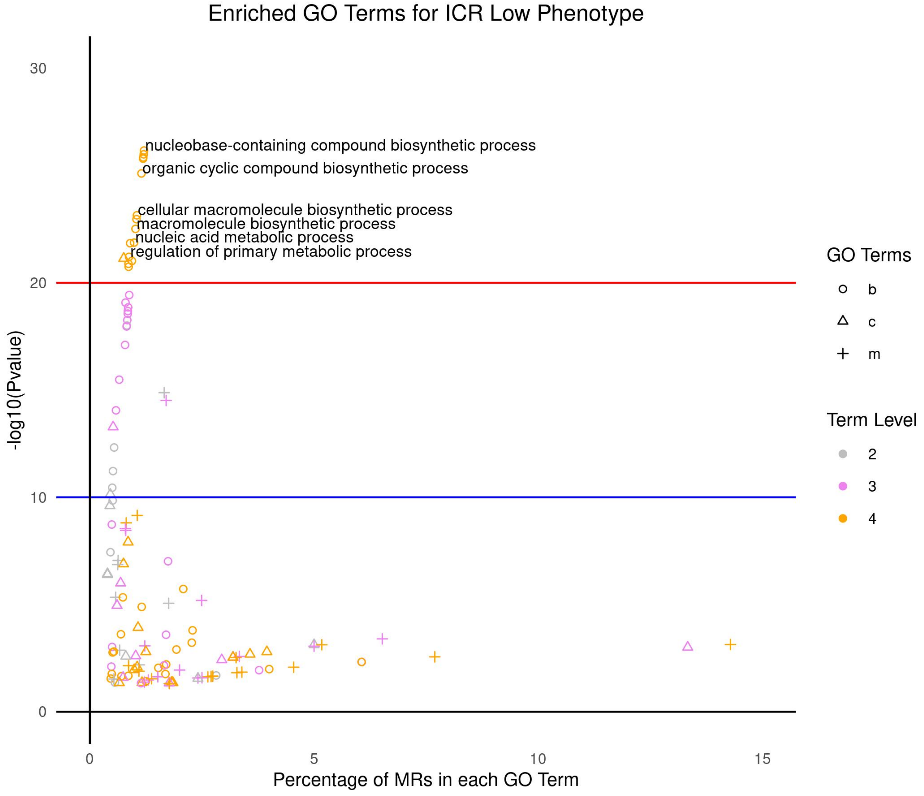

(b) Most significant GO Terms associated with common MRs specific to ICR Low phenotype across the 12 cancer types

Fig. S7. GO Terms including Biological Processes (b), Cellular Components (c), Molecular Functions (m) which are significantly enriched when performing over-expression analysis for common MRs for ICR High and ICR Low phenotype respectively.

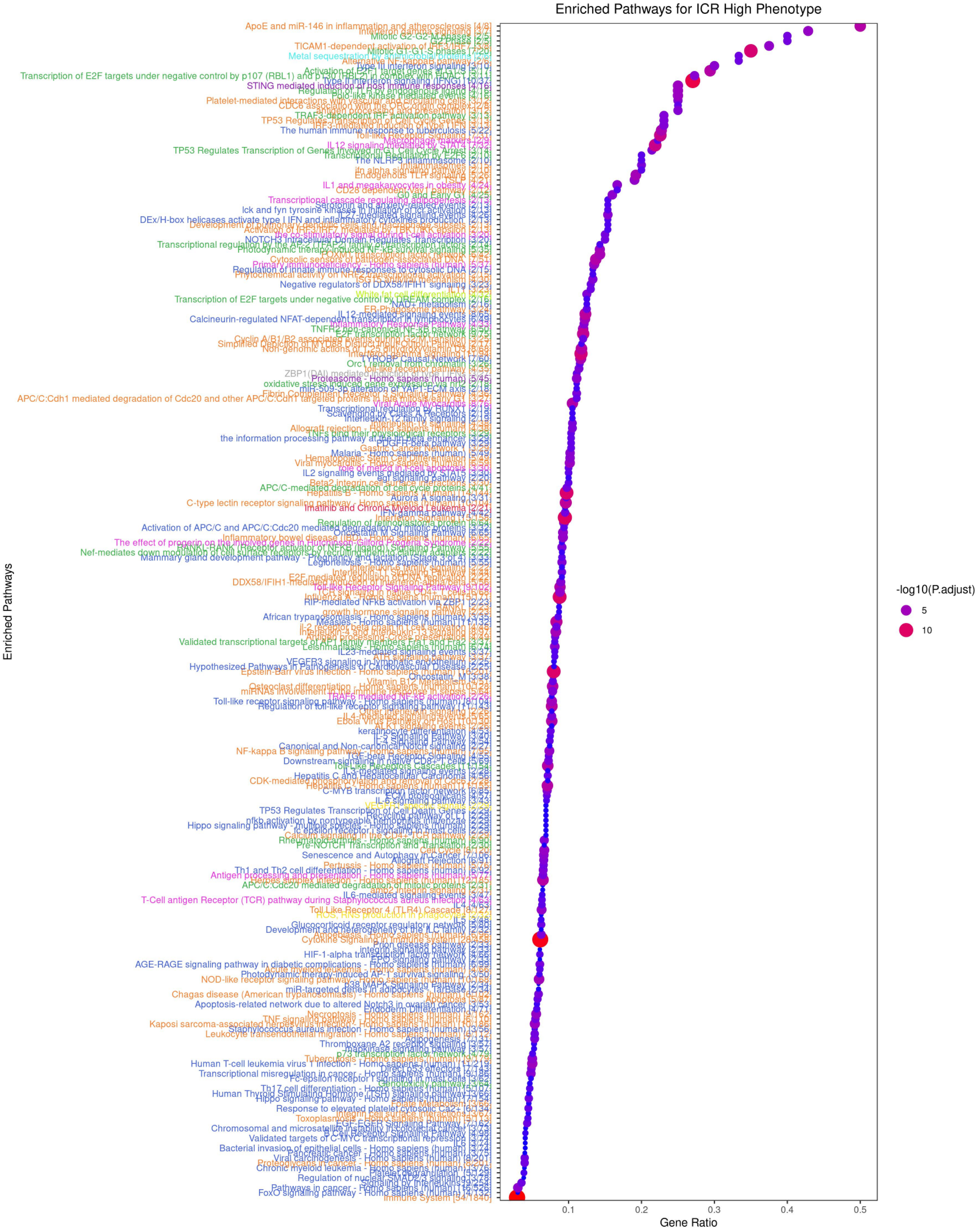

(a) Dotplot showing the ratio between top MRs and the total number of genes in each enriched pathway, where the size of each dot is proportional to $-\log 10(\cdot)$ transformation on the adjusted p-values and the color of each dot increases from blue to red as p-values decreases. The enriched pathways are colored grounded on the cluster to which each pathway belongs based on overlap between the top MRs in the pathways.

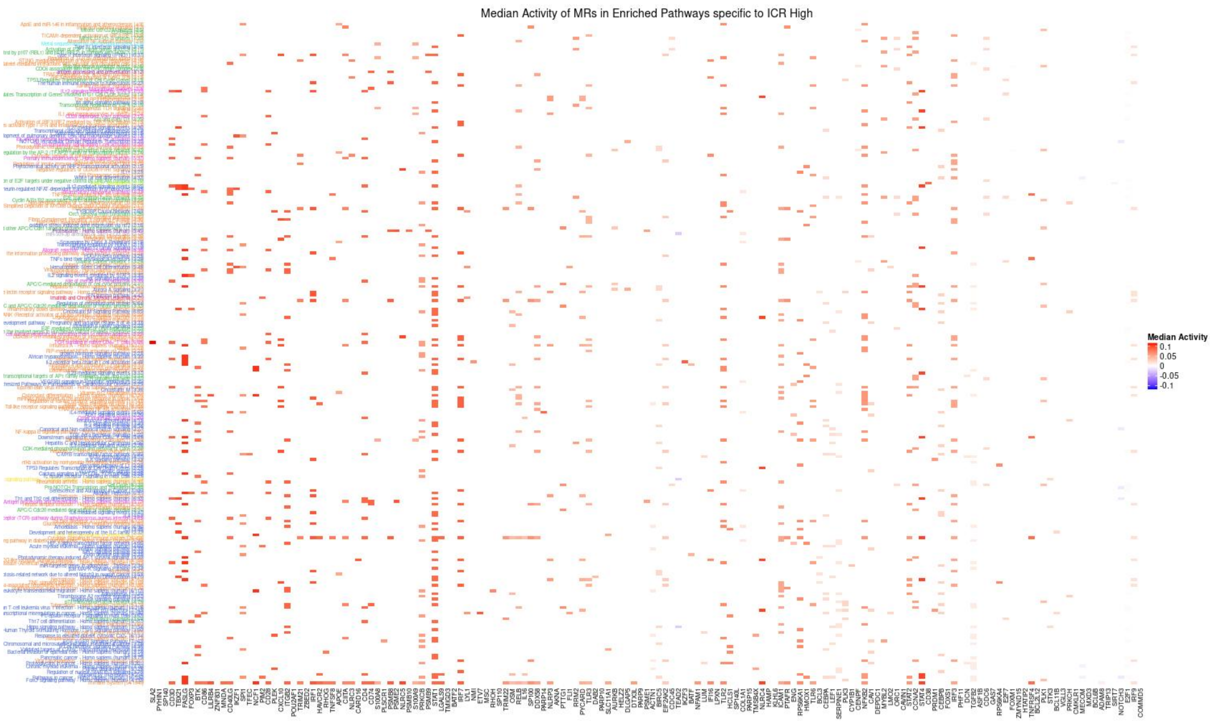

(b) Heat plot showcasing the enriched pathways in which each of the top MRs specific to ICR High phenotype are involved. The intensity represents the median activity for a MR across all the 12 cancers (both ICR Enabled and Disabled cancers).

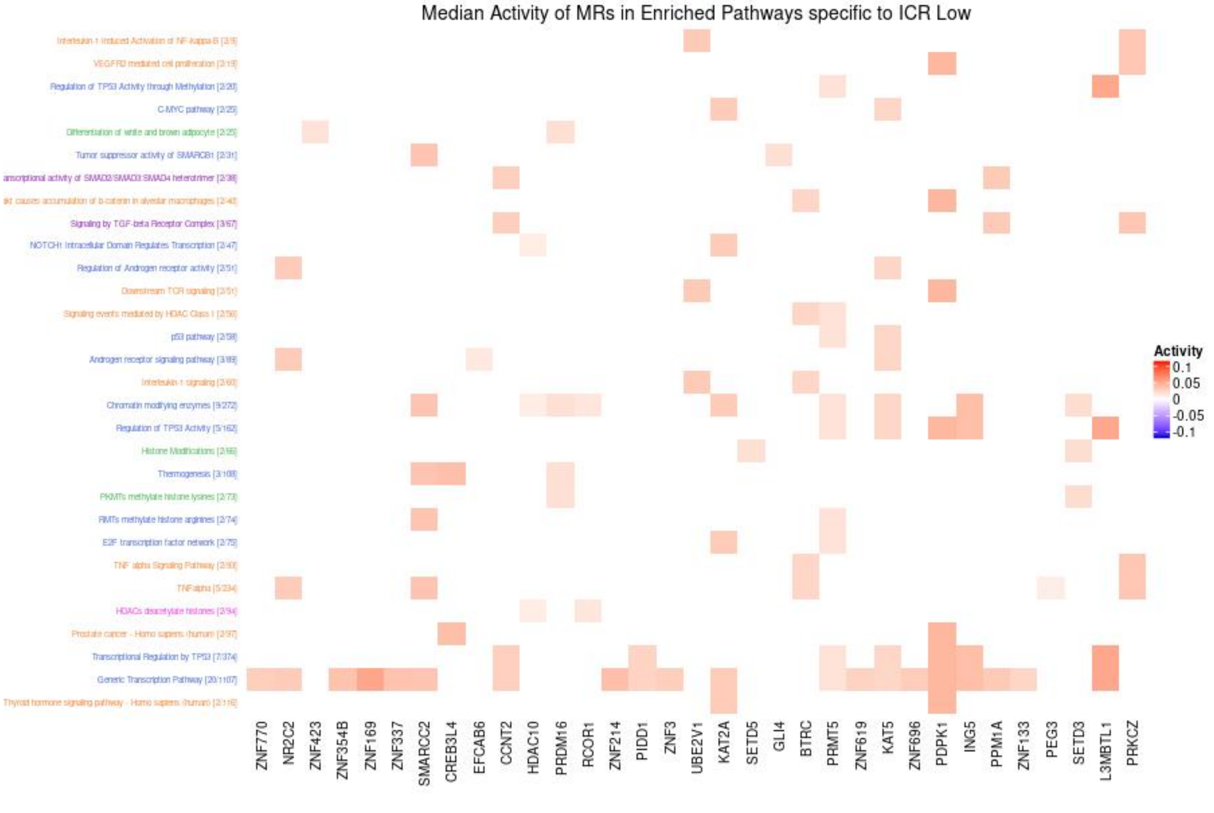

(c) Heat plot showcasing the enriched pathways in which each of the top MRs specific to ICR Low phenotype are involved. The intensity represents the median activity for a MR across all the 12 cancers (both ICR Enabled and Disabled cancers).

Fig. S8. The top enriched pathways obtained by over-expression analysis for the top MRs peculiar to ICR High and ICR Low phenotype are highlighted here. The pathways are clustered and color coded based on the similarity in the MRs involved in these pathways.

Table S12. List of 212 MRs including MRs specific to ICR Low and ICR High phenotype and their activity profile for the set of 20 ICR Neutral cancers. The significance (200 out of 212 MRs) of difference in activities in ICR High vs ICR Low cancer samples is highlighted using the Wilcoxon ranksum test.

MR	FDR Adjusted P-value	Mean_ICR_High	Mean_ICR_Low	FC_Mean
RCOR1	0.00018444291052493	-0.01	0.002	-0.012
PPM1A	1.07304647536921e-10	-0.011	0.01	-0.022
BTRC	3.98144413670395e-12	-0.011	0.011	-0.021
SETD3	8.36012739775122e-05	-0.008	0.01	-0.017
ZNF619	1.26933156142363e-23	-0.021	0.01	-0.031
ZNF91	6.75751105356814e-15	-0.015	0.011	-0.026
PRKCZ	2.7594253534213e-18	-0.02	0.008	-0.028
ZNF214	0.84590137452578	0	-0.003	0.003
PRMT5	1.48297285267166e-12	-0.013	0.014	-0.027
UBE2V1	6.29915049047496e-42	-0.026	0.021	-0.047
KAT5	1.78932852110983e-37	-0.027	0.019	-0.047
ZNF696	6.54864441966842e-70	-0.044	0.029	-0.073
ZNF770	2.1216272406398e-11	-0.014	0.012	-0.026
IPPK	8.85179124914419e-07	-0.01	0.01	-0.021
ZNF805	1.819409386644e-08	-0.012	0.009	-0.02
SETD5	8.3417015387985e-33	-0.026	0.019	-0.045
NR2C2	1.30130551320594e-11	-0.015	0.013	-0.028
SMARCC2	1.35727659685243e-14	-0.021	0.01	-0.031
PDPK1	2.14496311544244e-52	-0.035	0.023	-0.058
LHX4	1.923767981369e-58	-0.033	0.028	-0.06
ZNF337	1.55426001292506e-59	-0.037	0.028	-0.065
TSSK4	7.83672563621395e-43	-0.028	0.023	-0.051
ZNF852	2.9883446325839e-16	-0.018	0.013	-0.031
ZNF423	4.68409962894381e-22	-0.027	0.012	-0.04
PEG3	1.11995989927991e-28	-0.028	0.015	-0.043
ZBTB47	0.00281934265719435	-0.014	-0.002	-0.011
PRDM16	6.46453607502135e-24	-0.029	0.014	-0.044
NRIP2	6.26101324414431e-46	-0.034	0.018	-0.053
TCEAL3	2.16813265749533e-18	-0.024	0.013	-0.037
SALL2	4.29962537203818e-70	-0.045	0.027	-0.073
ZNF7	8.85415706727164e-05	-0.007	0.009	-0.015
GLI4	3.8139772600851e-19	-0.023	0.016	-0.038
HDAC10	0.149858203259015	-0.004	0.002	-0.006
ZNF354B	1.08131217298699e-17	-0.018	0.014	-0.032
HSF4	2.05600409050444e-27	-0.027	0.014	-0.041
DMTF1	2.27032991263742e-10	-0.014	0.009	-0.023
ZNF789	2.92338073492975e-39	-0.033	0.019	-0.052
ZNF169	3.81580578164997e-22	-0.02	0.014	-0.034
TRIM52	4.70178054467524e-21	-0.024	0.014	-0.038
TCERG1	2.50091469081211e-05	-0.011	0.005	-0.016
APBB3	3.96161466697785e-07	-0.01	0.008	-0.017
PIDD1	3.58492085718839e-19	-0.022	0.011	-0.033
KAT2A	1.40404171315504e-30	-0.023	0.019	-0.042
ZNF133	1.81230721531681e-40	-0.037	0.016	-0.053
SS18L1	8.13697588550558e-34	-0.026	0.017	-0.042
L3MBTL1	3.24434836555721e-42	-0.03	0.024	-0.054
CCNT2	7.74619314959946e-31	-0.027	0.016	-0.043
NFKBIL1	2.13174122143131e-41	-0.037	0.019	-0.056
ING5	2.03935238127411e-57	-0.04	0.028	-0.067
CREB3L4	2.43079837843744e-14	-0.015	0.015	-0.029
EFCAB6	3.24739352740684e-68	-0.044	0.027	-0.071
NUFIP1	1.14191370226229e-38	-0.033	0.017	-0.051
SIRT5	4.46412070513584e-21	-0.022	0.011	-0.033
ALKBH4	7.07957522042104e-21	-0.025	0.015	-0.04
BOLA1	1.87375563017503e-06	-0.015	0.006	-0.02
HEXIM2	8.23803824570322e-31	-0.028	0.021	-0.049

Continued on next page

Table S12 – *Continued from previous page*

MR	FDR Adjusted P-value	Mean__ICR__High	Mean__ICR__Low	FC__Mean
ZNF3	1.18725984832175e-32	-0.027	0.022	-0.05
PYCARD	3.73841190494315e-83	0.037	-0.039	0.076
RHOG	3.10506984459294e-127	0.05	-0.048	0.098
CD38	6.28399519439084e-78	0.034	-0.036	0.071
LYL1	6.04132498290458e-65	0.028	-0.032	0.059
TRIM21	7.56237880550687e-67	0.034	-0.033	0.067
LMO2	1.87552326121697e-23	0.022	-0.017	0.039
SP140L	5.98785065378059e-30	0.021	-0.018	0.039
PRDM1	5.55011459009383e-78	0.04	-0.034	0.074
ZBED2	2.05430276471036e-30	0.021	-0.023	0.044
PRKCH	2.48161418598538e-18	0.016	-0.019	0.035
LEF1	5.39274056041322e-06	0.002	-0.015	0.017
BCL11B	0.000116207818389292	0.004	-0.013	0.017
NOTCH3	0.359601776581272	-0.01	-0.004	-0.006
TEAD2	1.69078858522379e-10	0.017	-0.009	0.026
RPS6KA1	3.73794693108462e-05	0.006	-0.013	0.019
TLR3	3.87124524206189e-21	0.022	-0.019	0.041
MECOM	6.64588344733334e-10	-0.022	0.006	-0.028
BCL6B	5.25193234159459e-12	0.009	-0.017	0.026
ENG	2.23052819250214e-24	0.019	-0.025	0.044
FOXS1	0.000819535577820927	0.006	-0.009	0.015
HTATIP2	0.009150676788896	-0.011	0.001	-0.011
CEBPA	0.678553536674199	0	-0.002	0.002
COMMD5	6.15955451003769e-18	0.017	-0.015	0.032
BCL2L12	2.11127615017388e-09	0.016	-0.009	0.025
STK3	4.67868666888714e-05	0.01	-0.002	0.012
MXD3	0.000375595043143986	-0.011	0.005	-0.016
PTTG1	0.00254950487198294	0.005	-0.006	0.01
SIRT7	0.79843930987826	-0.002	-0.001	-0.001
DDX58	3.88252082482911e-105	0.05	-0.043	0.093
PLSCR1	2.53677582287725e-51	0.034	-0.026	0.06
RPS6KA4	1.41892198650495e-34	0.023	-0.023	0.046
NMI	1.27749389674198e-77	0.04	-0.043	0.082
S100A9	1.40966901464846e-67	0.038	-0.041	0.078
S100A8	2.01811490922114e-68	0.036	-0.04	0.076
CEBPB	5.81595564632253e-62	0.037	-0.036	0.073
SLC11A1	1.04966807736392e-65	0.035	-0.036	0.071
OSM	1.4861121481752e-138	0.053	-0.047	0.1
TMIGD3	2.67077283183459e-142	0.06	-0.053	0.113
HMOX1	4.03787803876848e-68	0.038	-0.035	0.073
DAB2	3.91485422246799e-22	0.017	-0.021	0.039
CAVIN1	2.45339990226268e-12	0.014	-0.02	0.034
CAV1	2.18743022421677e-36	0.026	-0.032	0.058
VGLL3	2.27967537095367e-30	0.022	-0.021	0.044
SERPINE1	4.12111983903476e-35	0.026	-0.025	0.051
ACTN1	5.58476843285529e-25	0.02	-0.023	0.043
GAS7	1.90839032255116e-24	0.019	-0.025	0.045
TGFB2	0.0871175124102753	0.005	-0.004	0.009
LUM	4.64578192183921e-29	0.025	-0.026	0.052
DCN	2.75493788947025e-32	0.025	-0.029	0.055
COL1A1	1.7991966262506e-25	0.022	-0.025	0.047
CYP1B1	1.802103210739e-48	0.028	-0.033	0.061
TNFRSF4	4.912450124745e-121	0.051	-0.04	0.091
APOE	7.12104236215371e-125	0.053	-0.045	0.098
LPXN	3.26327603922605e-182	0.068	-0.062	0.13
NLRC4	2.37965446239162e-125	0.055	-0.043	0.098
FOXP3	5.44022471496649e-187	0.073	-0.058	0.131
NFAM1	3.38626548040147e-136	0.057	-0.049	0.106
NLRP3	4.76334068636002e-124	0.055	-0.05	0.105

Continued on next page

Table S12 – *Continued from previous page*

MR	FDR Adjusted P-value	Mean_ICR_High	Mean_ICR_Low	FC_Mean
HAMP	3.81868366389005e-105	0.05	-0.04	0.091
ZMYND15	9.28105370886902e-77	0.041	-0.034	0.075
FLI1	3.9099214530743e-102	0.045	-0.047	0.092
PRKCB	6.37015639221665e-116	0.054	-0.043	0.097
PARP15	2.85913464052204e-133	0.051	-0.048	0.098
NLRC3	7.80906551228755e-130	0.055	-0.045	0.1
POU2AF1	2.63639868068058e-150	0.062	-0.059	0.122
PIM2	2.1395531026351e-126	0.054	-0.049	0.103
AKNA	1.54710108788709e-99	0.049	-0.045	0.093
CD40LG	6.7064107664725e-143	0.061	-0.056	0.118
ZNF831	2.96822401435502e-142	0.056	-0.054	0.111
HCLS1	7.32323889981377e-134	0.048	-0.05	0.097
KLHL6	3.71983669115627e-161	0.064	-0.058	0.122
LGALS9	6.61043407882246e-151	0.063	-0.057	0.12
CD4	5.41099421122385e-183	0.069	-0.063	0.132
ITGB2	1.86858291541057e-173	0.072	-0.061	0.133
TNFSF8	5.06850761914298e-176	0.068	-0.064	0.132
IKZF1	5.42461920590103e-183	0.072	-0.067	0.138
IL16	2.22377973041977e-148	0.059	-0.06	0.119
CD28	1.49653477823276e-165	0.066	-0.062	0.127
CD3D	3.15996374081027e-206	0.071	-0.067	0.139
PYHIN1	9.21734928760465e-223	0.084	-0.076	0.16
FASLG	1.44227679901715e-203	0.077	-0.061	0.138
RHOH	1.28318075904603e-214	0.08	-0.072	0.151
SLA2	3.01928961598453e-250	0.095	-0.087	0.181
IKZF3	1.82517217914268e-212	0.08	-0.071	0.152
BATF	4.47620371161654e-159	0.066	-0.059	0.125
CD74	3.6714429342889e-160	0.073	-0.059	0.131
CIITA	5.57309671381969e-145	0.065	-0.051	0.116
TRIM22	1.72554134543924e-142	0.065	-0.052	0.117
TLR2	2.07335613634051e-94	0.054	-0.045	0.099
PARP14	5.71249816909901e-138	0.057	-0.042	0.099
SP100	9.9938831877481e-111	0.055	-0.045	0.1
NLRC5	1.08339844043897e-182	0.073	-0.052	0.125
STAT1	4.57888160649282e-175	0.07	-0.049	0.118
IRF1	1.31121629082324e-214	0.079	-0.063	0.143
CXCL10	2.09813719257734e-259	0.091	-0.072	0.163
TBX21	2.04789988425786e-245	0.087	-0.072	0.16
PTAFR	2.33887921528734e-163	0.068	-0.054	0.122
MNDA	2.0924893969933e-173	0.072	-0.059	0.13
CMKLR1	2.03803728672466e-185	0.069	-0.063	0.132
TFEC	4.54714213086394e-202	0.077	-0.062	0.139
LILRB4	5.54113800920652e-228	0.08	-0.062	0.142
NCF1	5.22391871591666e-240	0.085	-0.071	0.155
BTK	8.96221430392592e-201	0.079	-0.066	0.144
SP140	4.59328125133419e-237	0.089	-0.075	0.164
PLEK	5.72078402683568e-192	0.07	-0.063	0.134
SPI1	1.10347364476731e-173	0.067	-0.055	0.123
HAVCR2	1.99723089258321e-222	0.074	-0.062	0.136
CD86	3.30108940601282e-194	0.071	-0.06	0.131
IFI16	5.25162899975951e-86	0.049	-0.043	0.092
MSC	5.49433216394937e-112	0.055	-0.043	0.099
RELB	5.09147063560536e-119	0.052	-0.044	0.096
NFKB2	1.8746076586552e-118	0.053	-0.041	0.095
TLR6	4.4168123764612e-99	0.051	-0.036	0.087
BCL3	2.01773452661136e-72	0.038	-0.031	0.07
STAT4	6.05250723111166e-136	0.053	-0.044	0.097
ADAM8	2.04399277324474e-93	0.057	-0.037	0.094
ICAM1	2.49406088641245e-105	0.055	-0.043	0.098

Continued on next page

Table S12 – *Continued from previous page*

MR	FDR Adjusted P-value	Mean_ICR_High	Mean_ICR_Low	FC_Mean
PLK3	1.22002235690825e-65	0.033	-0.037	0.071
PHF11	4.13377266749917e-47	0.027	-0.027	0.054
PARP10	3.92504703363981e-52	0.025	-0.026	0.051
PSME1	5.66668890582929e-76	0.034	-0.033	0.067
PSME2	1.96132884834267e-154	0.056	-0.043	0.1
IRF3	2.55214054560306e-31	0.019	-0.021	0.04
TMSB4X	1.30887745047632e-48	0.027	-0.03	0.058
PSMB9	2.01869756737636e-195	0.07	-0.06	0.13
PSMB8	5.59168336121078e-161	0.055	-0.051	0.106
PSMB10	2.40107560590299e-156	0.064	-0.054	0.117
CARD16	3.36666393213642e-163	0.058	-0.05	0.108
ETV7	1.72554134543924e-142	0.057	-0.041	0.098
BATF2	1.31531050546897e-119	0.059	-0.044	0.103
SP110	4.92686047603665e-115	0.053	-0.043	0.097
IRF7	8.54790949960488e-115	0.052	-0.04	0.092
PARP9	1.40686809670338e-125	0.059	-0.041	0.1
DTX3L	4.10820847678106e-113	0.053	-0.04	0.093
EIF2AK2	3.18179884944527e-25	0.023	-0.013	0.036
HELZ2	6.49023715940293e-43	0.034	-0.021	0.055
STAT2	1.20994386396319e-97	0.047	-0.039	0.086
IRF9	3.7419898433306e-70	0.038	-0.026	0.064
FOXM1	0.00278064066774352	-0.009	0.004	-0.013
PLK1	0.142450147457807	0	-0.005	0.006
DEPDC1	0.558964116831133	-0.001	-0.004	0.004
RRM2	0.000114379527026426	0.006	-0.012	0.018
BIRC5	0.00187085022044636	0.005	-0.009	0.014
AURKB	0.000405001296808243	0.007	-0.01	0.017
CCNA2	1.57946893998877e-07	0.012	-0.012	0.024
CDC6	0.700640560996662	-0.002	-0.001	-0.001
E2F7	0.0651388660822939	-0.009	-0.001	-0.007
CENPF	0.0609412254724756	-0.004	0.004	-0.008
MYBL2	2.75173612182074e-05	0.012	-0.008	0.02
E2F1	0.055151253353243	-0.005	0.002	-0.007
ASF1B	0.00325979999823571	0.008	-0.006	0.014
CDC45	9.53087920545697e-10	0.017	-0.012	0.029
DLGAP5	5.97197387175504e-11	0.018	-0.013	0.031
ORC1	0.00031245839263336	0.008	-0.009	0.016
TRIP13	0.000945745706904631	0.011	-0.005	0.015

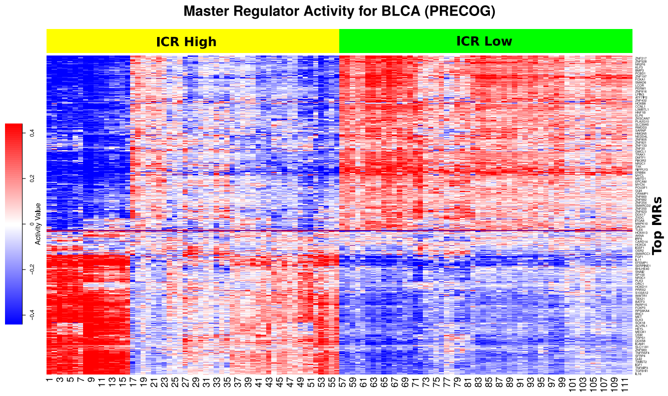

(a) Top MR activity for BLCA in PRECOG repository

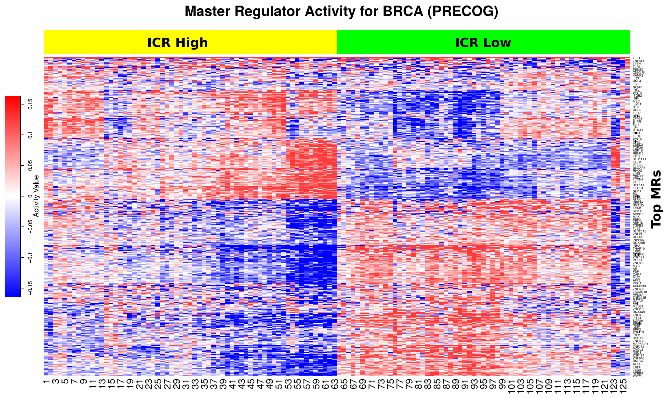

(b) Top MR activity for BRCA in PRECOG repository

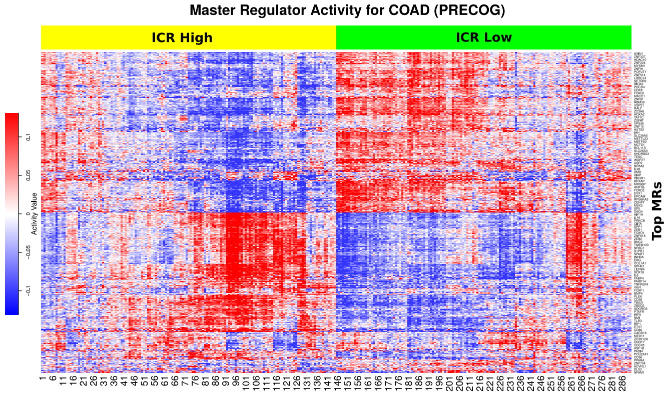

(c) Top MR activity for COAD in PRECOG repository

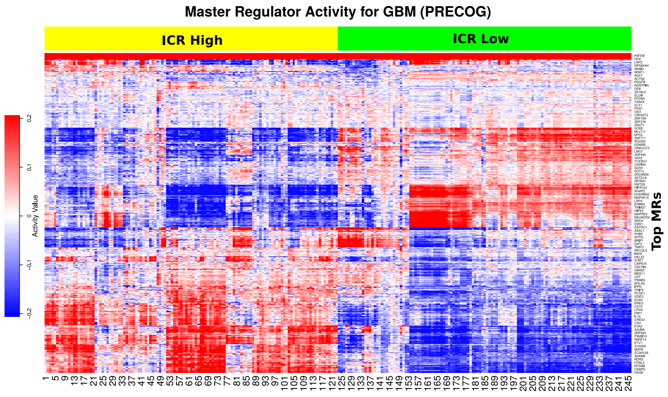

(d) Top MR activity for GBM in PRECOG repository

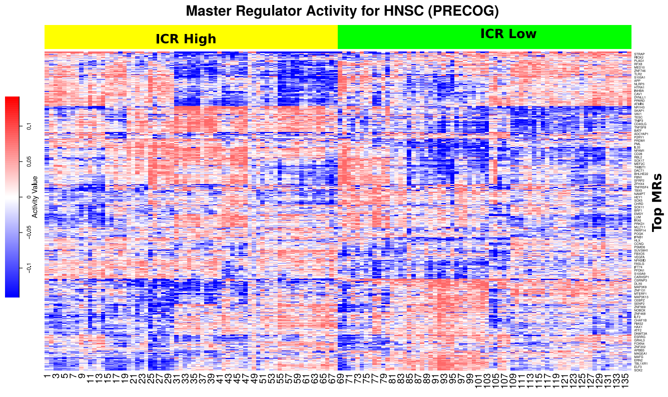

(e) Top MR activity for HNSC in PRECOG repository

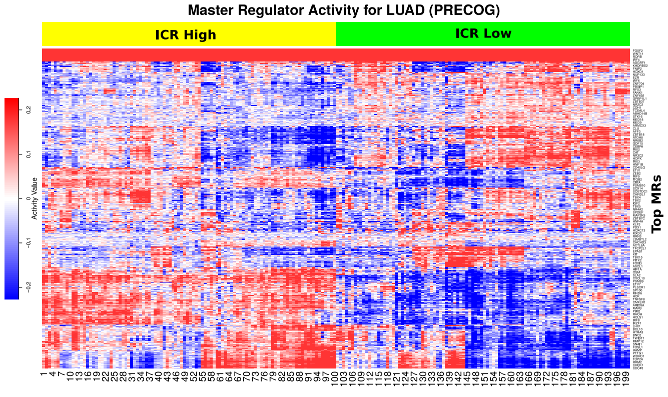

(f) Top MR activity for LUAD in PRECOG repository

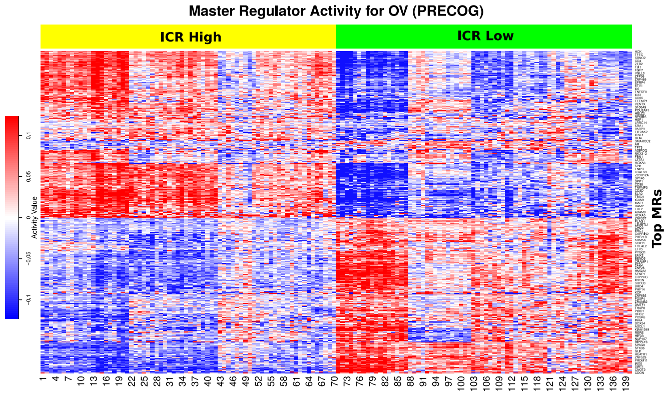

(g) Top MR activity for OV in PRECOG repository

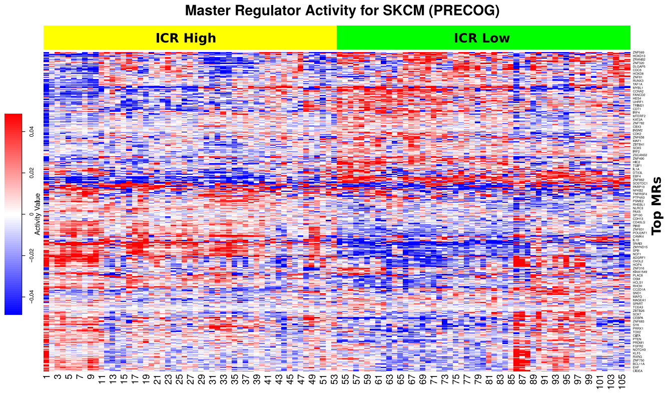

(h) Top MR activity for SKCM in PRECOG repository

Fig. S9. Reverse activity patterns are visible for each cancer type in the PRECOG repository datasets for majority of the myriad top differentially activated master regulators (Top MRs) identified for corresponding cancer type using the RGBM + FGSEA MRA pipeline.

Table S13. List of 212 MRs including MRs specific to ICR Low and ICR High phenotype and their activity profile for the set of 8 PRECOG cancer datasets. The significance (201 out of 212 MRs) of difference in activities in ICR High vs ICR Low cancer samples is highlighted using the Wilcoxon ranksum test.

MR	FDR Adjusted P-value	Mean_ICR_High	Mean_ICR_Low	FC_Mean
PRDM16	0.000206403328474853	-0.009	0.007	-0.016
SALL2	9.15131295297119e-18	-0.017	0.018	-0.035
RCOR1	0.0239424586527336	0.003	-0.004	0.007
PRMT5	0.00368902964268933	-0.008	0.004	-0.012
IPPK	1.77006345391959e-12	0.01	-0.015	0.025
SETD3	0.0248484568829337	-0.005	0.003	-0.008
PPM1A	6.77276003629573e-16	-0.014	0.011	-0.025
ZNF770	0.010726941277807	-0.003	0.002	-0.005
TCEAL3	0.361028092820285	0.001	-0.003	0.005
ZBTB47	0.000282167448983829	0.008	-0.009	0.017
PEG3	1.39352214372356e-16	-0.021	0.016	-0.037
BTRC	1.05579058621762e-78	-0.039	0.037	-0.077
PDPK1	1.23303059317858e-56	-0.03	0.027	-0.057
NRIP2	1.03917071411858e-52	-0.032	0.028	-0.06
ZNF423	4.34044485796121e-36	-0.026	0.03	-0.056
LHX4	6.95892405929949e-76	-0.03	0.028	-0.058
NR2C2	2.28652372727624e-34	-0.018	0.016	-0.033
SETD5	8.42411404226846e-37	-0.024	0.02	-0.044
ZNF91	3.16288660532335e-99	-0.036	0.036	-0.072
CCNT2	2.33454989894475e-57	-0.031	0.029	-0.061
SMARCC2	8.14206863615605e-51	-0.026	0.029	-0.055
PRKCZ	8.96772011688216e-51	-0.035	0.035	-0.07
UBE2V1	1	0	0	0
TSSK4	1.11452115053649e-14	-0.008	0.007	-0.015
ZNF354B	2.83130151461895e-18	-0.013	0.01	-0.023
ZNF852	5.44933120178028e-14	-0.01	0.01	-0.02
ZNF169	1.01743948084733e-37	-0.017	0.018	-0.035
ZNF619	3.60903406835817e-16	-0.014	0.016	-0.03
NFKBIL1	6.08442978292338e-22	-0.02	0.014	-0.034
HDAC10	0.480646241200906	-0.002	0.001	-0.003
GLI4	1.67716152787949e-10	-0.011	0.009	-0.02
ZNF7	4.83403405348585e-17	-0.016	0.013	-0.029
ZNF805	0.130546003467565	0.003	-0.007	0.01
APBB3	3.31359464193933e-13	-0.011	0.009	-0.021
HSF4	2.44742627727189e-36	-0.02	0.019	-0.039
TRIM52	1.94291259347725e-37	-0.022	0.019	-0.041
DMTF1	6.23903238756641e-19	-0.013	0.011	-0.024
ZNF789	1.28549986036817e-27	-0.015	0.015	-0.029
ZNF337	3.44801108047321e-43	-0.018	0.021	-0.039
L3MBTL1	4.43503108286113e-77	-0.03	0.031	-0.061
EFCAB6	4.08707474132188e-16	-0.017	0.013	-0.03
ZNF214	0.00353477555343026	-0.006	0.003	-0.008
CREB3L4	2.29266977779879e-05	-0.009	0.007	-0.016
HEXIM2	1.17758576925862e-10	-0.009	0.009	-0.018
BOLA1	5.35343092111726e-13	-0.014	0.012	-0.026
SIRT5	1.02420065470419e-05	-0.01	0.01	-0.02
ALKBH4	2.27110216912895e-20	-0.016	0.015	-0.031
NUFIP1	2.25038352607229e-26	-0.018	0.017	-0.035
ZNF3	2.23119947946399e-30	-0.021	0.021	-0.042
TCERG1	3.38190769744288e-26	-0.019	0.017	-0.036
SS18L1	3.8298918288129e-65	-0.03	0.027	-0.057
KAT2A	4.3344343192166e-30	-0.02	0.018	-0.038
ING5	3.88425747866491e-30	-0.018	0.018	-0.036
ZNF133	3.64675475000408e-48	-0.032	0.027	-0.059
KAT5	5.77479238511655e-34	-0.022	0.022	-0.044
PIDD1	0.0345840381690182	0.017	0.026	-0.008

Continued on next page

Table S13 – *Continued from previous page*

MR	FDR Adjusted P-value	Mean_ICR_High	Mean_ICR_Low	FC_Mean
ZNF696	2.39596211490874e-11	0.008	0.036	-0.028
NFAM1	1.15605699236821e-83	0.038	-0.043	0.081
PARP15	1.44217489630989e-71	0.031	-0.035	0.066
DAB2	1.57089473204299e-52	0.031	-0.038	0.069
RELB	1.11893768219725e-86	0.046	-0.051	0.096
ADAM8	9.05991262262704e-30	0.012	-0.04	0.052
NFKB2	3.05322985878191e-82	0.029	-0.061	0.09
SLC11A1	2.50768407850629e-94	0.042	-0.047	0.089
OSM	7.06226363382044e-95	0.042	-0.046	0.088
RHOG	1.40471989380792e-122	0.051	-0.055	0.106
S100A9	1.57523733897767e-95	0.041	-0.049	0.09
S100A8	2.06026420750453e-115	0.047	-0.055	0.102
HMOX1	2.57966091265403e-65	0.034	-0.035	0.068
HAMP	3.21193135515379e-107	0.049	-0.054	0.103
CEBPB	7.73036959754727e-75	0.04	-0.049	0.089
CAVIN1	2.83129093305402e-77	0.039	-0.042	0.081
CAV1	6.44721455908312e-52	0.028	-0.034	0.062
ACTN1	7.28385475349411e-75	0.037	-0.042	0.079
PLK3	5.82468821920758e-70	0.034	-0.039	0.073
SERPINE1	1.17765733375952e-49	0.029	-0.038	0.067
LUM	1.13419304218106e-51	0.03	-0.035	0.065
DCN	2.36701210924893e-66	0.033	-0.039	0.072
ENG	3.91458408734729e-69	0.033	-0.038	0.071
COL1A1	1.07499062041526e-34	0.027	-0.031	0.058
NOTCH3	8.01513831620514e-40	0.027	-0.032	0.059
PRDM1	1.56724981287316e-96	0.044	-0.048	0.092
BATF	1.50202032252826e-62	0.031	-0.035	0.066
PYCARD	1.74067308987019e-43	0.024	-0.031	0.054
PARP10	2.1409918170032e-53	0.026	-0.028	0.054
PHF11	1.57280655683716e-53	0.028	-0.03	0.058
TLR6	5.10200459300197e-71	0.036	-0.039	0.075
TLR3	3.96052712493504e-52	0.029	-0.03	0.059
ZMYND15	2.3181042431943e-86	0.032	-0.034	0.066
RPS6KA1	1.96843413278281e-44	0.023	-0.027	0.05
CYP1B1	1.25603995479446e-46	0.025	-0.031	0.056
ICAM1	7.58814018832336e-91	0.045	-0.05	0.095
BCL3	3.13388122220595e-54	0.033	-0.036	0.069
PLEK	4.42884484676936e-136	0.044	-0.046	0.091
CD86	1.36340880862385e-124	0.047	-0.05	0.096
TMIGD3	1.28628099222965e-110	0.041	-0.044	0.085
HAVCR2	1.83212034657233e-113	0.041	-0.044	0.085
CD3D	5.59820554033671e-137	0.045	-0.047	0.092
LYL1	4.47568044168682e-22	-0.012	-0.053	0.041
NLRP3	5.53499636271117e-74	0.039	-0.044	0.083
TLR2	4.21741275031728e-46	0.032	-0.035	0.066
TRIM22	3.84679774008698e-102	0.046	-0.047	0.093
SP100	3.57778288388976e-88	0.043	-0.044	0.086
CD74	2.99986003427541e-113	0.049	-0.05	0.099
CIITA	2.03381438807689e-105	0.043	-0.046	0.089
CD4	5.77973295118976e-132	0.051	-0.053	0.104
BTK	2.6320281818243e-138	0.051	-0.055	0.107
IL16	3.47337190097498e-101	0.047	-0.05	0.096
ITGB2	1.40537706500798e-132	0.052	-0.054	0.107
MNDA	1.60480015642876e-123	0.049	-0.052	0.101
HCLS1	1.370353818771e-102	0.038	-0.045	0.083
TFEC	9.89532817982932e-139	0.051	-0.055	0.106
LGALS9	5.85080171320664e-103	0.045	-0.048	0.094
PTAFR	3.37924049918871e-92	0.04	-0.042	0.082
CMKLR1	3.51454653851247e-106	0.046	-0.05	0.096

Continued on next page

Table S13 – *Continued from previous page*

MR	FDR Adjusted P-value	Mean_ICR_High	Mean_ICR_Low	FC_Mean
NCF1	2.00224949620734e-149	0.052	-0.055	0.107
LILRB4	2.15135493801516e-147	0.058	-0.061	0.119
KLHL6	1.63171732634089e-84	0.035	-0.036	0.071
SP140	4.24156696395186e-139	0.049	-0.051	0.1
TBX21	3.35743107715482e-127	0.052	-0.055	0.107
RHOH	1.52987851985316e-146	0.058	-0.064	0.122
SLA2	4.41045644149198e-162	0.057	-0.061	0.119
TNFSF8	5.98598547091216e-119	0.051	-0.055	0.107
CD28	3.2449640508105e-73	0.037	-0.043	0.08
IKZF3	1.61869573854845e-82	0.037	-0.04	0.077
LPXN	5.23883938257168e-91	0.046	-0.048	0.094
SP140L	2.79068975182887e-59	0.031	-0.034	0.064
TRIM21	6.61764963853388e-72	0.04	-0.041	0.081
NMI	2.56478020515248e-100	0.052	-0.056	0.107
DTX3L	2.96380126910746e-90	0.038	-0.042	0.081
PARP14	2.86554242134782e-99	0.039	-0.043	0.082
PARP9	1.97718804105109e-95	0.043	-0.045	0.088
DDX58	3.30439801237448e-86	0.037	-0.045	0.082
BATF2	1.39887037214496e-75	0.04	-0.038	0.077
IRF7	2.16966165412657e-61	0.034	-0.04	0.074
HELZ2	8.63195698324671e-55	0.03	-0.035	0.065
SP110	3.0154793495933e-49	0.029	-0.031	0.06
PSMB10	1.01334688124475e-97	0.043	-0.046	0.089
CARD16	1.21207344051578e-66	0.037	-0.039	0.076
PSME1	1.27968650110365e-82	0.036	-0.04	0.076
PSMB9	5.68528713749464e-158	0.058	-0.062	0.12
PSMB8	3.96726081331361e-136	0.051	-0.055	0.107
PSME2	3.46869329727386e-145	0.052	-0.057	0.109
ETV7	6.4809281738095e-118	0.045	-0.049	0.094
PLSCR1	6.25137062892311e-108	0.048	-0.051	0.099
IRF1	4.59400582517855e-151	0.058	-0.061	0.12
CXCL10	1.53748658951821e-159	0.059	-0.063	0.122
NLRC5	7.2131961554027e-121	0.044	-0.047	0.091
STAT1	7.19840874513979e-121	0.046	-0.054	0.1
IFI16	4.73181634383497e-46	0.027	-0.024	0.051
RPS6KA4	7.2067734502722e-42	0.024	-0.028	0.052
EIF2AK2	4.34044485796121e-36	0.022	-0.026	0.048
LMO2	7.3839692202764e-27	0.02	-0.018	0.038
SIRT7	0.375463669718953	-0.002	0.001	-0.003
IRF9	8.46504172492354e-23	0.013	-0.014	0.027
TMSB4X	1	0	0	0
BCL11B	0.887248346609485	0.001	-0.003	0.004
PRKCH	1.67012929575511e-21	0.012	-0.012	0.024
ZBED2	3.36044855568177e-25	0.014	-0.013	0.027
HTATIP2	0.0622409761160538	0.002	-0.003	0.005
LEF1	3.855606581522e-19	0.015	-0.018	0.032
BCL6B	6.44605516101448e-09	0.009	-0.008	0.017
FOXS1	2.1403852469302e-25	0.015	-0.019	0.034
TGFB2	2.54542917329132e-17	0.014	-0.018	0.032
MECOM	1.71354825126156e-16	-0.016	0.015	-0.031
CEBPA	9.10463567369924e-05	-0.008	0.006	-0.014
TEAD2	4.61883424383021e-13	0.013	-0.014	0.028
COMMD5	1.7114223357127e-15	0.011	-0.014	0.025
IRF3	1.57511709763627e-09	0.011	-0.016	0.028
BCL2L12	2.10177921284797e-18	0.019	-0.021	0.04
STK3	9.34813170672635e-32	0.024	-0.03	0.054
STAT2	1.87420090426217e-46	0.05	-0.009	0.059
NLRC4	2.14838516634892e-28	0.032	0.007	0.025
TNFRSF4	1.08473560529594e-32	0.045	-0.004	0.049

Continued on next page

Table S13 – *Continued from previous page*

MR	FDR Adjusted P-value	Mean_ICR_High	Mean_ICR_Low	FC_Mean
FOXP3	2.99188285753772e-43	0.05	-0.005	0.054
AKNA	1.71354068926505e-29	0.016	-0.019	0.035
NLRC3	9.24598654381856e-75	0.026	-0.027	0.053
APOE	2.1180991896571e-80	0.034	-0.036	0.07
CD40LG	2.97378928001626e-80	0.034	-0.035	0.069
POU2AF1	1.23471913477714e-65	0.03	-0.03	0.06
PIM2	4.01223948487326e-73	0.032	-0.038	0.07
VGLL3	2.24517763860545e-23	0.016	-0.018	0.035
MSC	4.58578442574319e-82	0.037	-0.039	0.076
FLI1	1.37811153195923e-87	0.037	-0.04	0.077
IKZF1	4.8983472238353e-94	0.038	-0.038	0.075
GAS7	3.04600936912407e-21	0.016	-0.019	0.035
PRKCB	1.5897486931173e-42	0.026	-0.035	0.06
SPI1	1.38140122119995e-104	0.033	-0.032	0.065
FASLG	2.68481863563389e-74	0.028	-0.027	0.055
ZNF831	5.06383808861693e-45	0.019	-0.023	0.041
CD38	9.48835826365546e-31	0.018	-0.017	0.035
STAT4	2.96315008224933e-38	0.022	-0.022	0.044
PYHIN1	2.758271744871e-67	0.062	-0.008	0.07
CENPF	0.828865280907893	-0.004	-0.006	0.002
MYBL2	0.0579447853836595	0.001	-0.009	0.009
E2F1	0.579604354293028	-0.006	-0.003	-0.004
MXD3	5.20331419685961e-08	-0.013	0.009	-0.022
ASF1B	2.94838920427373e-05	0.005	-0.015	0.02
PTTG1	1.2107203358776e-20	0.013	-0.023	0.036
TRIP13	0.0281220624406029	0	-0.01	0.01
CDC45	7.56275509985707e-09	0.009	-0.018	0.026
BIRC5	8.15785286136423e-12	0.01	-0.018	0.028
CDC6	0.000354197496154382	0.004	-0.012	0.016
ORC1	6.81218701284979e-20	0.015	-0.023	0.038
AURKB	1.28759298361704e-20	0.015	-0.024	0.04
FOXM1	4.90343296666309e-08	0.006	-0.015	0.022
PLK1	5.45480528371834e-18	0.012	-0.024	0.036
DLGAP5	1.92170639360552e-20	0.017	-0.025	0.042
CCNA2	1.70256492083021e-27	0.02	-0.029	0.049
DEPDC1	1.80667795224065e-16	0.015	-0.024	0.039
RRM2	1.19667239941876e-23	0.019	-0.028	0.046
E2F7	1.06326612872157e-11	0.011	-0.022	0.033

References

1. R Mall, et al., Rgbm: regularized gradient boosting machines for identification of the transcriptional regulators of discrete glioma subtypes. *Nucleic acids research* **46**, e39–e39 (2018).
2. JH Friedman, Greedy function approximation: a gradient boosting machine. *Annals statistics* **29**, 1189–1232 (2001).
3. A Irrthum, L Wehenkel, P Geurts, , et al., Inferring regulatory networks from expression data using tree-based methods. *PloS one* **5**, e12776 (2010).
4. AA Margolin, et al., Aracne: an algorithm for the reconstruction of gene regulatory networks in a mammalian cellular context in *BMC bioinformatics.* (Springer), Vol. 7, p. S7 (2006).
5. MJ Alvarez, et al., Functional characterization of somatic mutations in cancer using network-based inference of protein activity. *Nat. genetics* **48**, 838–847 (2016).
6. A Sergushichev, An algorithm for fast preranked gene set enrichment analysis using cumulative statistic calculation. *BioRxiv* **https://doi.org/10.1101/060012**, 06–12 (2016).
7. A Subramanian, et al., Gene set enrichment analysis: a knowledge-based approach for interpreting genome-wide expression profiles. *Proc. Natl. Acad. Sci.* **102**, 15545–15550 (2005).
8. P Jaccard, Étude comparative de la distribution florale dans une portion des alpes et des jura. *Bull Soc Vaudoise Sci Nat* **37**, 547–579 (1901).
9. AM Taylor, et al., Genomic and functional approaches to understanding cancer aneuploidy. *Cancer cell* **33**, 676–689 (2018).
10. D Aran, M Sirota, AJ Butte, Systematic pan-cancer analysis of tumour purity. *Nat. communications* **6**, 1–12 (2015).

11. K Breuer, et al., Innatedb: systems biology of innate immunity and beyond—recent updates and continuing curation. *Nucleic acids research* **41**, D1228–D1233 (2012).
12. A Ruepp, et al., Corum: the comprehensive resource of mammalian protein complexes. *Nucleic acids research* **36**, D646–D650 (2007).
13. A Fabregat, et al., Reactome pathway analysis: a high-performance in-memory approach. *BMC bioinformatics* **18**, 142 (2017).
14. R Mall, R Langone, J Suykens, Kernel spectral clustering for big data networks. *Entropy* **15**, 1567–1586 (2013).
