# Supplementary material for: Network-based identification of key master regulators associated with an immune-silent cancer phenotype
Source: Brief Bioinform. 2021 May 13;22(6):bbab168. doi: 10.1093/bib/bbab168 (PMC8574720; doi:10.1093/bib/bbab168)
Supplement: Reply_to_Reviewers_ICR_v3_bbab168 [file reply_to_reviewers_icr_v3_bbab168.pdf]

Reply to Reviewers

Network-based Identification of Key Master Regulators associated with an Immune-Silent Cancer Phenotype

Mall et al

We would like to thank the reviewers for their insightful and thoughtful comments and suggestions. We have carefully evaluated and taken into consideration the suggestions for the final edits.

Below we provide a point-by-point answer to each of the reviewer suggestions.

Reviewer 1:

Comments to the Author
I am satisfied with the revisions made in this version of the manuscript, and **recommend its publication**.

Ans: We would like to thank the reviewer for appreciating our work and his **recommendation**. We are very grateful for his comments and suggestions that allowed us to vastly improve the manuscript.

Reviewer 2:

Comments to the Author
The authors have addressed my scientific concerns with the manuscript and have improved its readability and flow. However, issues related to the writing and still remain.

Ans: We would like to thank the reviewer for his recommendations and technical suggestions which allowed us to improve the technical quality of the manuscript.

Q1. There remain small grammar errors throughout the manuscript, primarily relating to subject-verb agreement (i.e. using singular verbs with plural subjects and the reverse). An

example of this can be found in the abstract: “These TRs, referred as Master Regulators (MR), is unique to immune-silent and immune-active tumors respectively.”

Ans: We are grateful to the reviewer for pointing out these grammatical issues. In the revised version of the manuscript, we have made the adequate changes.

Q2. Figure 2 is a welcome resource, as the ICR-H, ICR-L, ICR-M, ICR-D, ICR-E, ICR-N nomenclature is hard to keep track of mentally and makes the manuscript a difficult read. The authors should clarify in the introduction that ICR-neutral is a cancer type category related to ICR-E and ICR-D, rather than a sample category related to ICR-H and ICR-L, as this was not clear upon rereading the manuscript. Overall, the authors have done a good job outlining what are otherwise very confusing categories and providing figures and tables that the readers can refer to as they read the manuscript.

Ans: We would like to thank the reviewer for his recommendation and appreciation of our efforts to simplify the nomenclature used in the manuscript. In the revised manuscript, we have highlighted in the introduction that ICR-Neutral is a cancer type category where no significant prognostic value (w.r.t survival) is observed for the ICR phenotype (Roelands et al. 2020).

Q3. The manuscript still contains extraneous information that takes away from the main message. For instance:

A. Paragraph 1, column 2, page 6: “We obtained a total of 661, 542, 452, 342, 437, 150, 560 and 236 consensus MRs for ICR-E cancers BLCA, BRCA, HNSC, LIHC(Liver hepatocellular carcinoma), SARC(Sarcoma), SKCM, STAD (Stomach adenocarcinoma) and UCEC (Uterine Corpus Endometrial Carcinoma) respectively. Similarly, we obtained a total of 616, 453, 481 and 327 consensus MRs for ICR-D cancers LGG, KIRC (Kidney renal clear cell carcinoma), PAAD (Pancreatic adenocarcinoma) and UVM (Uveal Melanoma) respectively. Supp. Figure S1e highlights the MRs identified using the different MRA pipelines as ‘Venn’ diagrams for each of the 12 cancers of interest.” Why is the number of TFs important to the point it needs to be mentioned in the text? Can’t this entire section be replaced with a reference to a Supplementary Figure?

B. Paragraph 2, column 2, page 7: By ranking “ICR-E cancers based on the difference in their median activity in the ICR-H versus ICR-L samples per cancer tissue”, it is self-apparent that each of the “top 10 MRs had predominantly high activity in the ICR-H samples and relatively much lower activity in the ICR-L samples.” Similar can be said for the description in ICR-D cancers. This entire paragraph could be distilled into 1-2 sentences and should instead focus on the meaning of the results. The conclusion sentence of this paragraph, “Thus, the 19 MRs (highlighted in ‘grey’ in Figures 4A and 4B) which were shared across all the 12 cancers of interest (both ICR-E and ICR-D cancers) were all specific to the ICR-H phenotype,” seems like an

important finding, but I do not see any “grey highlighting” in Figure 4A and 4B, making it difficult to interpret.

Ans: We would like to thank the reviewer and thank the reviewer for very useful suggestions. We have moved the part about the number of TFs for each cancer type into Supplementary and have provided a reference for the same in the main manuscript.

We have simplified paragraph 2 on Page 7 into a couple of sentences as per reviewer suggestion and have highlighted the 19 MRs which are shared across all the 12 cancers of interest that are specific to the ICR-High phenotype. Moreover, we have highlighted the 19 MRs in Figures 4A and 4B as rightly pointed out by the reviewer.

Q4. It is still difficult to determine the logic in many of the comparisons. Why are the authors looking for MRs shared across ICR-H in ICR-E and ICR-H in ICR-D? What does it mean if they are shared in both ICR-E and ICR-D categories? What is the biological significance of this? The authors have improved on this by adding sentences at the start of each section, however they need to be more explicit in relating their findings back to the overall message. Another example: How are the MRs “validated” in ICR-N cancers? Validation typically involves comparison to a gold standard and it’s unclear why the authors think ICR-N cancers represent a gold standard.

Ans: We would like to thank the reviewer for his suggestions and useful comments. In the manuscript, we now clarify the motivation for our analysis and its biological significance. We state: “As a first step, we aimed at identifying the most conserved MRs characterizing the two opposite immune phenotypes (ICR-H and ICR-L) within each prognostic cancer category (ICR-E and ICR-D). We then compare the lists of the identified MRs between the 8 ICR-E and the 4 ICR-D cancers. We found 44 MRs differentially activated between ICR-H and ICR-L phenotypes but common to all the 8 ICR-E cancers as observed in Figure 4A. Similarly, we identified 29 MRs common to all the 4 ICR-D cancers as depicted in Figure 4B. Interestingly, we observe each of these MRs has high positive median activity in ICR-H samples and low negative median activity in ICR-L samples and thus is considered to be specific to the ICR-H phenotype. From Figures 4A and 4B, we determined 19 MRs (highlighted in `grey' in Figures 4A and 4B), which were shared across all the 12 cancers of interest (both ICR-E and ICR-D cancers) and were all specific to the ICR-H phenotype.

This observation indicated that 1) the dominant features characterizing the two opposite immune phenotypes (ICR-H and ICR-L) resulted in the upregulation of MRs related to high immune activity rather than MRs capturing immune-exclusion, and 2) these MRs are shared independently of the prognostic connotation of immunologic activity (ICR)” in the **Consensus MRs across 12 ICR Prognosticated Cancers** (see Page 6) subsection of **Experimental Results**.

Moreover, in the **Enrichment Analysis** (see Page 8) subsection of **Experimental Results**, we say “Once we had identified the MRs that were specific to the ICR-H (155 MRs) and ICR-L (57 MRs)

phenotypes across all the 12 cancer subtypes of interest, we performed downstream (enrichment) analysis using ConsensusPathDB" and "Interestingly, we observed that the majority of the top significantly enriched pathways are hallmark pathways of immune engagement (Bedognetti et al. 2016), justifying the ICR-H phenotype, where the high activity of these MRs indicated active immune engagement and at least a partial rejection of the cancer tissue (Wang, Bedognetti, and Marincola 2013)". This provides an additional biological significance of looking for shared MRs specific to the ICR-H phenotype across the ICR-E and ICR-D cancer categories.

With regards to "validation", validation can be referred to as 1) a comparison with a gold standard (in case a method is novel and a gold standard exists), but might also refer to 2) the validation of the findings obtained by a certain method using an external and unseen dataset (i.e. a dataset not included to obtain the findings). Since a gold standard does not exist w.r.t phenotype of interest, we take a consensus of 4 different master regulator analysis (MRA) pipelines to enhance the robustness of the proposed framework. We then replicate the major findings i.e. the MRs characterizing the ICR categories have similar activity patterns, as in ICR-E and ICR-D cancers, in the set of ICR-N cancers as well as an additional set of datasets obtained from the PRECOG repository. This is attributed to a successful validation/replication of the proposed approach.

In the manuscript, we stated: "We observed that the MRs, which were specific to the ICR-L phenotype (55 out of 57) had predominantly high activity patterns in all ICR-L samples independent of the type of cancer, whereas they had low activity patterns in the majority of the ICR-H samples for all the 20 ICR-N cancers in TCGA (see Figure 8A and Supp. Table S12 for statistical significance). Similarly, for the MRs associated with the ICR-H phenotype, we observed that a majority of these MRs (145 out of 155) had high activities in the ICR-H samples while they had negative activities in the majority of the ICR-L samples as demonstrated in Figure 8A (see Supp. Table 12 for statistical significance)" in the **Validation of MRs for ICR-N cancers & PRECOG datasets** subsection in **Experimental Results**.

Reviewer: 3

Comments to the Author
The authors carefully answered my questions and revised the manuscript. So, I **recommend this for publication.**

Ans: We are very thankful to the reviewer for their comments which enabled us to enhance the quality of the manuscript and appreciate his **recommendation** of our work.

References

Bedognetti, Davide, Wouter Hendrickx, Michele Ceccarelli, Lance D. Miller, and Barbara Seliger. 2016. “Disentangling the Relationship between Tumor Genetic Programs and Immune Responsiveness.” *Current Opinion in Immunology* 39 (April): 150–58.
Roelands, Jessica, Wouter Hendrickx, Gabriele Zoppoli, Raghvendra Mall, Mohamad Saad, Kyle Halliwill, Giuseppe Curigliano, et al. 2020. “Oncogenic States Dictate the Prognostic and Predictive Connotations of Intratumoral Immune Response.” *Journal for Immunotherapy of Cancer* 8 (1). https://doi.org/10.1136/jitc-2020-000617.
Wang, Ena, Davide Bedognetti, and Francesco M. Marincola. 2013. “Prediction of Response to Anticancer Immunotherapy Using Gene Signatures.” *Journal of Clinical Oncology: Official Journal of the American Society of Clinical Oncology* 31 (19): 2369.
